# Modular Synthesis of Organoboron Helically Chiral Compounds: Cutouts from Extended Helices

**DOI:** 10.1002/anie.202014138

**Published:** 2021-01-26

**Authors:** Julian Full, Santosh P. Panchal, Julian Götz, Ana‐Maria Krause, Agnieszka Nowak‐Król

**Affiliations:** ^1^ Institut für Anorganische Chemie Universität Würzburg Am Hubland 97074 Würzburg Germany; ^2^ Institut für Organische Chemie and Center for Nanosystems Chemistry Universität Würzburg Am Hubland 97074 Würzburg Germany

**Keywords:** chirality, circular dichroism, fluorescence, helicene, organoboron

## Abstract

Two types of helically chiral compounds bearing one and two boron atoms were synthesized by a modular approach. Formation of the helical scaffolds was executed by the introduction of boron to flexible biaryl and triaryl derived from small achiral building blocks. All‐ortho‐fused azabora[7]helicenes feature exceptional configurational stability, blue or green fluorescence with quantum yields (*Φ*_fl_) of 18–24 % in solution, green or yellow solid‐state emission (*Φ*_fl_ up to 23 %), and strong chiroptical response with large dissymmetry factors of up to 1.12×10^−2^. Azabora[9]helicenes consisting of angularly and linearly fused rings are blue emitters exhibiting *Φ*_fl_ of up to 47 % in CH_2_Cl_2_ and 25 % in the solid state. As revealed by the DFT calculations, their P–M interconversion pathway is more complex than that of **H1**. Single‐crystal X‐ray analysis shows clear differences in the packing arrangement of methyl and phenyl derivatives. These molecules are proposed as primary structures of extended helices.

## Graphical abstract

Configurationally stable and flexible organoboron helicenes consisting of either all‐*ortho*‐fused or a combination of angularly and linearly fused rings were synthesized by a modular approach from small achiral building blocks. A concept towards extended organoboron helices comprising both types of helically chiral compounds as primary substructures is presented.

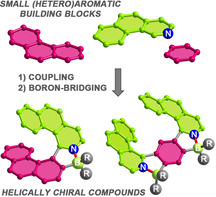

## Introduction

In recent years, chirality emerged as a central concept in the field of π‐conjugated compounds. Rapid progress in synthetic methodology of polycyclic aromatic hydrocarbons (PAHs) and nanobelts^[1]^ contributed to the development of a large variety of curved, contorted, and bent congeners.^[2]^ Interest in these compounds is motivated by their unique solid‐state packing, dynamic nature, and chiroptical properties of configurationally stable derivatives. Tuning of their properties is achieved mainly by substitution of their periphery with functional groups or, more recently, incorporation of heptagons and octagons.^[3]^ In general, the performance of all‐carbon PAHs can be improved by utilization of heteroatoms. Introduction of main group elements into PAHs entails significant perturbation of their electronic structures. Polycyclic heteroaromatics with fine‐tuned properties are explored as functional chromophores and charge transport materials. Such structural variation can also be used to achieve compounds with attractive features for coordination chemistry and catalysis.^[4]^

Primary representatives of chiral PAHs are screw‐shaped molecules called helicenes.[^4d^, ^5^] In materials science, they were studied in the context of their chirality‐determined organization in the solid state and its implications for charge transport in transistor and photovoltaic devices.^[6]^ They were also identified as promising compounds for chiral light emission, since they exhibit high differential emission of right‐ and left‐handed circularly polarized (CP) light quantified by the dissymmetry factor.^[7]^ Yet, for application as CP luminescence emitters, for instance in CP‐organic light emitting diodes (CP‐OLEDs), high dissymmetry factors are not sufficient. Organic materials should also show intense emission, preferably at high concentration or in the solid state. However, intersystem crossing typically lowers *Φ*_fl_ of helicenes,^[8]^ hence limiting their potential use in chiral optoelectronics.

π‐Conjugated boron compounds have received recognition for their outstanding optical properties and are intensively studied in OLED devices.[^9^, ^10^] Embedding boron into π‐conjugated scaffolds can provide materials with high electron affinity, electron mobility, and photovoltaic performance.[^10^, ^11^] Moreover, the versatility of B−N dative bonds enables the construction of stimuli‐responsive materials and dynamic systems.^[12]^ Thus, merging benefits of boron with chirality could give rise to materials with unique characteristics and improved properties versus all‐carbon analogues.

Although a plethora of organoboron molecules have been synthesized to date,[^9^, ^10^, ^11^, ^12^, ^13^, ^14^] the availability of helically chiral congeners with boron in the π‐conjugated core is still limited. Three‐coordinate boron‐fused helicenes were synthesized by Hart reaction and boron‐assisted demethylative cyclization as the key steps,^[15]^ a tandem bora‐Friedel–Crafts‐type reaction,^[16]^ intramolecular Yamamoto coupling of triarylborane,^[17]^ or intramolecular electrophilic borylation.^[18]^ Likewise, four‐coordinate boron helicenes are rare. In addition to chiral *O*‐BODIPYs and *O*‐aza‐BODIPYs with boron on the inner helicene rim,^[19]^ only few other organoboron helicenes have been reported. Boron‐bridging of [4]‐ and [6]helicenes with one or two flanking pyridine units^[20]^ elongated the framework by two or four fused rings, whereas configurational stability in azabora[5]helicenes was achieved by substitution of terminal positions with sterically demanding groups.^[21]^

**Figure 1:**
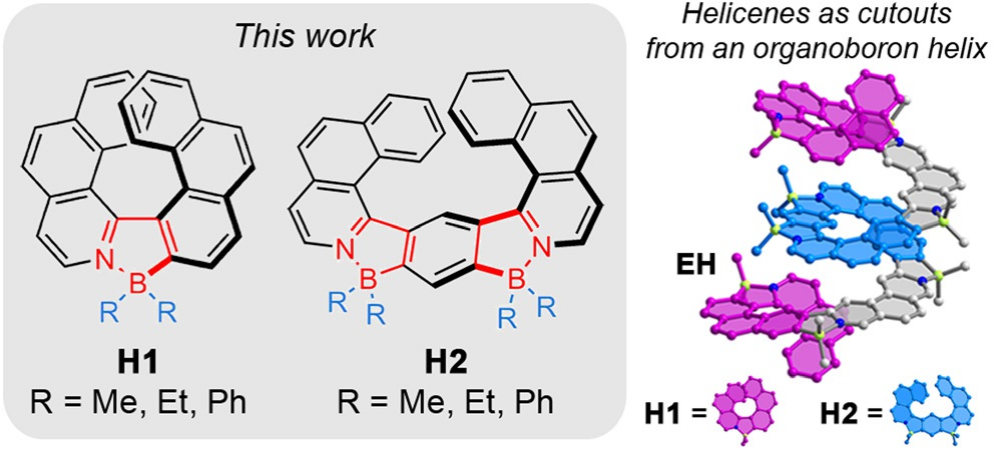
Structures of **H1** and **H2. EH** is an example of an extended helix incorporating both types of helicenes. For simplicity, only (*P*)‐ and (*P*,*P*)‐steroisomers are shown. The hydrogen atoms have been removed.

Our objective is the synthesis of long helicenes consisting of multiple boron atoms. The attractiveness of such extended structures lies beyond the structural curiosity. These entities should display large circular dichroism, efficient symmetry‐breaking spin transport,^[22]^ and allow studies of exciton transport pathways in discrete molecules. Access to such elongated structures is, however, limited due to synthetic limitations. There are only few reports on long well‐defined helicenes^[23]^ with the record number of 19 fused rings for oxahelicenes.^[24]^ To construct organboron helices, we propose a modular approach in which flexible oligoaryl precursors are prepared in a convergent synthesis from small achiral building blocks. Lewis acidic boron is introduced as “glue” to these species to join two or more subunits into fully fused scaffolds by formation of dative bonds with nitrogen or other heteroatoms. Noteworthy, chirality cannot be ensured by bulky substituents at sterically hindered positions of the substructures in this case, nor can the boron atoms be included only in the terminal parts of the helical backbone. The construction of fully *ortho*‐fused helices is energetically costly due to introduced steric strain. A reasonable means to facilitate the closure of azaborole rings would be incorporation of *meta*‐fused or a combination of both *ortho*‐ and *meta*‐fused units. Thus, we herein present the synthesis and properties of two types of molecules differing in the fusion point: fully π‐conjugated all‐*ortho*‐fused azabora[7]helicenes **H1** and azbora[9]helicenes **H2**, which embody both angularly and linearly fused rings, as primary substructures of organoboron helices, such as **EH** in Figure 1. As we will demonstrate, a combination of a non‐planar geometry of helicenes with boron has a synergistic effect on the emission of these emitters both in solution and in the solid state.

## Results and Discussion

**Scheme 1:**
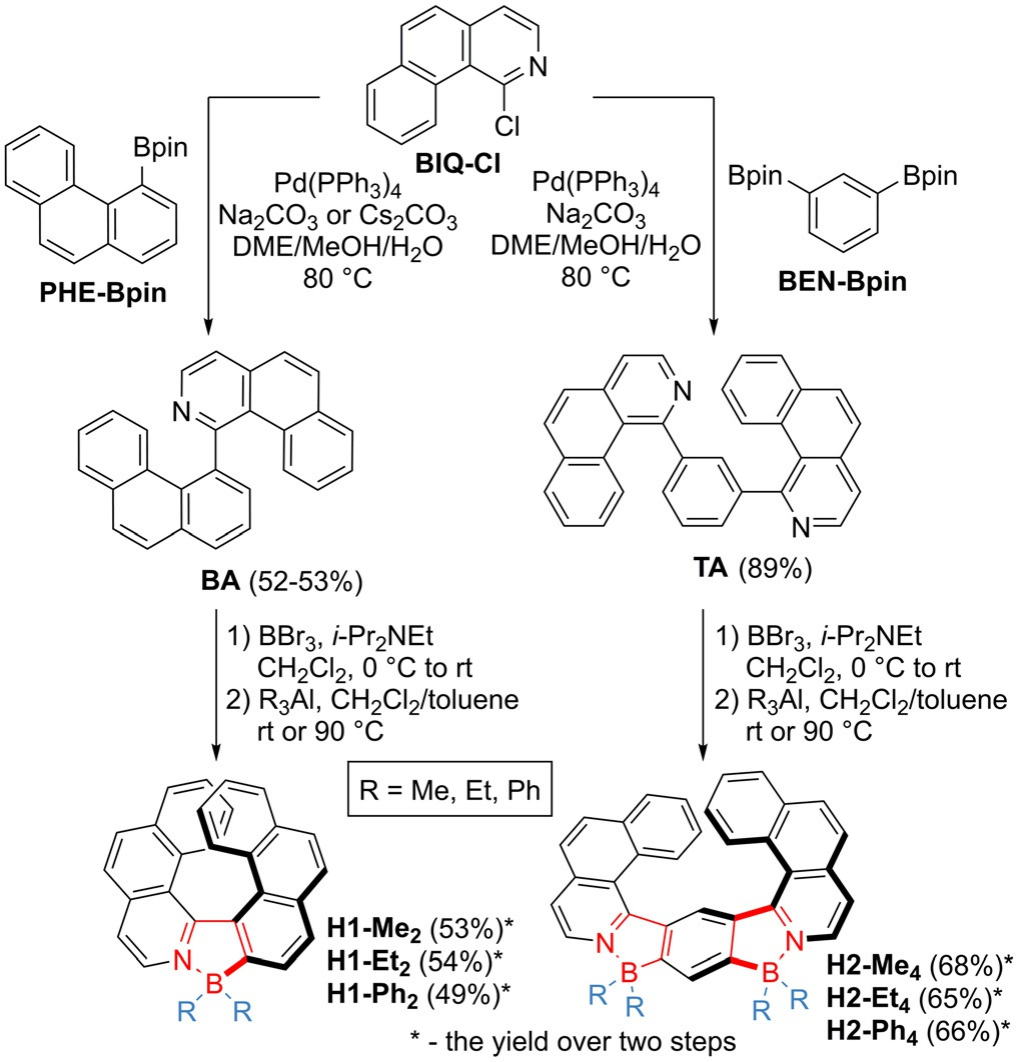
Modular synthesis of **H1** and **H2**.

**Synthesis**. Biaryl **BA** and triaryl **TA** were synthesized by cross‐coupling of 1‐chlorobenzo[*h*]isoquinoline (**BIQ‐Cl**) with borylated phenanthrene (**PHE‐Bpin**) and benzene (**BEN‐Bpin**) derivatives (Scheme 1). **BEN‐Bpin** was prepared by Miyaura borylation from commercially available 1,3‐dibromobenzene. The syntheses of the other two building blocks are more demanding. **PHE‐Bpin** was synthesized in seven steps from 2‐bromobenzaldehyde and 2‐methoxyphenylboronic acid. Cross‐coupling thereof afforded formyl‐substituted biphenyl, which was converted into the corresponding alkyne via Corey–Fuchs reaction. The subsequent Pt‐catalyzed ring closure produced methoxy‐phenanthrene. The following cleavage of the methyl ether, synthesis of a pseudohalide, and Suzuki–Miyaura reaction furnished **PHE‐Bpin. BIQ‐Cl** could be obtained in five steps. The synthesis started from coupling of 3‐bromo‐4‐methylpyridine with 2‐formylphenylboronic acid, followed by base‐promoted cyclization to benzo[*h*]isoquinoline (**BIQ**). Oxidation of **BIQ**, rearrangement of *N*‐oxide to the corresponding lactam, and, finally, chlorination thereof with POCl_3_ afforded **BIQ‐Cl**. For reproducibility and ease of purification, it is advised to perform the last three reactions in a stepwise manner (method B, Supporting Information) rather than in one pot (method A). The detailed synthesis of the small building blocks is presented in the Supporting Information. To execute the introduction of boron atoms into these intermediates, we adapted the method reported by Murakami^[25]^ with some modifications. According to this protocol, **BA** and **TA** were reacted with BBr_3_ in the presence of *i*‐Pr_2_NEt to yield complexes **H1‐Br_2_** and **H2‐Br_4_**, respectively. The synthesis was accomplished by the exchange of the bromide with alkyl or aryl ligands in overall yields of 7–8 % for **H1** (10 steps) and 30–32 % for **H2** (8 steps). Whereas substitution with Me and Et could be performed under mild conditions, introduction of Ph groups required elevated temperature. Triorganylaluminum reagents proved superior to diorganylzinc complexes for this transformation. Not only was the reaction of **H1‐Br_2_** with Et_2_Zn lower‐yielding than the analogous reaction with Et_3_Al, but it was also more sluggish and had to be performed at higher temperature (see the Supporting Information). All compounds except **H2‐Et_4_** feature excellent stability against light, moisture and air. **H2‐Et_4_**, on the other hand, decomposed over time. For this reason, its further characterization was not carried out.

**Solid state structural analysis**. Single crystals of racemic **H1‐Me_2_** and **H2‐Me_4_** suitable for X‐ray analysis were obtained by slow evaporation of chloroform solutions, and those of **H1‐Ph_2_** and **H2‐Ph_4_** by diffusion of hexane into CH_2_Cl_2_ solutions. **H1‐Me_2_** and **H2‐Me_4_** crystallized in the *P*2_1_/*n* space group, the other two in the *P̄*1 space group. The B−N bond lengths of 1.597(3)–1.612(6) Å in the azaborole rings confirm strong Lewis pair interactions (Figures S63–S66 in the Supporting Information). In the solid state, compounds **H1** and **H2** adopt helical conformations. The sums of the five dihedral angles for the inner helicene rim (*ϕ*) of **H1‐Me_2_** and **H1‐Ph_2_** are 94.2° and 88.4°, respectively, which are intermediate values between those of phospha‐ and sila[7]helicenes (95–100°)^[26]^ and other hetero[7]helicenes (79–88°).^[27]^ The distortion is largely determined by the geometry of the five‐membered rings closely related to the type of a heteroatom. The angles between two formal C=C double bonds of azaborole rings are approximately 38°, large enough to ensure a substantial overlap of terminal rings and, in turn, excellent configurational stability of **H1**.

**Figure 2:**
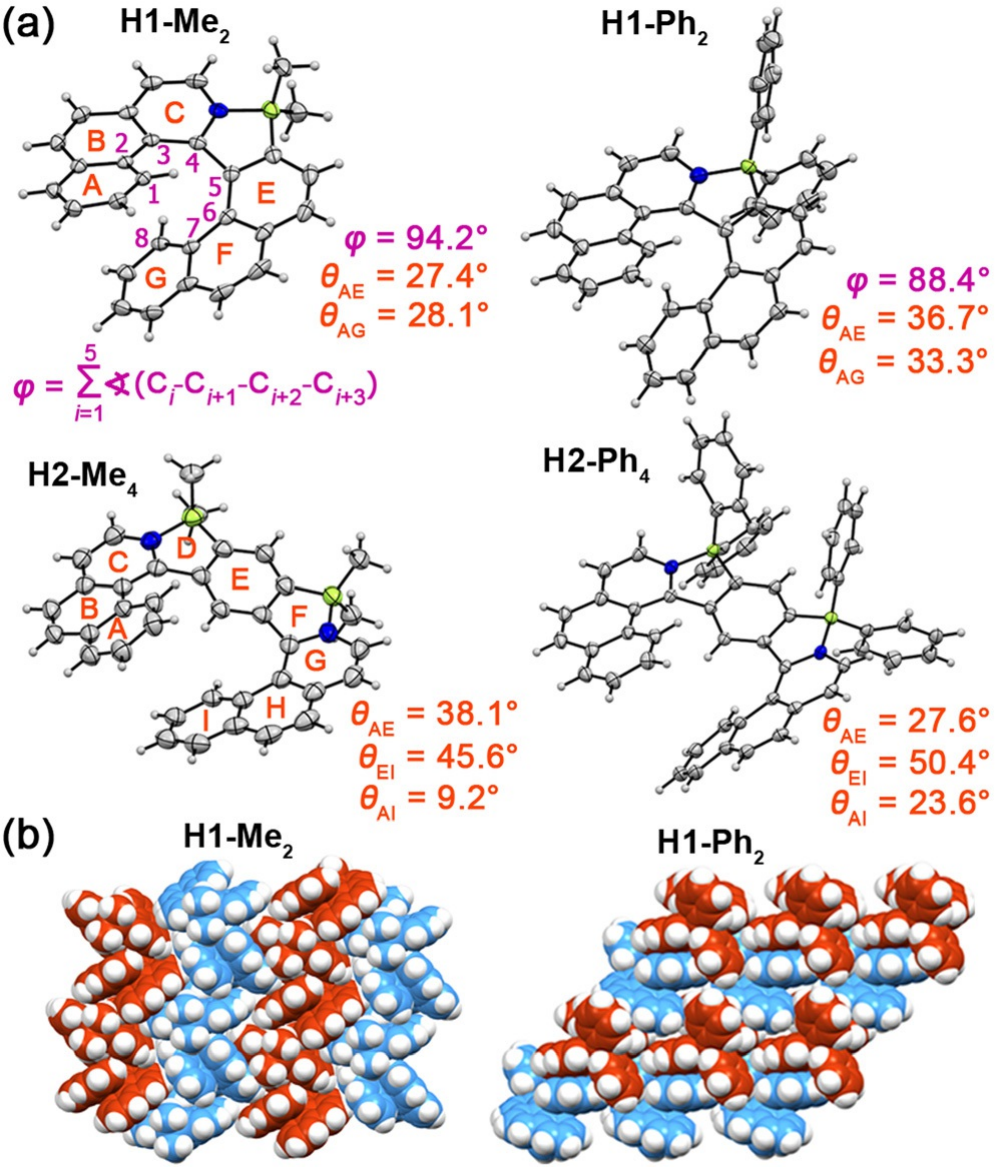
a) Molecular structures of **H1‐Me_2_**, **H1‐Ph_2_**, **H2‐Me_4_**, and **H2‐Ph_4_**,^[47]^ determined by X‐ray analysis at 100 K. ORTEP drawings are shown with 50 % probability. Only (*P*)‐enantiomers are shown. b) Packing arrangements of **H1‐Me_2_** and **H1‐Ph_2_**. (*P*)‐ and (*M*)‐enantiomers are colored in maroon and blue, respectively.

The dihedral angle between the mean planes of terminal rings in **H1‐Me_2_** is 28.1° (Figure 2), smaller than in other hetero[7]helicenes. A slightly larger *θ*_AG_ (33.3°) was observed in **H1‐Ph_2_**, which is comparable to that in pristine carbo[7]helicene (32.3°).^[28]^ Such small splay angles indicate enhanced intramolecular π–π interactions in both molecules. The angles defined by rings A–E (formally azabora[5]helicene) are 27.4° and 36.7° (Figure 2). The corresponding angles in **H2** molecules are generally larger. In **H2‐Me_4_** one BIQ wing is more strongly bent than the other unit (*θ*_AE_ and *θ*_EI_ of 38.1 and 45.6°). **H2‐Ph_4_** is even more distorted (*θ*_AE_ and *θ*_EI_ of 27.6 and 50.4°), which results in a larger *θ* between the terminal rings (*θ*_AI_ of 23.6° vs. 9.2° for **H2‐Me_4_**). Since the helical cores of the optimized geometries are almost symmetrical, these differences must originate from the crystal packing modes.

In the packing arrangements of all four helicenes, stacks of (*P*)‐ and (*M*)‐enantiomers could be observed. The molecules are arranged in a slipped fashion forming stacks with interplanar distances of 3.35–3.59 Å. **H1‐Me_2_** and **H2‐Me_4_** arrange in a sandwich herringbone pattern through C−H⋅⋅⋅π interactions with adjacent dimers. **H1‐Ph_2_** and **H2‐Ph_4_** share a different packing arrangement. The isomers are packed in an alternating fashion forming sheet structures with multiple C−H⋅⋅⋅π interactions also involving Ph rings (Figure S67 in the Supporting Information).

**Figure 3:**
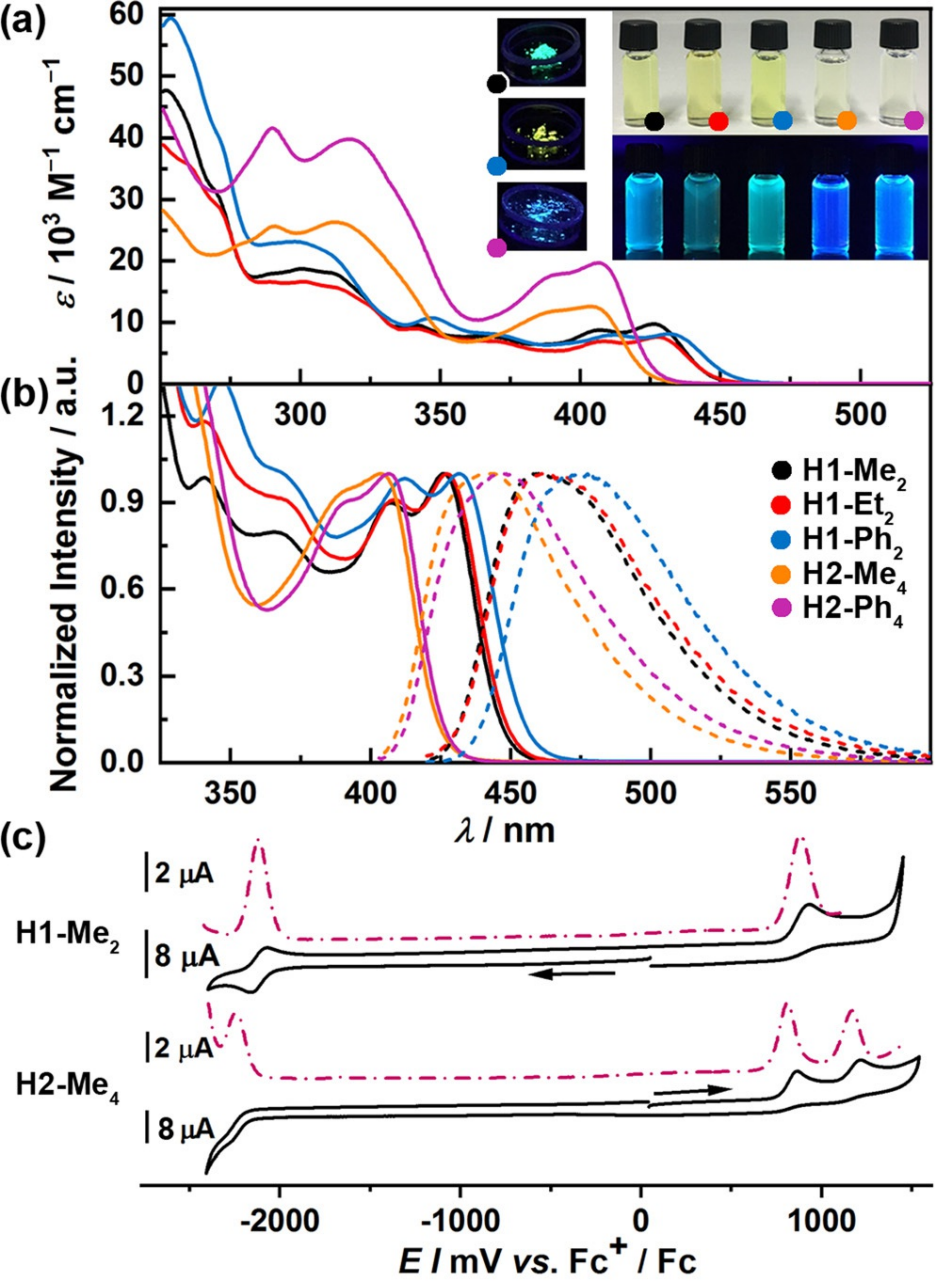
a) Absorption spectra of **H1** and **H2** in CH_2_Cl_2_. Inset right: Photographs of their solutions in CH_2_Cl_2_ under visible (top) and UV light (bottom). Inset left: Photographs of the powders of **H1‐Me_2_**, **H1‐Ph_2_**, and **H2‐Ph_4_** under UV light. b) Normalized absorption (solid lines) and emission (dashed lines) spectra of **H1** and **H2** in CH_2_Cl_2_. c) Cyclic (solid lines) and differential pulse (dash‐dotted lines) voltammograms of **H1‐Me_2_** and **H2‐Me_4_**.

**Absorption and emission properties**. The photophysical data are summarized in Table S1 (Supporting Information). **H1** show moderate molar absorption coefficients (*ϵ*) (7.6–9.7×10^3^ m ^−1^ cm^−1^). The lowest‐energy absorption bands of **H1** are centered at 426–432 nm with well‐resolved vibronic progressions at 407–412 nm and correspond to the yellow color of CH_2_Cl_2_ solutions (Figure 3 a). Absorption maxima of **H1** are bathochromically shifted vs. all‐carbon analogues^[29]^ and related hetero[7]helicenes.[^26^, ^27^] *λ*_abs_ of the compounds bearing two boron atoms are blue‐shifted to 404–406 nm. This pronounced shift is accompanied by an increase in intensity (*ϵ* of up to 19.7×10^3^ m ^−1^ cm^−1^). In contrast to **H1**, the fine structure is almost entirely lost. The significant hypsochromic shift vs. **H1** is likely due to a somewhat disrupted conjugation along the helical core. Since **H2** features high flexibility (see below), it is possible that various conformers coexist in solution differing in the effective π‐conjugated pathway. The HOMOs and LUMOs of **H1** are delocalized over the entire helicene cores with somewhat larger coefficients on PHE and BIQ moieties, respectively (Figure S74 in the Suppporting Information). The HOMOs of **H2** involve the whole π‐conjugated systems with larger coefficients at the pyridine and central benzene rings, and small contributions from the Ph substituents for **H2‐Ph_4_**. The LUMO and LUMO+1 of **H2** are more or less uniformly delocalized over both BIQ and BEN moieties. According to the time‐dependent density functional theory (TD‐DFT) calculations at the CAM‐B3LYP^[30]^‐D3BJ^[31]^/def2‐TZVP^[32]^ level (solvent CH_2_Cl_2_, PCM model) the lowest energy absorption bands of **H1** mainly correspond to the HOMO→LUMO transitions (84 %), while those of **H2** are superpositions of two transitions and are predominantly attributed to the HOMO→LUMO (76–80 %, oscillator strength *f*≈0.45), and HOMO→LUMO+1 (≈70 %, *f* ≈0.72–0.10) transitions (Figure S75 and Tables S3–S6 in the Supporting Information) so that very little charge transfer is to be expected. The compounds show blue (**H1‐Me_2_**, **H1‐Et_2_**, and both **H2**) or green (**H1‐Ph_2_**) fluorescence with maxima at 459–477 nm (Figure 3 b), which translates to Stokes shifts of 1700–1800 cm^−1^ for **H1‐Me_2_** and **H1‐Et_2_**, and 2200–2250 cm^−1^ for **H1‐Ph_2_**, and **H2** compounds. *λ*_fl_ of **H2** are, like the absorption bands, blue‐shifted versus emission maxima of **H1**. The emission spectra are devoid of vibronic structures. Fluorescence quantum yields (*Φ*_fl_) fall in the range of 18–24 % and are markedly higher compared to carbohelicenes consisting of only six‐membered rings,^[33]^ whereas *Φ*_fl_ of structurally similar compounds strongly depend on the atom at the fusion point of a central five‐membered ring (from 0.1 to 23 % for heteroatoms and up to 40 % for carbon).[^26^, ^27^, ^29^, ^34^] **H2** are highly emissive with *Φ*_fl_ of 43–47 %.

Absorption of spin‐coated films is slightly red‐shifted (Figure S68 and Table S1 in the Supporting Information), probably due to somewhat increased intramolecular interactions in the solid state, with the absorption maxima of **H1** located between 433 and 438 nm and of **H2** at 402 nm. In general though, the line shapes resemble those of spectra in solution. Only small variations in the intensity ratios of the 0–0 to 0–1 vibronic transitions of the S_0_→S_1_ transition from ca. −5 % for **H2‐Me_4_** to +9 % for **H1‐Ph_2_** could be observed. Emission was measured for amorphous powder samples of **H1** and **H2**. The fluorescence spectra are presented in Figure S69, while the images of the powders under visible and UV irradiation are shown in Figures 3 a and S70 in the Supporting Information. **H1‐Me_2_**, **H1‐Ph_2_**, and **H2‐Ph_4_** show impressive *Φ*_fl_ values of 17, 23, and 25 %, respectively.^[35]^ To our knowledge, these *Φ*_fl_ values are among the highest quantum yields reported for helicenes to date.^[36]^ However, emission of **H1‐Et_2_** and **H2‐Me_4_** is substantially weaker (3 % and 8 %, respectively). As opposed to **H2**, showing blue fluorescence both in solution and in the powder with only small shifts of the emission spectra, the spectra of powder samples of **H1** are red‐shifted by approximately 2000 cm^−1^ for both alkyl derivatives and almost 3000 cm^−1^ for **H1‐Ph_2_** as compared to their spectra in CH_2_Cl_2_. These pronounced spectral shifts result in a change of the emission color from blue to green and green to yellow, respectively.

Essentially, the *Φ*_fl_ values of **H1‐Me_2_** and **H1‐Ph_2_** do not decrease upon going from solution to the solid state. In contrast to other popular emitters, such as BODIPY^[37]^ or perylene bisimide (PBI) dyes,^[38]^ these organoboron helicenes do not undergo aggregation‐caused quenching of fluorescence. Whereas for PBIs, extensive molecular engineering via introduction of voluminous substituents is necessary in order to retain high emission properties in the solid state,^[39]^ we could achieve this for **H1‐Me_2_** and **H1‐Ph_2_** without any special treatment, since their inherent non‐planar geometry effectively reduces intermolecular π–π interactions. In addition, the advantage of this molecular design manifests itself in the fact that the change in the emission color of **H1** could be obtained by simply replacing Me with Ph substituents, hence without any modification of the π‐conjugated core.

**Electrochemistry**. The electrochemical behavior of **H1** and **H2** was investigated by cyclic voltammetry (CV) and pulse techniques in CH_2_Cl_2_ in the presence of Bu_4_NPF_6_ as a supporting electrolyte and calibrated versus ferrocenium/ferrocene (Fc^+^/Fc). As shown in Figures 3 c and S71 in the Supporting Information, all **H1** compounds exhibit one reversible reduction wave at −2.11–−2.13 V for alkyl derivatives. The reduction potential of **H1‐Ph_2_** is anodically shifted by ca. 0.1 V. The effect of substituents on boron is more pronounced for oxidation. Exchange of alkyl with Ph substituents results in an anodic shift of ca. 0.2 V. For **H1‐Et_2_**, the second oxidation at +1.57 V could be recorded. The differences in redox potentials are rather small. Thus, the band gaps differ only slightly, which coincides with the shifts in the absorption spectra of these compounds. A voltammogram of **H2‐Me_4_** reveals two oxidation processes at +0.86 and +1.22 V, and one irreversible reduction at −2.24 V. On the contrary, only one oxidation (+1.08 V) and two reduction processes at −2.09 and −2.31 V were observed for the Ph congener.

**Figure 4:**
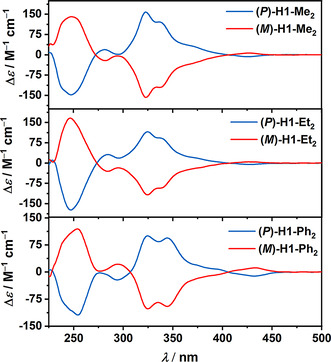
ECD spectra of **H1‐Me_2_**, **H1‐Et_2_**, and **H1‐Ph_2_** in CH_2_Cl_2_ (*c=*11–21 μm).

**Chiroptical properties**. Enantiomers of **H1‐Me_2_**, **H1‐Et_2_**, and **H1‐Ph_2_** were resolved by HPLC on a chiral stationary phase (for details see SI). As shown in Figure 4, their electronic circular dichroism (ECD) spectra recorded in CH_2_Cl_2_ revealed perfect mirror‐image relationships. The absolute configuration of the enantiomers was assigned by comparison of the experimental ECD with the TD‐DFT‐simulated ECD spectra (Figure S76 in the Supporting Information). Thus, the first and second fractions correspond to (*P*)‐ and (*M*)‐enantiomers, respectively. As expected, the ECD spectra of **(*P*)‐H1‐Me_2_** and **(*P*)‐H1‐Et_2_** have similar profiles, they differ, however, in intensity. Accordingly, their spectra exhibit positive Cotton effects (CEs) in the ranges of 272–405 nm (Δ*ϵ*=+158 m ^−1^ cm^−1^ at 323 nm; Δ*ϵ*= +19 m ^−1^ cm^−1^ at 282 nm) and 273–407 (Δ*ϵ*=+115 m ^−1^ cm^−1^ at 324 nm; Δ*ϵ*=+31 m ^−1^ cm^−1^ at 284 nm), respectively. Negative CEs are observed in the ranges of 229–272 (Δ*ϵ*=−148 m ^−1^ cm^−1^ at 248 nm) and 405 to ca. 450 nm (Δ*ϵ*=−7 m ^−1^ cm^−1^ at 426 nm) for **(*P*)‐H1‐Me_2_** and 231–273 (Δ*ϵ*=−173 m ^−1^ cm^−1^ at 247 nm) and 405 to ca. 450 nm (Δ*ϵ*=−6 m ^−1^ cm^−1^ at 428 nm) for **(*P*)‐H1‐Et_2_**. The ECD spectrum of **(*P*)‐H1‐Ph_2_** revealed a different profile to those of alkyl derivatives with negative ECD at 294 nm (Δ*ϵ*=−22 m ^−1^ cm^−1^) and a strong negative CE at 254 nm (Δ*ϵ*=−119 m ^−1^ cm^−1^). A positive CE appears in the range of 307–405 nm (Δ*ϵ*= +100 m ^−1^ cm^−1^ at 325 nm), and a weak negative CE between 405 and 459 nm (Δ*ϵ*=−11 m ^−1^ cm^−1^ at 432 nm). The intensities of the longest wavelength bands of all compounds are low and so are the corresponding anisotropy factors (*g*_abs_) (0.7×10^−3^–1.4×10^−3^). On the other hand, the strong ECD bands located at 323, 324, and 325 correspond to the highest |*g*_abs_| of 1.12×10^−2^, 9.1×10^−3^, and 7.6×10^−3^, respectively. In particular, **H1‐Me_2_** exhibits excellent chiroptical performance with |*g*_abs_| exceeding those of carbo[6]helicene,^[40]^ a number of multipoles,[^3a^, ^41^] and approaching |*g*_abs_| of helicene nanoribbons^[42]^ and double[8]helicene.^[43]^

**Figure 5:**
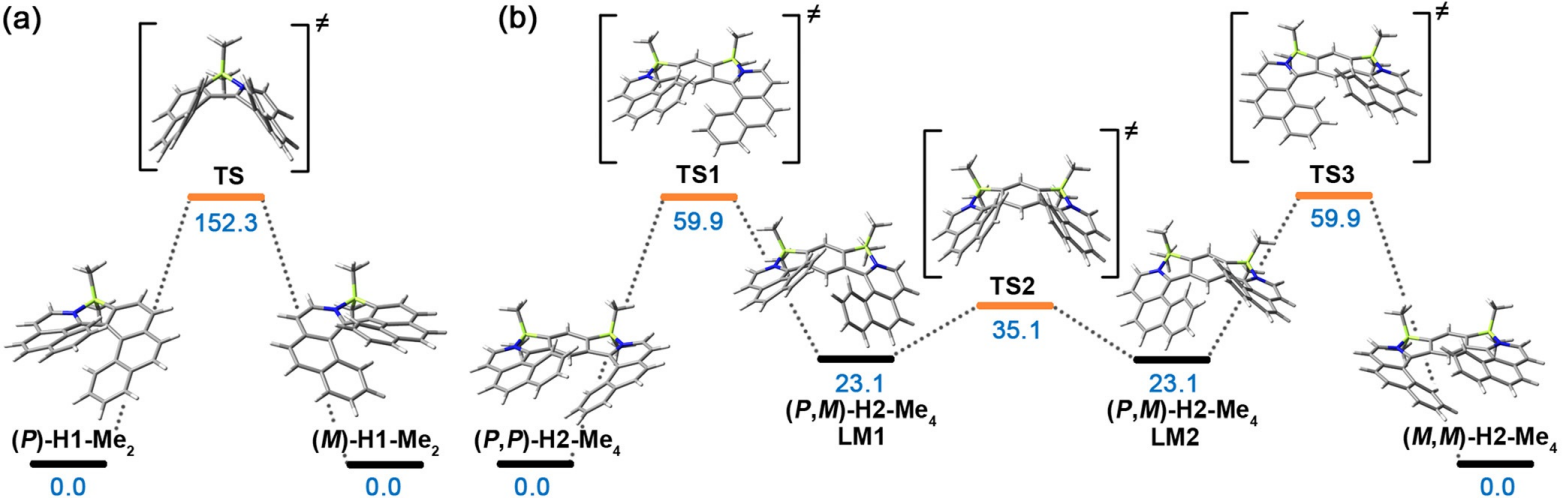
Interconversion pathways of a) **(*P*)‐H1‐Me_2_** and b) **(*P***,***P*** **)‐H2‐Me_4_** calculated at the B3LYP/def2‐SVP (solvent CH_2_Cl_2_, PCM model) level. The relative Gibbs free energies for the stationary points are given in kJ mol^−1^.

***P*** **–*M* interconversion**. As opposed to **H2** (see below), helicenes **H1** are configurationally stable. No racemization was observed for a solution of **(*P*)‐H1‐Me_2_** over a month at room temperature. According to DFT calculations (B3LYP‐D3BJ/def2‐SVP,^[28]^ solvent CH_2_Cl_2_, PCM model), the *P*–*M* interconversion of **H1‐Me_2_** proceeds via one transition state of *C*_1_ symmetry (Figure 5). The inversion barrier (Δ*G* ^≠^) is 152.3 kJ mol^−1^ (36.4 kcal mol^−1^) and is comparable to the configurationally stable hexahelicene (36.2 kcal mol^−1^).^[44]^ For comparison, Δ*G* ^≠^ for azabora[5]helicenes **H3** of 57.8 kJ mol^−1^ (13.8 kcal mol^−1^) (Figure S77 in the Supporting Information) is considerably lower and the formation of the configurationally stable helicene would require introduction of a bulky substituent into a sterically hindered position of the N‐heterocycle or an all‐carbon subunit. Thermal racemization of **(*P*)‐H1‐Me_2_** in 1,2‐dichlorobenzene at 180 °C was monitored by HPLC following the decay of the enantiomeric excess. The Gibbs free energy of activation for racemization was determined to be 142.6 kJ mol^−1^ (34.1 kcal mol^−1^), which corresponds to a racemization half‐life of 70.2 min at 180 °C and approximates to the calculated value. Such a high barrier indicates that the devices incorporating these materials would not be adversely affected by racemization during the fabrication process, even at relatively high temperatures. In contrast to **H1**, the interconversion of **H2** occurs via three transition states due to the presence of a hydrogen atom of the central benzene ring on the inner rim of **H2**. In principle, **H2** can be considered as two azabora[5]helicenes (**H3**) with one joint benzene ring, each undergoing *P*–*M* interconversion. In the first step, **(*P***,***P*** **)‐H2‐Me_4_** converts to a local minimum **(*P***,***M*** **)‐H2‐Me_4_** (**LM1**) with both BIQ on the same side of the benzene ring. The stable conformation is ca. 23.1 kJ mol^−1^ (5.5 kcal mol^−1^) lower in energy than **LM1**. The second process occurs via a transition state in which two BIQ moieties are in co‐facial arrangement. This process is accompanied by the lowest energy penalty. From this state, the molecule relaxes to a second local minimum **LM2**—an enantiomer of **LM1**. Finally, the molecule reaches a stable form **(*M***,***M*** **)‐H2‐Me_4_** via **TS3** which has an enantiomeric relationship with **TS1**. The activation barriers for **TS1**, **TS2**, and **TS3** are 59.9, 12.0, and 36.8 kJ mol^−1^ (14.3, 2.9, and 8.8 kcal mol^−1^), respectively. The first value is markedly smaller than a barrier of 100 kJ mol^−1^ (23.9 kcal mol^−1^) for carbo[5]helicene,^[45]^ which racemizes slowly at ambient temperature.^[46]^ Thus, the interconversion of **H2** occurs rapidly at room temperature, which prevents the resolution of the stereoisomers. Because both azabora[5]helicenes are a part of the same system, the interconversion of one of them affects the geometry of the second subunit and hence, the whole molecule. Nevertheless, the presence of the second BIQ unit leads to a negligible increase in Δ*G* ^≠^ (+2.1 kJ mol^−1^) as compared with **H3**. Accordingly, the limiting process in the interconversion of **H2** is defined by the interconversion of the azabora[5]helicene subunit.

## Conclusion

In summary, we have synthesized azabora[7]helicenes and azabora[9]helicenes as primary substructures of extended helical structures. These compounds were prepared by a conceptually simple modular approach in which the helical structure was obtained by boron‐bridging of conformationally flexible biaryl **BA** and triaryl **TA**. Configurational stability of angularly fused **H1** was achieved without any additional blocking groups at the terminal positions. Configurationally flexible **H2** is a new type of building block consisting of both angularly and linearly fused rings. **H1** feature moderate (high among helicenes) fluorescence quantum yields and superior chiroptical properties with |*g*_abs_| of up to 1.12×10^−2^. Excellent *Φ*_fl_ of 43–47 % were recorded in CH_2_Cl_2_ solution for **H2**. Intense fluorescence (*Φ*_fl_ of up to 25 %) was retained in the solid state for **H1‐Me_2_**, **H1‐Ph_2_**, and **H2‐Ph_4_** affording green, yellow, and blue emitters, respectively. Thus, introduction of boron into helical scaffolds provided helicenes with outstanding optical properties both in solution and in the solid state. These features along with high chemical and photostability make these fluorophores attractive for applications as pristine materials or (chiral) emissive dopants in polymer matrices in OLEDs, fluorescent solid‐state sensors, and fluorescent probes for bioimaging.

The flexibility of our synthetic approach opens up the opportunity to prepare heterohelices with precisely modulated properties. Incorporation of both types of units should facilitate modification of their helical pitch, the extent of the intersystem crossing and optical properties. Our current efforts are focused on the application of this concept to the synthesis of extended systems.

## Conflict of interest

The authors declare no conflict of interest.

## Supporting information

As a service to our authors and readers, this journal provides supporting information supplied by the authors. Such materials are peer reviewed and may be re‐organized for online delivery, but are not copy‐edited or typeset. Technical support issues arising from supporting information (other than missing files) should be addressed to the authors.

Supplementary
