# Supplementary material for: Modular Synthesis of Organoboron Helically Chiral Compounds: Cutouts from Extended Helices
Source: Angew Chem Int Ed Engl. 2021 Jan 26;60(8):4350–7. doi: 10.1002/anie.202014138 (PMC7898935; doi:10.1002/anie.202014138)
Supplement: Supplementary file 1 — Supplementary [file ANIE-60-4350-s001.pdf]

Angewandte Chemie
Eine Zeitschrift der Gesellschaft Deutscher Chemiker

Supporting Information

Modular Synthesis of Organoboron Helically Chiral Compounds: Cutouts from Extended Helices

*Julian Full[+], Santosh P. Panchal[+], Julian Götz, Ana-Maria Krause, and Agnieszka Nowak-Król**

anie_202014138_sm_miscellaneous_information.pdf

Table of Contents

1. Materials and Methods 2
2. Synthesis and Characterization of Organoboron Helicenes 3
3. NMR and MS spectra 10
4. Single crystal X-ray analysis 41
5. Optical and Electrochemical Properties 45
6. Resolution of Enantiomers 47
7. Interconversion Barrier 47
8. Computations 48
9. References 86

1. Materials and Methods

Synthesis: All reagents were purchased from commercial sources and used as received without further purification, unless otherwise stated. Reagent grade solvents were distilled prior to use. Column chromatography was performed on silica (silica gel, 230–400 mesh) and flash column chromatography on Interchim PuriFlash 450.

NMR Spectroscopy: ^{1}H, ^{13}C NMR spectra were recorded on a Bruker Avance 400 or an Avance III HD 400 spectrometers and were calibrated to the residual solvent signals. *J* values are given in Hz. The following abbreviations were used to designate multiplicities: s = singlet, d = doublet, t = triplet, dd = doublet of doublet, ddd = doublet of doublet of doublet, dm = doublet of multiplet, dt = doublet of triplet, td = triplet of doublet, m = multiplet, br s = broad singlet.

Mass Spectrometry: High-resolution mass spectra were obtained by electrospray ionization (ESI), atmospheric-pressure chemical ionization (APCI) or matrix-assisted laser desorption/ionization (MALDI). ESI and APCI spectra were recorded on an ESI micrOTOF Focus spectrometer from Bruker Daltonics. MALDI spectra were recorded on a Bruker Daltonics autoflex II LRF or a Bruker Daltonics ultrafleXtreme spectrometers. *Trans*-2-[3-(4-*tert*-butylphenyl)-2-methyl-2-propenylidene]malononitrile (DCTB) or 2',4'-dihydroxyacetophenone (DHAP) were used as MALDI matrices.

UV/Vis Absorption Spectroscopy in Solution: UV/Vis spectra were recorded on a Jasco V-770 UV/Vis spectrometer. All spectroscopy measurements were conducted with spectroscopic grade solvents from ACROS Organics. Conventional quartz cells (light path 1 cm) were used.

Absorption Spectroscopy in Thin Films: UV/Vis spectra in the solid state were recorded on a Lambda 950 UV/Vis spectrometer from PerkinElmer equipped with an integration sphere. All spectroscopic measurements were conducted for thin films on commercially available quartz substrates. Prior to use, the quartz substrates were washed with toluene, acetone and isopropanol (spin coater, 3000 rpm, 30 sec.). The thin films were spin-coated (2 × 50 or 2 × 60 µL) onto the quartz substrates from solutions of 5 mg/mL or 10 mg/mL in $CHCl_3$ or CH_2Cl_2 (2000 rpm, 60 sec).

Fluorescence Spectroscopy in Solution: The fluorescence spectra were recorded using a FLS 980 fluorescence spectrometer from Edinburgh Instruments equipped with a double monochromator for emission and excitation. The spectra were corrected against photomultiplier and lamp intensity. The fluorescence quantum yields were determined by the optical dilution method (OD ≤ 0.05)[1] as the average value of four to six different excitation wavelengths with perylene (Φ_{fl} = 0.94 in cyclohexane)[2] as a standard.

Fluorescence Spectroscopy in the Solid State: Absolute fluorescence quantum yields of powders were determined on a Hamamatsu Absolute PL Quantum Yield Measurement System CC9920-02. The system is composed of a 150 W CW Xenon lamp as the excitation source, a monochromator (250−700 nm, full width at half-maximum (FWHM) 10 nm), an integrating sphere, and a multichannel spectrometer capable of simultaneously measuring multiple wavelengths between 300 and 950 nm.

Circular Dichroism (CD) Spectroscopy: CD spectra were measured with a Jasco J-810 spectropolarimeter equipped with a Jasco CDF-426S Peltier temperature controller.

Electrochemistry: Cyclic voltammetry (CV), differential pulse voltammetry (DPV) and square-wave voltammetry (SW) measurements were performed on a standard, commercial electrochemical analyzer (EC epsilon; BAS Instruments, UK) in a three electrode single-compartment cell under an argon atmosphere. The supporting electrolyte NBu_4PF_6 was synthesized according to the literature,[3] recrystallized from ethanol/water, and dried in a high vacuum. The measurements were carried out in CH_2Cl_2/0.1 M NBu_4PF_6 under the exclusion of air and moisture at a concentration of c ~ 2.5-2.8 × 10^{-4} M with the ferrocenium/ferrocene redox couple as an internal standard for the calibration of the potential. Working electrode: glassy carbon (Ø 1 mm); reference electrode: Ag/AgCl; auxiliary electrode: Pt wire. The internal resistance was compensated by 50%.

2. Synthesis and Characterization of Organoboron Helicenes

The synthesis of the **BIQ-Cl**, **PHE-Bpin** and **BEN-Bpin** building blocks are shown in Scheme S1.

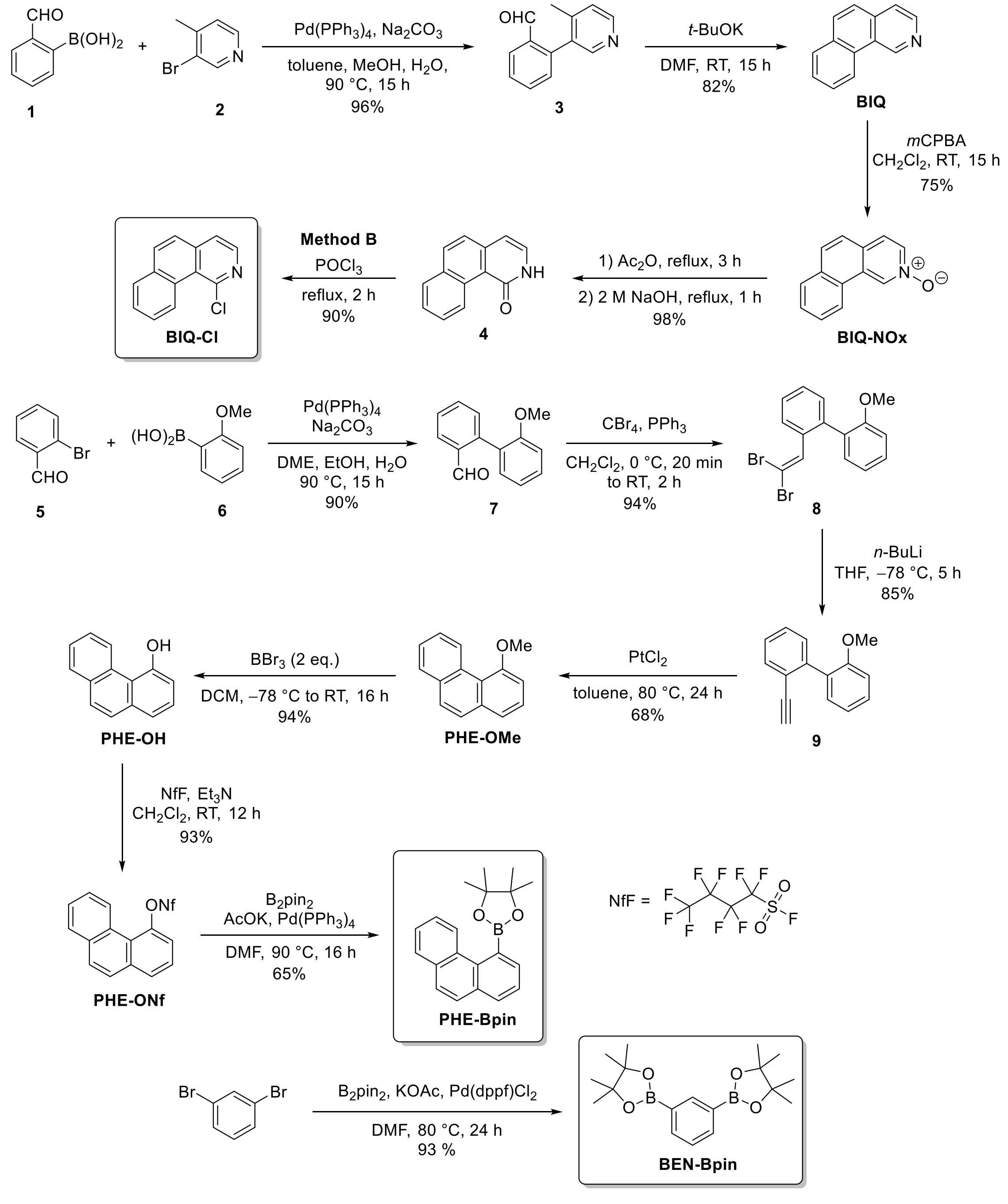

Scheme S1. Synthesis of building blocks **BIQ-Cl**, **PHE-Bpin** and **BEN-Bpin**.

Synthesis of 2-(4-methylpyridin-3-yl)benzaldehyde (3).

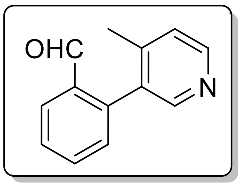

The compound was prepared according to the reported procedure.[4] 3-Bromo-4-methylpyridine **1** (3.00 g, 17.4 mmol, 1.00 eq.) was added to a degassed solution of $Pd(PPh_3)_4$ (1.01 g, 0.87 mmol, 0.05 eq.) in toluene (70 mL) under a nitrogen atmosphere. Subsequently, a degassed solution 2-formylphenylboronic acid **2** (2.87 g, 19.2 mmol, 1.10 eq.) in MeOH (35 mL) and a degassed solution of Na_2CO_3 (3.70 g, 34.8 mmol, 2.00 eq.) in water (35 mL) were added to the mixture. After heating at 90 °C for 15 h, the reaction mixture was cooled to room temperature and extracted with EtOAc. The combined organic layers were dried over $MgSO_4$, filtered and the solvent was removed under reduced pressure. The crude product was purified

via column chromatography (silica, hexane/EtOAc 85:15) to obtain compound **3** (3.30 g, 96%) as a light yellow solid. **^{1}H NMR** (400 MHz, CD_2Cl_2): δ = 9.76 (s, 1H, CHO), 8.50 (d, J = 5.0 Hz, 1H, ArH^{Py}), 8.37 (s, 1H, ArH^{Py}), 8.02 (ddd, J = 7.8, 1.4 Hz, 0.4 Hz, 1H, ArH^{Ph}), 7.70 (td, J = 7.5, 1.5 Hz, 1H, ArH^{Ph}), 7.61–7.56 (m, 1H, ArH^{Ph}), 7.32 (ddd, J = 7.6, 1.2, 0.5 Hz, $1H^{Ph}$), 7.25 (d, J = 5.0 Hz, 1H, ArH^{Py}), 2.11 ppm (s, 3H, CH_3). Analytical data are in accordance with the literature.[4]

Synthesis of benzo[*h*]isoquinoline (BIQ).

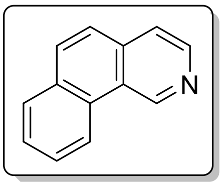

The compound was prepared according to the reported procedure.[4] Under a nitrogen atmosphere, a solution of *t*-BuOK (2.28 g, 20.3 mmol, 2.00 eq.) in dry DMF (20 mL) was added dropwise to a solution of aldehyde **3** (2.00 g, 10.1 mmol, 1.00 eq.) in dry DMF (15 mL) and the mixture was stirred at room temperature for 15 h. Subsequently, the reaction was quenched by addition of water. The phases were separated and the aqueous layer was extracted with CH_2Cl_2. The combined organic layers were dried over $MgSO_4$, concentrated under reduced pressure and the crude product was purified via column chromatography (silica, hexane/EtOAc 85:15) to obtain compound **BIQ** (1.50 g, 82%) as a colorless solid. **^{1}H NMR** (400 MHz, $CDCl_3$): δ 10.06 (s, 1H, ArH^{Py}), 8.81 (dd, J = 8.2, 0.6 Hz, 1H, ArH), 8.72 (d, J = 5.4 Hz, 1H, ArH^{Py}), 7.96–7.93 (m, 2H, ArH), 7.75 (ddd, J = 8.4, 7.1, 1.5, 1H, ArH), 7.72–7.65 ppm (m, 3H, ArH). Analytical data are in accordance with the literature.[4]

Synthesis of benzo[*h*]isoquinolin-*N*-oxide (BIQ-NOx).

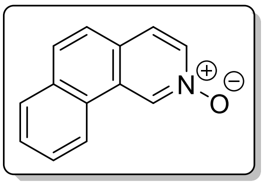

Compound **BIQ** (3.44 g, 19. mmol, 1.00 eq.) was dissolved in CH_2Cl_2 (40 mL) and the mixture was cooled to 0 °C. *m*-Chloroperoxybenzoic acid (6.63 g, 38.4 mmol, 2.00 eq.) was added portionwise. The mixture was allowed to warm to room temperature and it was stirred for 15 h. To dissolve the precipitated solid, MeOH (100 mL) was added followed by saturated aqueous $NaHCO_3$ (40 mL). The mixture was extracted with CH_2Cl_2. The combined organic layers were dried over Na_2SO_4, filtered, and the solvent was removed *in vacuo*. The crude product was purified via column chromatography (silica, EtOAc, then EtOAc/MeOH 10:1) to obtain **BIQ-NOx** (2.82 g, 75%) as a beige solid. **m.p.** 177.2 – 183.2 °C (decomposition). **^{1}H NMR** (400 MHz, $CDCl_3$): δ = 9.46 (s, 1H, ArH), 8.46–8.40 (m, 1H, ArH), 8.31 (dd, J = 6.9, 1.7 Hz, 1H), 7.94–7.91 (m, 1H, ArH), 7.85 (d, J = 8.8 Hz, 1H), 7.77–7.68 (m, 3H, ArH), 7.64 ppm (d, J = 8.8 Hz, 1H). **^{13}C NMR** (101 MHz, $CDCl_3$): δ = 136.9, 133.7, 132.8, 130.4, 129.1, 128.7, 128.4, 128.2, 127.4, 126.8, 124.5, 123.9, 122.2 ppm. **HRMS** (APCI) *m/z* calcd for $C_{13}H_{10}NO$ $[M+H]^+$ 196.0757, found 196.0755.

Synthesis of benzo[*h*]isoquinolin-1-one (4).

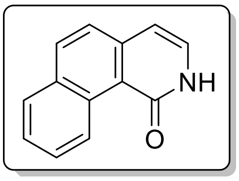

Compound **BIQ-NOx** (7.20 g, 36.9 mmol, 1.00 eq.) was dissolved in Ac_2O (122 mL) and the mixture was heated to reflux for 3 h. Subsequently, Ac_2O was removed under reduced pressure and 2 M NaOH (250 mL) was added. The mixture was heated under reflux for additional 1 h, allowed to cool to room temperature and a 5% aqueous solution of acetic acid was added until pH=6 was reached. The mixture was extracted with CH_2Cl_2, the combined organic layers were dried over $MgSO_4$, filtered, and the solvent was removed *in vacuo*. The residue was recrystallized from MeOH to obtain **4** (7.0 g, 98%) as brown crystals. **m.p.** 212.5 – 213.5 °C (MeOH). **^{1}H NMR** (400 MHz, DMSO-d_6): δ = 11.69 (br s, 1H, NH), 10.13 (dm, J = 8.6 Hz, 1H, ArH), 8.14 (d, J = 8.6 Hz, 1H, ArH), 8.01 (dd, J = 7.9 Hz, 1.4 Hz, 1H, ArH), 7.72–7.68 (m, 2H, ArH), 7.62 (ddd, J = 8.1, 6.9, 1.3, 1H, ArH), 7.45 (d, J = 6.6 Hz, 1H, ArH), 6.74 ppm (d, J = 6.8 Hz, 1H, ArH). **^{1}H NMR** (400 MHz, $CDCl_3$) δ = 11.66 (br s, 1H, NH), 10.23 (d, J = 8.5 Hz, 1H, ArH), 8.05 (d, J = 8.6 Hz, 1H, ArH), 7.93 (dd, J = 8.0, 1.4 Hz, 1H), 7.78 (ddd, J = 8.6, 6.9, 1.5 Hz, 1H), 7.64 (ddd, J = 8.1, 6.9, 1.2 Hz, 1H), 7.58 (d, J = 8.6 Hz, 1H, ArH), 7.45 (d, J = 6.8 Hz, 1H, ArH), 6.74 ppm (d, J = 6.9 Hz, 1H, ArH).**^{13}C NMR** (101 MHz, DMSO-d_6): δ = 162.8 (CO), 139.9, 133.4, 131.7, 131.5, 130.8, 128.4, 127.9, 126.4, 126.0, 125.2, 118.8, 105.7 ppm. **HRMS** (APCI) *m/z* calcd for $C_{13}H_{10}NO$ $[M+H]^+$ 196.0757, found 196.0755. **Elemental analysis** calcd (%) for $C_{13}H_9NO$: C, 79.98; H, 4.65; N, 7.17, found: C, 79.79; H, 4.63; N, 7.15. Analytical data are in accordance with the literature.[5]

Synthesis of 1-chlorobenzo[*h*]isoquinoline (BIQ-Cl).

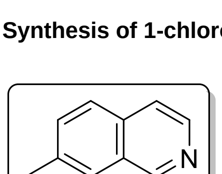

Method A: The compound was prepared according to the reported procedure.[6] *m*-Chloroperoxybenzoic acid (*m*-CPBA) (2.17 g, 12.5 mmol, 1.50 eq.) was added slowly to a solution of benzo[*h*]isoquinoline **BIQ** (1.5 g, 8.37 mmol, 1.00 eq.) in dry CH_2Cl_2 (15 mL). The reaction mixture was stirred at room temperature for 4 h, quenched with saturated aqueous Na_2CO_3 solution and extracted with CH_2Cl_2. The combined organic layers were dried over Na_2SO_4, filtered and the solvent was removed under reduced pressure. The crude benzo[*h*]isoquinoline *N*-oxide was directly used without any further purification. $POCl_3$ (2.58 mL, 27.6 mmol, 3.30 eq.) was added to a solution to crude benzo[*h*]isoquinoline *N*-oxide in dry chloroform (25 mL) and the mixture was refluxed for 3 h. Subsequently, the reaction mixture was cooled to room temperature and poured into ice water. Concentrated aqueous ammonia was added dropwise until the solution was basic. The layers were separated and the aqueous layer was extracted with CH_2Cl_2. The combined organic layers were dried over Na_2SO_4, filtered and the solvent was removed under reduced pressure. The crude product was recrystallized (chloroform/hexane) to obtain compound **BIQ-Cl** (1.50 g, 83%) as yellow crystals. **^{1}H NMR** (400 MHz, $CDCl_3$): δ =9.93–9.89 (dm, 1H, ArH), 8.45 (d, J = 5.1 Hz, 1H, ArH), 7.99–7.95 (m, 2H, ArH), 7.79–7.68 ppm (m, 4H, ArH).

Method B: A solution of **4** (50.0 mg, 0.35 mmol, 1.00 eq.) in $POCl_3$ (1.00 mL, 1.07 mmol, 41.0 eq.) was refluxed for 2 h under a nitrogen atmosphere. Then $POCl_3$ was evaporated and cold, saturated aqueous Na_2CO_3 (2 mL) was added to the residue. The mixture was extracted with CH_2Cl_2. The combined organic layers were dried over $MgSO_4$, filtered and concentrated under reduced pressure. The residue was filtered through a short plug of silica (EtOAc) and the solvent was removed *in vacuo* to obtain **BIQ-Cl** (49.0 mg, 90%) as a yellow solid. **m.p.** 96.7 – 98.9 °C. **^{1}H NMR** (400 MHz, CD_2Cl_2): δ = 9.90–9.87 (dm, 1H, ArH), 8.42 (d, *J* = 5.1 Hz, 1H, ArH), 8.04–7.95 (m, 2H, ArH), 7.82–7.69 ppm (m, 4H, ArH). **^{1}H NMR** (400 MHz, $CDCl_3$): δ = 9.93–9.88 (m, 1H, ArH), 8.45 (d, *J* = 5.1 Hz, 1H, ArH), 8.00–7.95 (m, 2H, ArH), 7.81–7.69 ppm (m, 4H, ArH). **^{13}C NMR** (101 MHz, CD_2Cl_2): δ = 149.3, 143.6, 140.8, 134.2, 133.5, 129.7, 129.0, 128.3, 127.9, 127.4, 125.7, 124.4, 122.4 ppm. **HRMS** (ESI) *m/z* calcd for $C_{13}H_9ClN$ $[M+H]^+$ 214.0418, found 214.0416. **Elemental analysis** calcd (%) for $C_{13}H_8ClN$: C, 73.08; H, 3.77; N, 6.56, found: C, 72.99; H, 4.00; N, 6.57.

Synthesis of 2'-methoxy-[1,1'-biphenyl]-2-carbaldehyde (7).

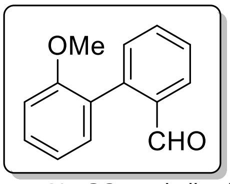

The compound was prepared according to the reported procedure.[7] 2-Bromobenzaldehyde **5** (5.00 g, 27.0 mmol, 1.00 eq.) was dissolved in degassed DME (70 mL) and $Pd(PPh_3)_4$ (937 mg, 0.81 mmol, 0.03 eq.) was added. Subsequently, a degassed solution of 2-methoxyphenylboronic acid **6** (4.93 g, 32.4 mmol, 1.20 eq.) in EtOH (15 mL) and a degassed solution of Na_2CO_3 (5.73 g, 54.0 mmol, 2.00 eq.) in water (15 mL) were added. After heating under reflux for 15 h the reaction mixture was brought to room temperature and diluted with diethyl ether. The phases were separated and the organic layer was washed with brine, dried over Na_2SO_4 and all volatiles were removed under reduced pressure. The crude product was purified via flash column chromatography (silica, hexane/EtOAc 90:10) to obtain compound **7** (5.10 g, 90%) as a white solid. **^{1}H NMR** (400 MHz, $CDCl_3$): δ = 9.79 (d, *J* = 0.8 Hz, 1H, CHO), 8.00 (dd, *J* = 7.8, 1.5, 0.5 Hz, 1H, ArH), 7.65 (td, *J* = 7.5, 1.5 Hz, 1H, ArH), 7.51–7.46 (m,1H, ArH), 7.42 (tm, *J* = 8.3, 7.5, 1.8 Hz, 1H, ArH), 7.36 (dm, *J* = 7.7, 1.3, 0.5 Hz,1H, ArH), 7.29 (dd, *J* = 7.5, 1.8, 0.2 Hz, 1H, ArH), 7.09 (td, *J* = 7.5, 1.1 Hz, 1H, ArH), 6.98 (dd, *J* = 8.3, 0.9 Hz,1H, ArH), 3.74 ppm (s, 3H, OCH_3). Analytical data are in accordance with the literature.[7]

Synthesis of 2-(2,2-dibromovinyl)-2'-methoxy-1,1'-biphenyl (8).

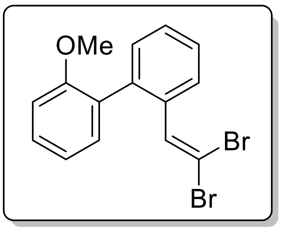

CBr_4 (19.5 g, 58.9 mmol, 2.50 eq.) was added to a solution of PPh_3 (30.9 g, 117 mmol, 5.00 eq.) in dry CH_2Cl_2 (400 mL) under a nitrogen atmosphere and the resulting mixture was stirred at 0 °C for 20 min. Subsequently, a solution of aldehyde **7** (5.00 g, 23.5 mmol, 1.00 eq.) in CH_2Cl_2 (100 mL) was added dropwise and the reaction was allowed to warm to room temperature. After stirring for 2 h the reaction was quenched with brine and extracted with CH_2Cl_2. The combined organic layers were dried over Na_2SO_4, filtered, and the solvent was removed in vacuo. The crude product was purified via column chromatography (silica, hexane/EtOAc = 95:5) to obtain compound **8** (8.20 g, 94%) as a yellow oil. **^{1}H NMR** (400 MHz, $CDCl_3$): δ **=** 7.72–7.64 (m, 1H, ArH), 7.45–7.35 (m, 3H, ArH), 7.35–7.29 (m, 1H, ArH), (ddd, *J* = 7.5, 1.8, 0.2 Hz, 1H), 7.15 (d, *J* = 0.4 Hz, 1H, H^{vinyl}), 7.04 (td, *J* = 7.4, 1.1 Hz, 1H, ArH), 6.99 (dd, *J* = 8.3, 1.0 Hz, 1H, ArH), 3.81 (s, 3H, OCH_3). **^{13}C NMR** (101 MHz, $CDCl_3$): δ **=** 156.7, 138.2, 137.5, 135.3, 131.4, 130.6, 129.5, 129.1, 128.52, 128.48, 127.2, 120.7, 111.0, 90.1, 55.6 ppm. **HRMS** (APCI): *m/z* calcd for $C_{15}H_{13}Br_2O$ $[M+H]^+$: 366.9328; found: 366.9333.

Synthesis of 2-ethynyl-2'-methoxy-1,1'-biphenyl (9).

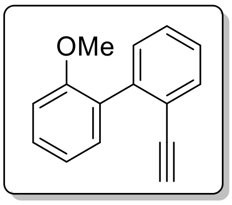

The compound was prepared according to the reported procedure.[7] Dibromide **8** (8.00 g, 21.7 mmol, 1.00 eq.) was dissolved in dry THF (120 mL) under a nitrogen atmosphere, and cooled to -78 °C. Subsequently, *n*-BuLi (1.6 M in THF, 34.0 mL, 54.3 mmol, 2.50 eq.) was added and the reaction mixture was stirred at -78 °C for 5 h. Afterwards, the reaction was allowed to warm to room temperature, quenched with water and extracted with diethyl ether. The combined organic layers were dried over Na_2SO_4, filtered, and all volatiles were removed under reduced pressure. The crude product was purified via flash chromatography (silica, hexane/EtOAc 98:2) to obtain alkyne **9** (3.80 g, 85%) as a colorless oil. **^{1}H NMR** (400 MHz, $CDCl_3$): δ = 7.60 (dd, *J* = 7.6, 0.8 Hz, 1H, ArH), 7.43–7.27 (m, 5H, ArH), 7.05–6.97 (m, 2H, ArH), 3.79 (s, 3H, OCH_3), 2.93 ppm (s, 1H, CCH). Analytical data are in accordance with the literature.[7]

Synthesis of 4-methoxyphenanthrene (PHE-OMe).

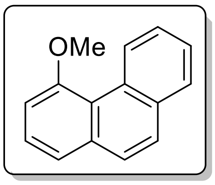

The compound was prepared according to the reported procedure.[7] **9** (3.50 g, 16.8 mmol, 1.00 eq.) was dissolved in dry toluene (60 mL) under an argon atmosphere and $PtCl_2$ (224 mg, 840 µmol, 0.05 eq.) was added. The reaction was heated to 80 °C and it was stirred for 24 h at this temperature. Afterwards, the reaction was allowed to get room temperature and subsequently the solvent was removed under reduced pressure. The crude product was purified via column chromatography (silica, hexane/EtOAc = 90:10) to obtain compound **PHE-OMe** (2.38 g, 68%) as a colorless solid. **^{1}H NMR** (400 MHz, $CDCl_3$): δ = 9.67 (dm, *J* = 8.5 Hz, 1H, ArH), 7.88 (dd, *J* = 7.7, 1.7 Hz, 1H, ArH), 7.75 (d, *J* = 8.8 Hz, 1H, ArH), 7.71 (d, *J* =8.8 Hz, 1H, ArH), 7.64 (ddd, *J* = 8.6, 7.0, 1.7, 1H, ArH), 7.58 (ddd, *J* = 7.7, 7.0, 1.3, 1H, ArH), 7.55–7.52 (m, 2H, ArH), 7.17 (dd, *J* =6.2, 2.9 Hz, 1H, ArH), 4.15 ppm (s, 1H, OCH_3). Analytical data are in accordance with the literature.[7]

Synthesis of phenanthren-4-ol (PHE-OH).

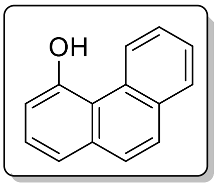

The compound was prepared according to the reported procedure.[8] Compound **PHE-OMe** (2.50 g, 12.0 mmol, 1.00 eq.) was dissolved in dry CH_2Cl_2 (75 mL) under a nitrogen atmosphere and the reaction mixture was cooled to -78 °C. Then BBr_3 (2.28 mL, 24.0 mmol, 6.01 g, 2.00 eq.) was added. The mixture was allowed to warm to room temperature and was stirred at this temperature for 16 h. Subsequently, the reaction was quenched by the addition of water. The phases were separated and the aqueous layer was extracted with CH_2Cl_2. The combined organic layers were washed with brine, dried over $MgSO_4$ and all volatiles were removed under reduced pressure. The crude product was purified via column chromatography (silica, hexane/CH_2Cl_2 4:1) to obtain compound **PHE-OH** (2.19 g, 94%) as a colorless solid. ^{1}**H NMR** (400 MHz, $CDCl_3$): δ = 9.66 (dd, J = 8.5, 1.2, 0.7 Hz, 1H, ArH), 7.90 (dd, J = 7.8, 1.6 Hz, 1H, ArH), 7.75 (d, J = 8.9 Hz, 1H, ArH), 7.72-7.65 (m, 2H, ArH), 7.61 (ddd, J = 7.8, 7.0, 1.3, 1H, ArH), 7.53 (dd, J = 7.9, 1.2 Hz, 1H, ArH), 7.43 (t, J = 7.7 Hz, 1H, ArH), 6.97 (dd, J = 7.6, 1.3 Hz, 1H, ArH), 5.64 ppm (d, J = 0.5 Hz, 1H, OH). Analytical data are in accordance with the literature.[8]

Synthesis of phenanthren-4-yl 1,1,2,2,3,3,4,4,4-nonafluorobutane-1-sulfonate (PHE-ONf).

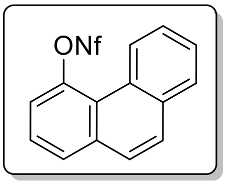

Compound **PHE-OH** (1.31 g, 6.73 mmol, 1.00 eq.) was dissolved in CH_2Cl_2 (60 mL) and and NEt_3 (2.00 ml, 14.3 mmol, 1.45 g, 2.13 eq.) was added, followed by perfluorobutane-1-sulfonyl fluoride (1.46 ml, 8.13 mmol, 2.46 g, 1.21 eq.). The reaction mixture was stirred at room temperature under exclusion of light. After 12 h dist. H_2O was added (50 mL). The phases were separated and the aqueous layer was extracted with CH_2Cl_2 (3 x 50 mL). The combined organic layers were washed with H_2O (3 x 50 mL), dried over $MgSO_4$, filtered and evaporated. The crude product was purified via column chromatography (silica, hexane/EtOAc 4:1) to obtain compound **PHE-ONf** (3.00 g, 93%) as a colorless oil. ^{1}**H NMR** (400 MHz, CD_2Cl_2): δ = 9.20–9.15 (m, 1H, ArH), 8.00–7.93 (m, 2H, ArH), 7.85 (d, J = 8.8 Hz, 1H, ArH), 7.78 (d, J = 8.9 Hz, 1H, ArH), 7.75–7.68 (m, 2H, ArH), 7.67–7.61 ppm (m, 2H, ArH). ^{1}**H NMR** (400 MHz, $CDCl_3$): δ = 9.19 (dd, J = 8.4, 1.1 Hz, 1H, ArH), 7.96–7.90 (m, 2H, ArH), 7.82 (d, J = 8.8 Hz, 1H, ArH), 7.77–7.58 ppm (m, 5H, ArH). ^{13}**C NMR** (101 MHz, CD_2Cl_2): δ = 147.8, 135.8, 133.9, 129.9 (CH), 129.6 (CH), 129.5 (CH), 128.3 (CH), 127.9, 127.73 (CH), 127.70 (CH), 126.88 (CH), 126.85 (CH), 123.7, 121.2 (t, J = 1.8 Hz, 1C, CH), 119.4-106.2 ppm (m, 4C. CF). Analytical data are in accordance with the literature.[8]

Synthesis of 4,4,5,5-tetramethyl-2-(phenanthren-4-yl)-1,3,2-dioxaborolane (PHE-Bpin).

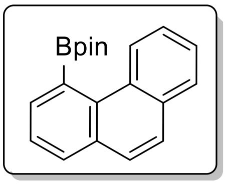

Compound **PHE-ONf** (3.00 g, 7.28 mmol, 1.00 eq.), bis(pinacolato)diborane (2.77 g, 10.9 mmol, 1.50 eq.) and KOAc (4.29 g, 43.7 mmol, 6.00 eq.) were dissolved in anhydrous DMF (100 mL) under an inert atmosphere, and the mixture was degassed for 30 min. Subsequently, $Pd(PPh_3)_4$ (841 mg, 728 µmol, 0.10 eq.) was added and the reaction was heated to 90 °C. The reaction mixture was stirred at this temperature for 16 h and then allowed to cool to room temperature. The mixture was filtered through a plug of Celite and the filtrate was washed with ice water and brine. The organic layer was dried over $MgSO_4$, filtered and the solvent was removed under reduced pressure. The crude product was purified via column chromatography (silica, hexane/EtOAc, 95:5) to obtain **PHE-Bpin** (1.44 g, 65%) as a colorless solid. ^{1}**H NMR** (400 MHz, $CDCl_3$): δ = 8.57–8.53 (m, 1H, ArH), 7.92 (dd, J = 7.9, 1.4 Hz, 1H, ArH), 7.89–7.87 (m, 1H, ArH), 7.84 (dd, J = 7.0, 1.4 Hz, 1H, ArH), 7.74 (d, J = 9.0 Hz,1H ArH), 7.71 (d, J = 8.8 Hz,1H ArH), 7.61–7.54 (m, 3H, ArH), 1.50 ppm (s, 12H, CH_3). ^{1}**H NMR** (400 MHz, CD_2Cl_2): δ = 8.54–8.51 (m, 1H, ArH), 7.95 (dd, J = 7.9, 1.5 Hz, 1H, ArH), 7.93–7.90 (m, 1H, ArH), 7.82 (dd, J = 7.0, 1.4 Hz, 1H, ArH), 7.77 (d, J = 9.0 Hz, 1H, ArH), 7.74 (d, J = 8.8 Hz, 1H, ArH), 7.65–7.56 (m, 3H, ArH), 1.48 ppm (s, 12H, CH_3). ^{13}**C NMR** (101 MHz, CD_2Cl_2) δ 134.1, 133.4, 133.3, 132.8, 131.3, 130.9, 128.8, 128.0, 127.4, 127.2, 127.1, 126.3, 125.8, 84.8 (*C*CH_3), 25.10 (CCH_3). One carbon signal is not visible due to the quadrupolar relaxation ^{11}**B NMR** (128 MHz, CD_2Cl_2): δ = 32.8 ppm. **HRMS** (ESI) *m/z* calcd for $C_{20}H_{21}BNaO_2$ $[M+Na]^+$ 327.1527, found: 327.1536.

Synthesis of 1-(phenanthren-4-yl)benzo[*h*]isoquinoline (BA).

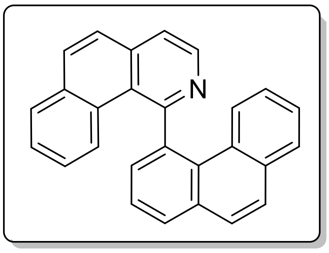

Method A: 1-Chlorobenzo[*h*]isoquinoline **BIQ-Cl** (125 mg, 585 µmol, 1.00 eq.) was dissolved in DME (4 mL) under an inert atmosphere and a solution of boronic acid ester **PHE-Bpin** (178 mg, 585 µmol, 1.00 eq.) in MeOH (1.2 mL) was added followed by a solution of Na_2CO_3 (186 mg, 1.76 mmol. 3.00 eq.) in water (1.4 mL). The mixture was degassed and subsequently $Pd(PPh_3)_4$ (20.3 mg, 18.0 µmol, 0.03 eq.) was added. The reaction mixture was heated up to 85 °C for 16 h. Afterwards the reaction mixture was brought to room temperature, diluted with water and extracted with EtOAc. The combined organic layers were washed with water and brine, dried over Na_2SO_4, filtered and the solvent was removed under reduced pressure. The crude product was purified by flash column chromatography (silica, hexane/EtOAc 8:2) to obtain desired compound **BA** (110 mg, 53%) as a white solid. ^{1}**H NMR** (400 MHz, $CDCl_3$): δ = 8.77 (d, J = 5.3 Hz, 1H, ArH), 8.14-8.01 (m, 2H, ArH), 7.91-7.82 (m, 7H, ArH), 7.63 (t, J = 7.6, 1H, ArH), 7.49-7.42 (m, 2H, ArH), 7.37 (ddd, J = 8.0, 7.0, 1.0 Hz, 1H, ArH), 7.32 (dm, J = 7.2 Hz, 1H, ArH), 7.05 (ddd, J = 8.6, 7.0, 1.4 Hz, 1H, ArH), 6.95 ppm (ddd, J = 8.5, 7.0, 1.5 Hz, 1H, ArH). Analytical data are in accordance with the literature.[6]

Method B: 1-Chlorobenzo[*h*]isoquinoline **BIQ-Cl** (100 mg, 468 µmol, 1.00 eq.) was dissolved in DME (2 mL) under an inert atmosphere and a solution of boronic acid ester **PHE-Bpin** (156 mg, 515 µmol, 1.10 eq.) in MeOH (1 mL) was added followed by a solution of Cs_2CO_3 (305 mg, 936 µmol, 2.00 eq.) in water (1 mL). The mixture was degassed and subsequently $Pd(PPh_3)_4$ (27.0 mg, 23.4 µmol, 0,05 eq.) was added. The reaction mixture was heated up to 80 °C. After 15 h the reaction was brought to room temperature, diluted with water and the mixture was extracted with CH_2Cl_2. The combined organic layers were washed with water and brine, dried over $MgSO_4$, filtered and the volatiles were removed under reduced pressure. The crude product was purified via flash column chromatography (silica, hexane/EtOAc 3:2) to obtain compound **BA** (87.0 mg, 52%) as a colorless solid. **^{1}H NMR** (400 MHz, $CDCl_3$): δ = 8.75 (d, *J* = 5.2 Hz, 1H, ArH), 8.05 (dd, *J* = 7.9, 1.4 Hz, 1H, ArH), 8.02 (d, *J* = 8.8 Hz, 1H, ArH), 7.93–7.81 (m, 7H, ArH), 7.61 (dd, *J* = 7.8, 7.3, 1H, ArH), 7.54 (dm, *J* = 8.6 Hz, 1H, ArH), 7.43 (ddd, *J* = 8.0, 7.0, 1.1 Hz, 1H, ArH), 7.37 (ddd, *J* = 8.0, 7.0, 1.1 Hz, 1H, ArH), 7.28 (dd, *J* = 7.3, 1.4 Hz, 1H, ArH), 7.04 (ddd, *J* = 8.6, 7.0, 1.5 Hz, 1H, ArH), 6.96 ppm (ddd, *J* = 8.6, 7.0, 1.5 Hz, 1H, ArH). Analytical data are in accordance with the literature.[6]

Synthesis of H1-Br$_2$.

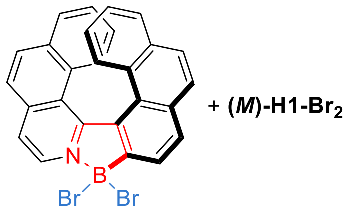

(*P*)-H1-Br$_2$

Biaryl **BA** (70.0 mg, 197 µmol, 1.00 eq.) was dissolved in CH_2Cl_2 (2 mL) and *i*-Pr_2NEt (351 µL, 197 µmol) was added under an inert atmosphere. The mixture was cooled to 0 °C and BBr_3 (56.1 µL, 591 µmol, 148 mg, 3.00 eq.) was added dropwise. The reaction mixture was brought to room temperature and it was stirred for 24 h at this temperature. Subsequently, the reaction was quenched with saturated aqueous K_2CO_3 solution and the mixture was diluted with CH_2Cl_2 and water. The phases were separated and the aqueous phase was extracted with CH_2Cl_2. The combined organic layers were washed with water and brine, dried over $MgSO_4$, filtered and the volatiles were removed under reduced pressure. The crude product was used in the next step without any further purification. **HRMS** (ESI) *m/z* calcd for $C_{27}H_{16}BBr_2NNa$ $[M+Na]^+$ 545.9635, found: 545.9621.

Synthesis of H1-Me$_2$.

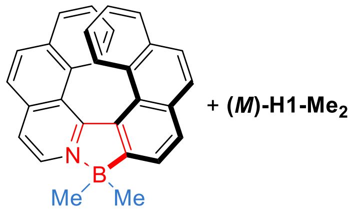

(*P*)-H1-Me$_2$

Compound **H1-Br$_2$** (50.0 mg, 952 µmol, 1.00 eq.) was dissolved in a mixture of CH_2Cl_2 (1 mL) and toluene (1 mL) and $AlMe_3$ (2 M solution in hexane, 105 µL, 210 µmol, 2.20 eq.) was added dropwise under an inert atmosphere. The reaction was stirred for 4 h and quenched with water. The phases were separated and the aqueous layer was extracted with CH_2Cl_2. The combined organic layers were washed with water and brine, dried over $MgSO_4$, filtered and the solvent was removed under reduced pressure. The crude product was purified via flash chromatography (silica, CH_2Cl_2/hexane 95:5) to obtain compound **H1-Me$_2$** (20.0 mg, 53%) as a yellow solid. **m.p.** 259-262 °C. **^{1}H NMR** (400 MHz, CD_2Cl_2): δ = 8.54 (d, *J* = 5.8 Hz, 1H, ArH), 8.00–7.97 (m, 2H, ArH), 7.94 (d, *J* = 7.5 Hz, 1H, ArH), 7.85–7.81 (m, 2H, ArH), 7.79 (d, *J* = 8.7 Hz, 1H, ArH), 7.66 (d, *J* = 8.6 Hz, 1H, ArH), 7.64–7.57 (m, 2H, ArH), 7.55 (dm, *J* = 7.9 Hz, 1H, ArH), 7.28 (dm, *J* = 8.5 Hz, 1H, ArH), 7.15 (ddd, *J* = 8.0, 7.0, 1.2 Hz, 1H, ArH), 7.01 (ddd, *J* = 8.0, 6.9, 1.2 Hz, 1H, ArH), 6.43 (ddd, *J* = 8.4, 7.0, 1.4 Hz, 1H, ArH), 6.30 (ddd, *J* = 8.4, 6.9, 1.4 Hz, 1H, ArH), 0.26 (s, 3H, CH_3), 0.19 (s, 3H, CH_3) ppm. **^{13}C NMR** (101 MHz, CD_2Cl_2): δ = 157.2, 139.1, 136.1, 134.1, 132.7, 132.54, 132.45, 131.71, 131.69, 130.4, 129.9, 129.3, 128.3, 127.7, 127.48, 127.44, 127.0, 126.7, 125.9, 125.82, 125.79, 124.7, 124.5, 123.7, 120.2 ppm. Signals corresponding to the C atoms bound to the B atom are not visible. **^{11}B NMR** (128 MHz, CD_2Cl_2): δ = 0.4 ppm. **HRMS** (APCI) *m/z* calcd for $C_{29}H_{23}BN$ $[M+H]^+$ 396.1918, found 396.1908.

Synthesis of H1-Et$_2$.

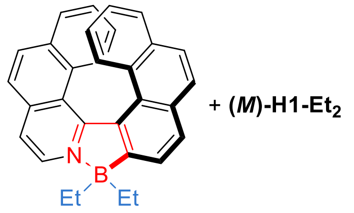

(*P*)-H1-Et$_2$

Method A: Compound **H1-Br$_2$** (30.0 mg, 57.1 µmol, 1.00 eq.) was dissolved in a mixture of CH_2Cl_2 (0.5 mL) and toluene (0.5 mL) and $AlEt_3$ (1 M solution in *n*-hexane, 126 µL, 126 µmol, 2.20 eq.) was added dropwise under an inert atmosphere. The reaction was stirred for 2 h and subsequently quenched with water. The phases were separated and the aqueous layer was extracted with CH_2Cl_2. The combined organic layers were washed with water and brine, dried over $MgSO_4$, filtered and the solvent was removed under reduced pressure. The crude product was purified via flash chromatography (silica, CH_2Cl_2/hexane 10:1) to obtain compound **H1-Et$_2$** (13 mg, 54%) as a light yellow solid. **m.p.** 189 – 192 °C. **^{1}H NMR** (400 MHz, CD_2Cl_2): δ = 8.44 (d, *J* = 5.9 Hz, 1H, ArH), 8.01–7.97 (m, 2H, ArH), 7.93 (d, *J* = 7.5 Hz, 1H, ArH), 7.86–7.81 (m, 2H, ArH), 7.80 (d, *J* = 8.7 Hz, 1H, ArH), 7.65 (d, *J* = 8.6 Hz, 1H, ArH), 7.63–7.58 (m, 2H, ArH), 7.54 (dm, *J* = 7.9 Hz, 1H, ArH), 7.29 (dm, *J* = 8.4 Hz, 1H, ArH), 7.15 (ddd, *J* = 8.0, 7.0, 1.1, 1H, ArH), 7.00 (ddd, *J* = 8.0, 6.9, 1.2, 1H, ArH), 6.44 (ddd, *J* = 8.4, 7.1, 1.3, 1H, ArH), 6.31 (ddd, *J* = 8.4, 6.9, 1.4, 1H, ArH), 1.13–0.95 (m, 2H, CH_2), 0.94–0.83 (m, 1H, CH_2), 0.78–0.65 (m, 1H, CH_2), 0.50 (t, *J* = 7.7 Hz, 3H, CH_3), 0.28 ppm (t, *J* = 7.6 Hz, 3H, CH_3). **^{13}C NMR** (101 MHz, CD_2Cl_2) δ 158.0, 138.9, 135.8, 134.2, 134.1, 132.51, 132.48, 131.6, 131.3, 130.4, 130.0, 129.1, 128.2, 127.77, 127.70, 127.47, 126.9, 126.7, 125.8, 125.8, 125.7, 124.6, 124.1, 123.8, 120.1, 10.3, 10.1. Signals corresponding to the C atoms bound to the

B atom are not visible. **^{11}B NMR** (128 MHz, CD_2Cl_2): *δ* = 3.0 ppm. **HRMS** (APCI) *m*/*z* calcd for $C_{31}H_{27}BN$ [M+H]$^+$ 424.2231, found: 424.2221.

Method B: Compound **H1-Br₂** (30 mg, 57.1 µmol, 1.00 eq.) was dissolved in toluene (2.5 mL) and Et_2Zn (1.1 M solution in toluene, 0.12 mL, 120 µmol, 2.1 eq.) was added slowly under an inert atmosphere. The reaction mixture was stirred at 70 °C for 15 h and afterwards it was quenched with water. The phases were separated and extracted with CH_2Cl_2, washed with brine, dried over $MgSO_4$, filtered and the solvent was removed under reduced pressure. The residue was purified by flash column chromatography (silica, hexane/EtOAc 95:5) to obtain compound **H1-Et₂** (11 mg, 45%) as a light yellow solid. ^{1}H NMR (400 MHz, $CDCl_3$): δ **=** 8.42 (d, *J* = 5.9 Hz, 1H, ArH), 7.94-7.97 (m, 3H, ArH), 7.82 (d, *J* = 8.6 Hz, 1H, ArH), 7.77-7.74 (m, 2H, ArH), 7.65-7.61 (m, 2H, ArH), 7.58 (dm, *J* = 7.8 Hz, 1H, ArH), 7.53 (dm, *J* = 7.9 Hz, 1H, ArH), 7.28 (dm, *J* = 8.5 Hz, 1H, ArH), 7.14 (ddd, *J* = 8.0, 7.0, 1.1 Hz, 1H, ArH), 7.00 (ddd, *J* = 8.0, 6.9, 1.2 Hz, 1H, ArH), 6.45 (ddd, *J* = 8.4, 7.0, 1.4 Hz, 1H, ArH), 6.31 (ddd, *J* = 8.4, 6.9, 1.4 Hz, 1H, ArH), 1.12-0.97 (m, 2H, CH_2), 0.93-0.84 (m, 1H, CH_2), 0.76-0.67 (m, 1H, CH_2), 0.52 (t, *J* = 7.7 Hz, 3H, CH_3), 0.31 ppm (t, *J* = 7.6 Hz, 3H, CH_3). **HRMS** (APCI): *m*/*z* calcd for $C_{31}H_{27}BN$ [M+H]$^+$ 424.2231, found: 424.2226.

Synthesis of H1-Ph₂.

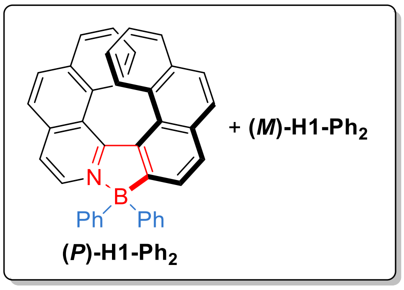

Compound **H1-Br₂** (25.0 mg, 47.6 µmol, 1.00 eq.) was dissolved in a mixture of CH_2Cl_2 (1.5 mL) and toluene (1.5 mL) and $AlPh_3$ (1 M solution in *n*-hexane, 200 µL, 200 µmol, 4.20 eq.) was added dropwise under an inert atmosphere. After the reaction was stirred at 90 °C for 4 h it was brought to room temperature and quenched with water. The phases were separated and the aqueous layer was extracted with CH_2Cl_2. The combined organic layers were washed with water and brine, dried over $MgSO_4$, filtered and the solvent was removed under reduced pressure. The crude product was purified via flash chromatography (silica, CH_2Cl_2/hexane 9:1) to obtain compound **H1-Ph₂** (12 mg, 49%) as a yellow solid. **m.p.** 281 – 285°C. **^{1}H NMR** (400 MHz, CD_2Cl_2): *δ* = 8.53 (d, *J* = 5.9 Hz, 1H, ArH), 8.02 (d, *J* = 8.7 Hz, 1H, ArH), 8.00 (d, *J* = 7.7 Hz, 1H, ArH), 7.96 (d, *J* = 7.6 Hz, 1H, ArH), 7.82 (d, *J* = 8.7 Hz, 1H, ArH), 7.80 (d, *J* = 5.8 Hz, 1H, ArH) overlapping with 7.79 (d, *J* = 8.7 Hz, 1H, ArH), 7.70–7.65 (m, 2H, ArH), 7.64 (dm, *J* = 7.9 Hz, 1H, ArH), 7.56 (dm, *J* = 7.9 Hz, 1H, ArH), 7.42–7.38 (m, 2H, ArH), 7.37–7.30 (m, 3H, ArH), 7.25–7.11 (m, 7H, ArH), 7.04 (ddd, *J* = 8.0, 7.0, 1.2 Hz, 1H, ArH), 6.48 (ddd, J = 8.4, 7.1, 1.4 Hz, 1H, ArH), 6.35 ppm (ddd, *J* = 8.4, 7.0, 1.4 Hz, 1H, ArH). **^{13}C NMR** (101 MHz, CD_2Cl_2): δ = 159.0, 139.8, 137.6, 135.0, 134.0, 133.9, 133.8, 132.7, 132.6, 132.1, 130.4, 129.9, 129.4, 128.6, 128.4, 128.05, 128.03, 127.9, 127.63, 127.61, 127.01, 126.97, 126.4, 126.2, 126.1, 125.9, 124.58, 124.56, 123.9, 120.7 ppm. **^{11}B NMR** (128 MHz, CD_2Cl_2): *δ* = 3.3 ppm. **HRMS** (APCI) *m*/*z* calcd for $C_{39}H_{27}BN$ [M+H]$^+$ 520.2231, found: 520.2220.

Synthesis of 1,3-bis(benzo[*h*]isoquinolin-1-yl)benzene (TA).

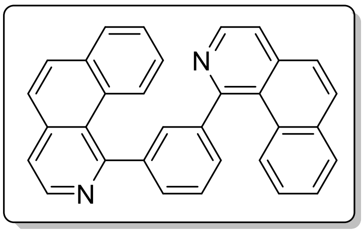

Compound **BIQ-Cl** (203 mg, 954 µmol, 2.10 eq.) was dissolved in DME (3 mL) under an inert atmosphere and a solution of boronic acid ester **BEN-Bpin**[9] (150 mg, 455 µmol, 1.00 eq.) in MeOH (1.5 mL) was added followed by a solution of Na_2CO_3 (96.3 mg, 909 µmol, 2.00 eq.) in water (1.5 mL). The mixture was degassed and subsequently, $Pd(PPh_3)_4$ (8.60 mg, 13.6 µmol, 0.03 eq.) was added. The reaction mixture was heated up to 80 °C. After 15 h the reaction was brought to room temperature, diluted with water and the mixture was extracted with CH_2Cl_2. The combined organic layers were washed with water and brine, dried over $MgSO_4$, filtered and the volatiles were removed under reduced pressure. The crude product was purified via column chromatography (silica, hexane/EtOAc 3:2) to obtain compound **TA** (175 mg, 89%) as a colorless solid. **m.p.** 299 – 233 °C. **^{1}H NMR** (400 MHz, CD_2Cl_2): *δ* = 8.67 (d, *J* = 5.2 Hz, 2H, ArH), 8.44 (br s, 1H, ArH), 8.02–7.81 (m, 6H, ArH), 7.74 (d, *J* = 8.8 Hz, 2H, ArH), 7.68 (d, *J* = 5.2 Hz, 2H, ArH), 7.68–7.56 (m, 3H, ArH), 7.52 (t, *J* = 7.1 Hz, 2H, ArH), 7.47–7.20 ppm (m, 2H, ArH). **^{13}C NMR** (101 MHz, CD_2Cl_2): δ **=** 158.9, 145.7, 144.0, 138.7, 134.1, 132.3, 130.0, 129.9, 129.5, 129.2, 128.7, 127.4, 126.5, 125.9, 124.3, 120.9 ppm. **HRMS** (ESI) *m*/*z* calcd for $C_{32}H_{21}N_2$ [M+H]$^+$ 433.1699, found 433.1717.

Synthesis of H2-Br₄.

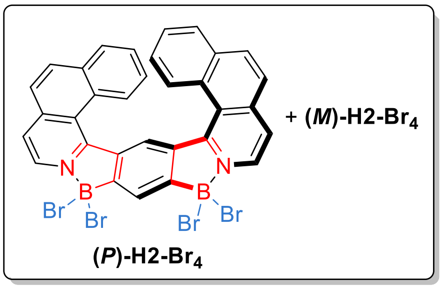

Oligoaryl **TA** (100 mg, 0.231 µmol, 1.00 eq.) was dissolved in CH_2Cl_2 (5 mL) under an inert atmosphere and *i*-Pr_2NEt (823 µL, 0.462 µmol, 2.00 eq.) was added. The mixture was cooled to 0 °C and BBr_3 (143 µL1.50 mmol, 6.50 eq.) was added dropwise. The reaction mixture was brought to room temperature and it was stirred for 48 h at this temperature. Subsequently, the reaction was quenched with saturated aqueous K_2CO_3 solution and the precipitated solid was filtered off. The mixture was diluted with CH_2Cl_2 and water. The phases were separated and the aqueous phase was extracted with CH_2Cl_2. The combined organic layers were washed with water and brine, dried over $MgSO_4$, filtered and the volatiles were removed under reduced pressure. The crude product was used in the next step without any further purification.

HRMS (APCI) *m*/*z* calcd for $C_{32}H_{18}B_2Br_4N_2$ M$^{•+}$ 767.8384, found 767.8416.

Synthesis of H2-Me$_4$.

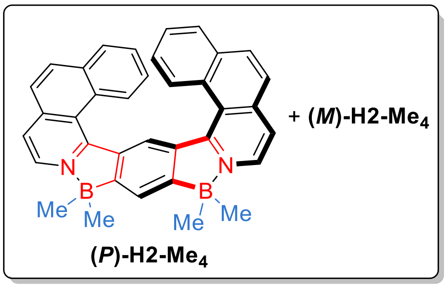

Compound **H2-Br$_4$** (64.0 mg, 82.7 µmol, 1.00 eq.) was dissolved in a mixture of CH_2Cl_2 (1.5 mL) and toluene (1.5 mL) and $AlMe_3$ (2 M solution in *n*-hexane, 174 µL, 347 µmol, 4.20 eq.) was added dropwise under an inert atmosphere. The reaction was stirred for 4 h and quenched with water. The phases were separated and the aqueous layer was extracted with CH_2Cl_2. The combined organic layers were washed with water and brine, dried over $MgSO_4$, filtered and the solvent was removed and under reduced pressure. The crude product was purified via flash chromatography (silica, CH_2Cl_2/hexane 3:2) to obtain compound **H2-Me$_4$** (29 mg, 68%) as a yellow solid. **m.p.** 246 – 249 °C (decomposition). **^{1}H NMR** (400 MHz, CD_2Cl_2): δ =10.05 (d, J = 0.8 Hz, 1H, ArH), 8.61 (d, J = 8.3 Hz, 2H, ArH), 8.43 (d, J = 5.9 Hz, 2H, ArH), 8.05 (d, J = 8.6 Hz, 2H, ArH), 8.00 (d, J = 0.8 Hz, 1H, ArH), 7.83 (dd, J = 8.0 Hz, 1.3 Hz, 2H, ArH), 7.73 (d, J = 8.7 Hz, 2H, ArH), 7.68 (d, J = 6.0 Hz, 2H, ArH), 7.24 (ddd, J = 8.1, 7.0, 1.1, 2H, ArH), 6.52 (ddd, J = 8.4, 7.1, 1.4, 2H, ArH), 0.20 ppm (s, 12H, CH_3). **^{13}C NMR** (100 MHz, CD_2Cl_2): δ = 156.3, 139.6, 136.7, 135.2, 133.9, 133.7, 129.6, 129.1, 128.6, 128.5, 127.9, 125.0, 124.9, 124.2, 121.0, 119.9 ppm. Signals corresponding to the C atoms bound to the B atoms are not visible. **^{11}B NMR** (128 MHz, CD_2Cl_2): δ = 0.3 ppm. **HRMS** (APCI) *m*/*z* calcd for $C_{36}H_{31}B_2N_2$ $[M+H]^+$ 513.2668, found: 513.2661.

Synthesis of H2-Et$_4$.

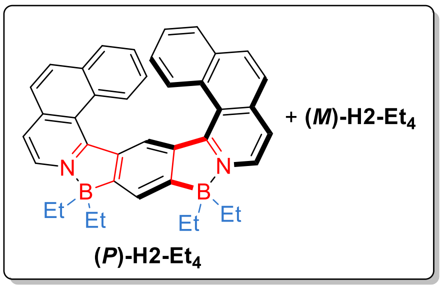

Compound **H2-Br$_4$** (50.0 mg, 64.8 µmol, 1.00 eq) was dissolved in a mixture of CH_2Cl_2 (1 mL) and toluene (1 mL) and $AlEt_3$ (1 M solution in *n*-hexane, 583 µL, 583 µmol, 4.50 eq.) was added dropwise under an inert atmosphere. The reaction was stirred for 2 h and subsequently quenched with water. The phases were separated and the aqueous layer was extracted with CH_2Cl_2. The combined organic layers were washed with water and brine, dried over $MgSO_4$, filtered and the solvent was removed and under reduced pressure. The crude product was purified via flash chromatography (silica, CH_2Cl_2/hexane 95:5) to obtain compound **H2-Et$_4$** (24 mg, 65%) as a yellow solid. **^{1}H NMR** (400 MHz, $CDCl_3$): δ = 10.00 (d, J = 0.7 Hz, 1H, ArH), 8.66 (d, J = 8.6 Hz, 2H, ArH), 8.33 (d, J = 5.9 Hz, 2H, ArH), 8.04 (d, J = 0.8 Hz, 1H, ArH), 8.00 (d, J = 8.6 Hz, 2H, ArH), 7.79 (dm, J = 7.9 Hz, 2H, ArH), 7.69 (d, J = 8.7 Hz, 2H, ArH), 7.59 (d, J = 6.0 Hz, 2H, ArH), 7.22 (ddd, J = 8.0, 7.1, 1.1 Hz, 2H, ArH), 6.55 (ddd, J = 8.4, 7.1, 1.3 Hz, 2H, ArH), 1.09–0.93 (m, 4H, CH_2), 0.77–0.66 (m, 4H, CH_2), 0.59 (t, J = 7.4 Hz, 12H, CH_3). **HRMS** (APCI): *m*/*z* calcd for $C_{40}H_{39}B_2N_2$ $[M+H]^+$ 569.3294, found: 569.3303. The compound is unstable.

Synthesis of H2-Ph$_4$.

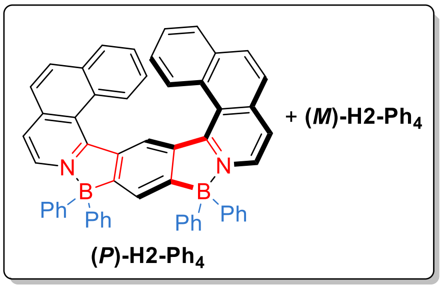

Compound **H2-Br$_4$** (100 mg, 129 µmol, 1.00 eq) was dissolved in a mixture of CH_2Cl_2 (2.5 mL) and toluene (2.5 mL) and $AlPh_3$ (1 M solution in *n*-hexane, 543 µL, 543 µmol, 4.20 eq.) was added dropwise under an inert atmosphere. After the reaction was stirred for 4 h at 90 °C it was brought to room temperature and quenched with water. The phases were separated and the aqueous layer was extracted with CH_2Cl_2. The combined organic layers were washed with water and brine, dried over $MgSO_4$, filtered and the solvent was removed under reduced pressure. The crude product was purified via flash chromatography (silica, CH_2Cl_2/hexane 85:15) to obtain compound **H2-Ph$_4$** (65 mg, 66%) as a yellow solid. **m.p.** > 400 °C. **^{1}H NMR** (400 MHz, CD_2Cl_2): δ = 10.16 (d, J = 0.6 Hz, 1H, ArH), 8.64 (d, J = 8.4 Hz, 2H, ArH), 8.42 (d, J = 6.0 Hz, 2H, ArH), 8.17 (d, J = 0.7 Hz, 1H, Ar-H), 8.10 (d, J = 8.6 Hz, 2H, ArH), 7.86 (dd, J = 8.0, 1.2 Hz, 2H, ArH), 7.75 (d, J = 8.7 Hz, 2H, ArH), 7.69 (d, J = 6.1 Hz, 2H, ArH), 7.39–7.06 (m, 22H, ArH), 6.59 ppm (ddd, J = 8.4, 7.1, 1.3, 2H, ArH). **^{13}C NMR** (100 MHz, CD_2Cl_2): δ = 157.7, 140.3, 138.4, 136.7, 134.8, 133.8, 132.9, 129.5, 128.8, 128.4, 128.2, 127.9, 126.1, 125.3, 124.9, 124.5, 121.0, 120.4 ppm. Signals corresponding to the C atoms bound to the B atoms are not visible. **^{11}B NMR** (128 MHz, CD_2Cl_2): δ = 4.6 ppm. **HRMS** (APCI) *m*/*z* calcd for $C_{56}H_{39}B_2N_2$ $[M+H]^+$ 761.3294, found 761.3260.

3. NMR and MS spectra

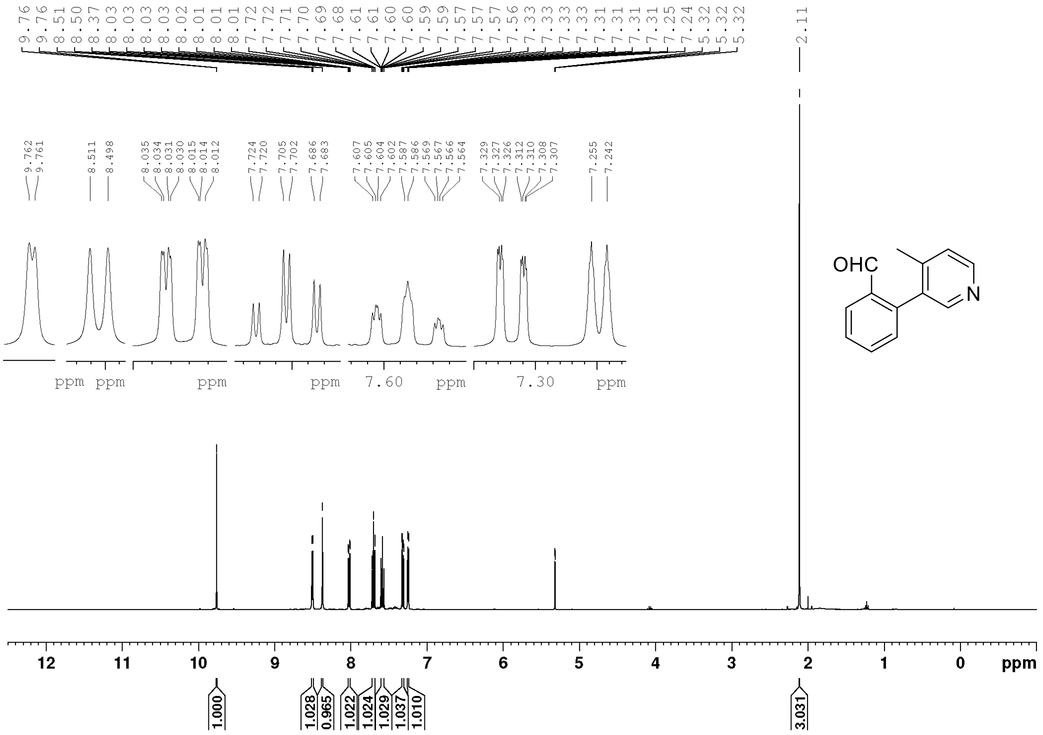

Figure S1. ^{1}H NMR of compound **3** (400 MHz, CD_2Cl_2, 25 °C).

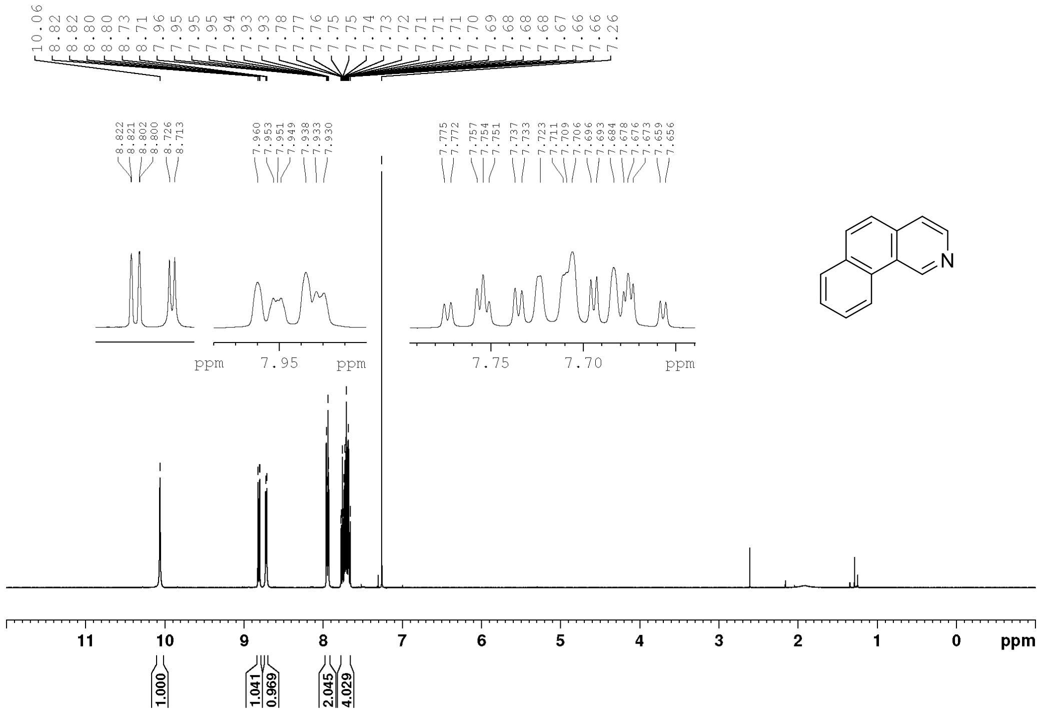

Figure S2. ^{1}H NMR of compound **BIQ** (400 MHz, $CDCl_3$, 25 °C).

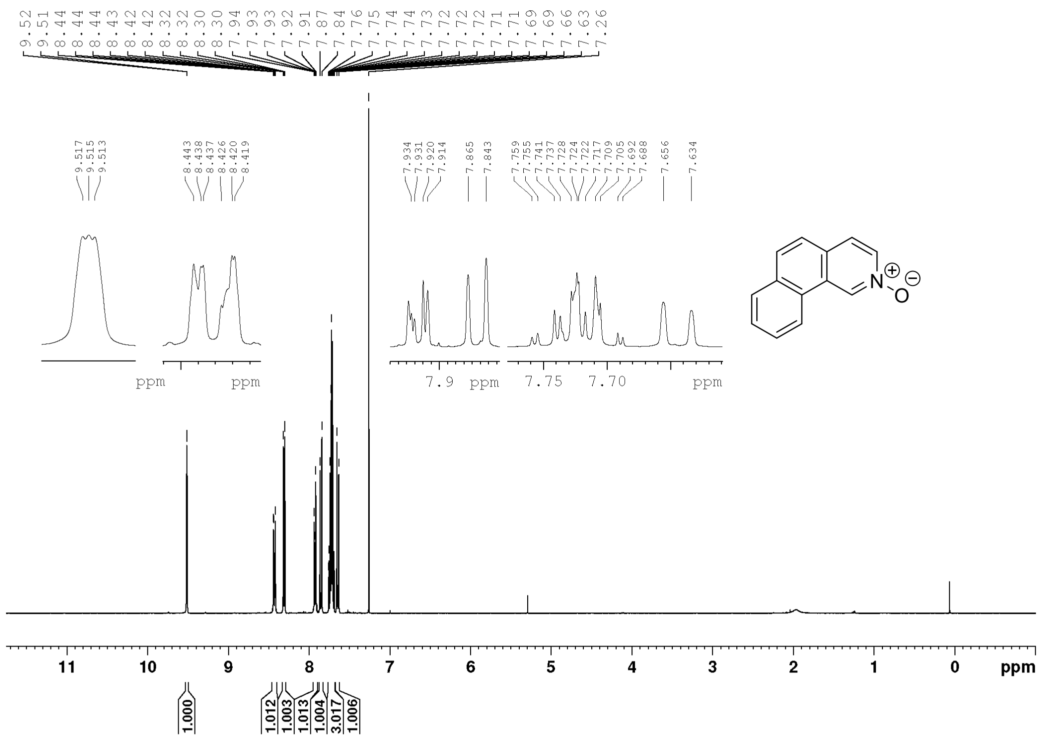

Figure S3. ^{1}H NMR of compound **BIQ-NOx** (400 MHz, $CDCl_3$, 25 °C).

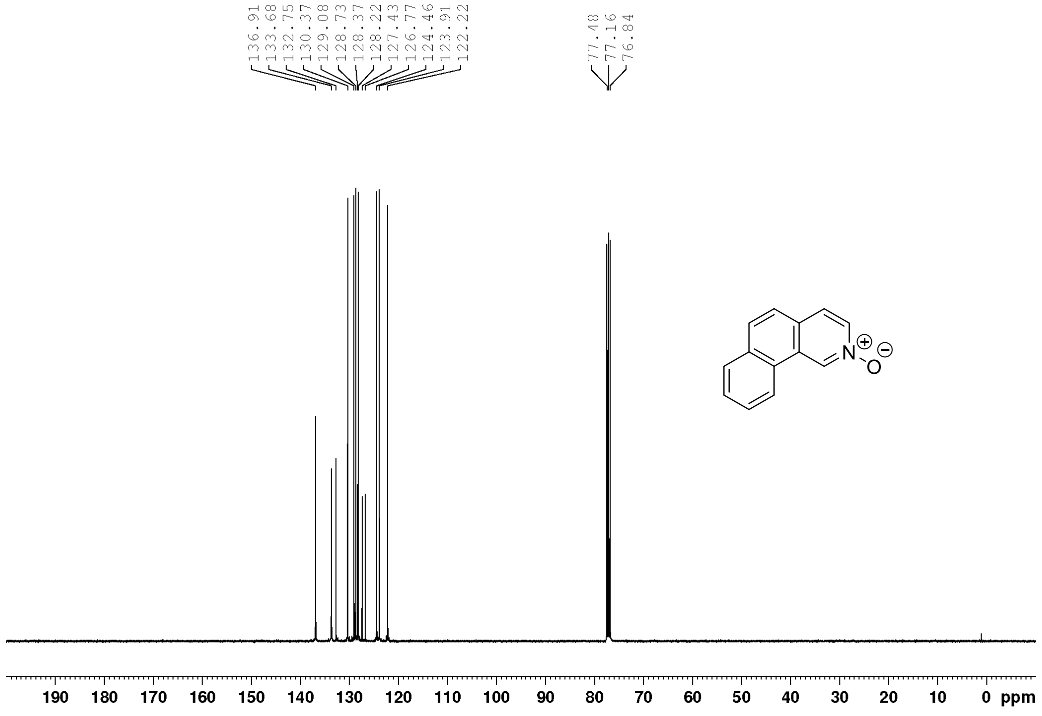

Figure S4. ^{13}C NMR of compound **BIQ-NOx** (101 MHz, $CDCl_3$, 25 °C).

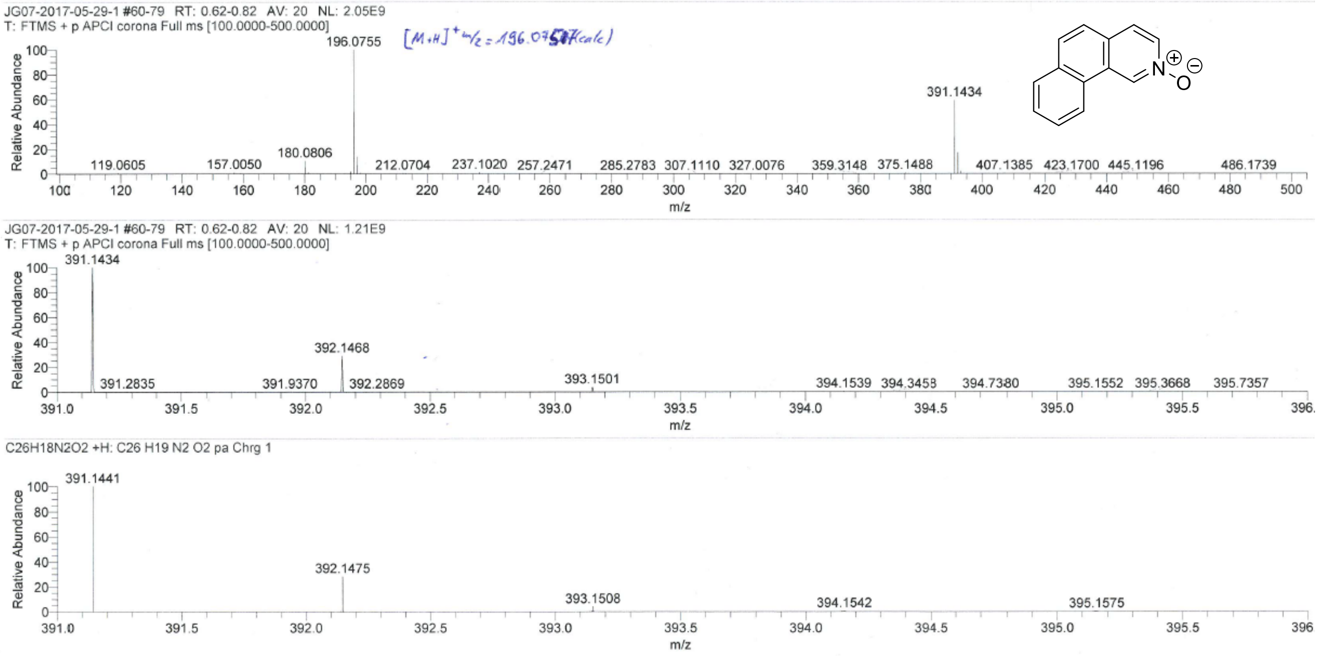

Figure S5. HRMS (APCI) spectrum of compound **BIQ-NOx**.

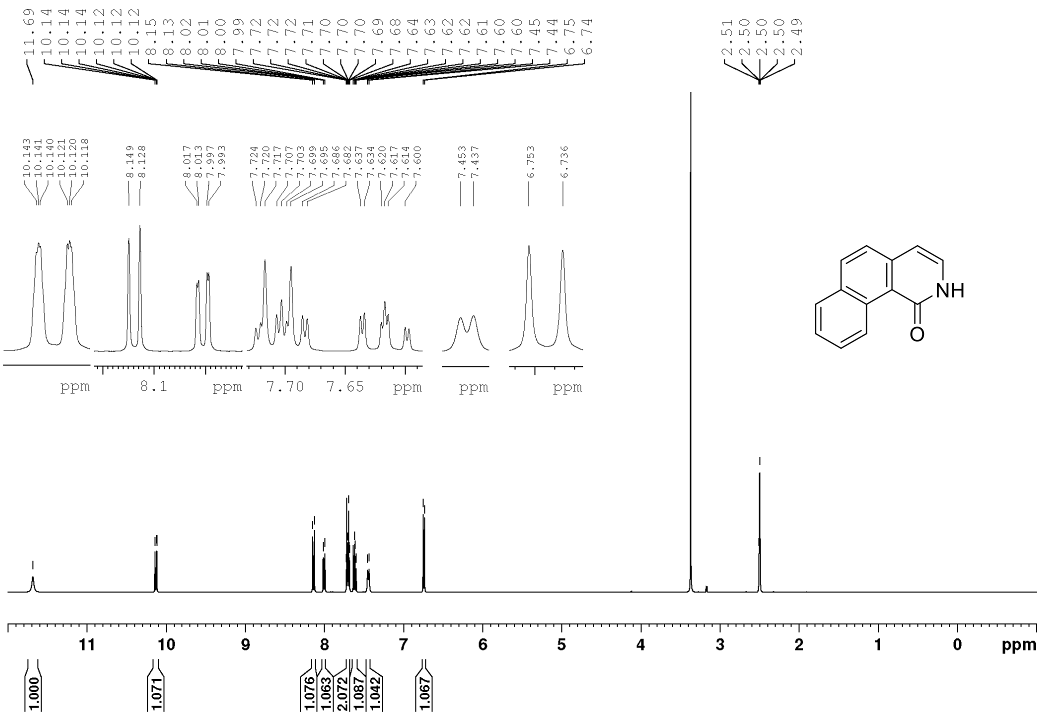

Figure S6. ^{1}H NMR of compound **4** (400 MHz, DMSO-d_6, 25 °C).

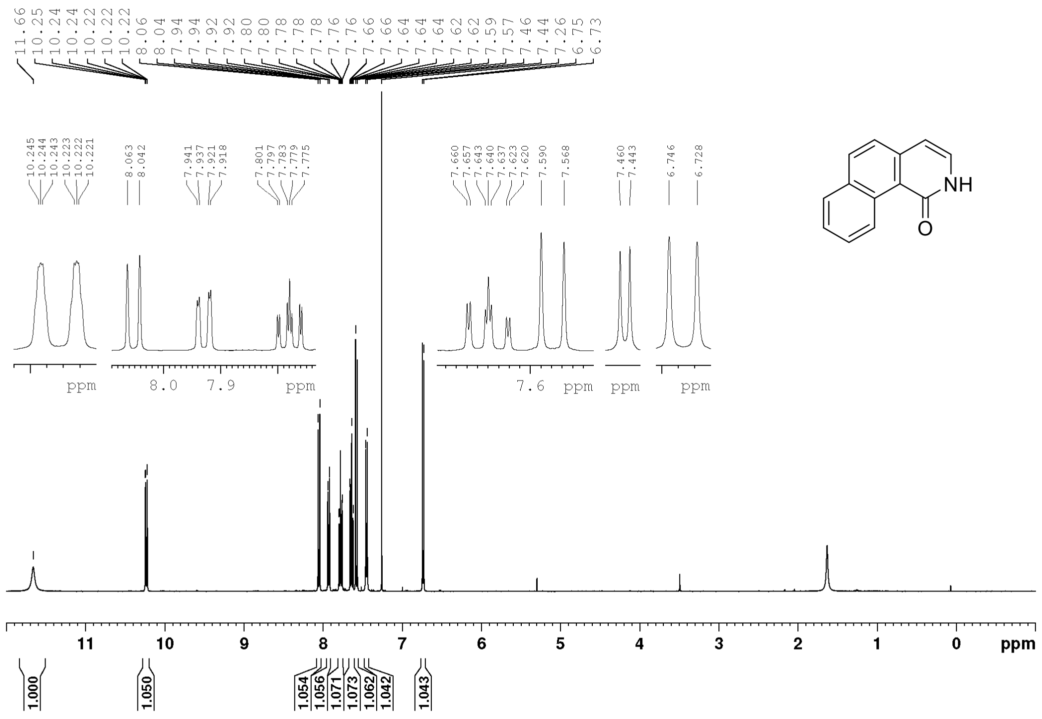

Figure S7. ^{1}H NMR of compound **4** (400 MHz, $CDCl_3$, 25 °C).

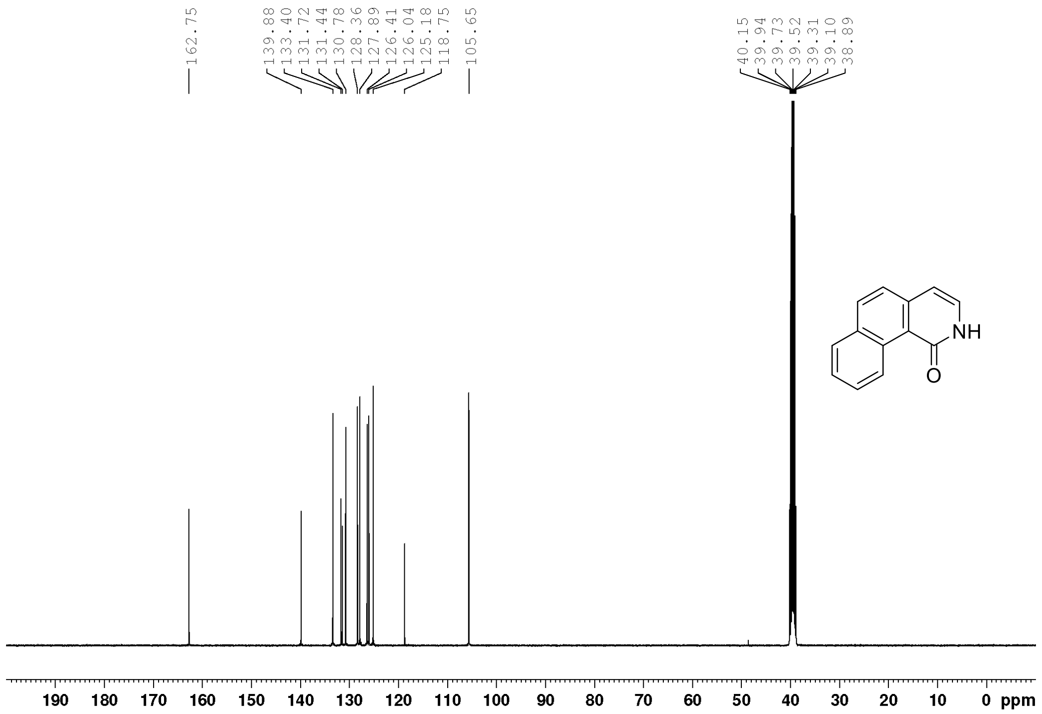

Figure S8. ^{13}C NMR of compound **4** (101 MHz, DMSO-d_6, 25 °C).

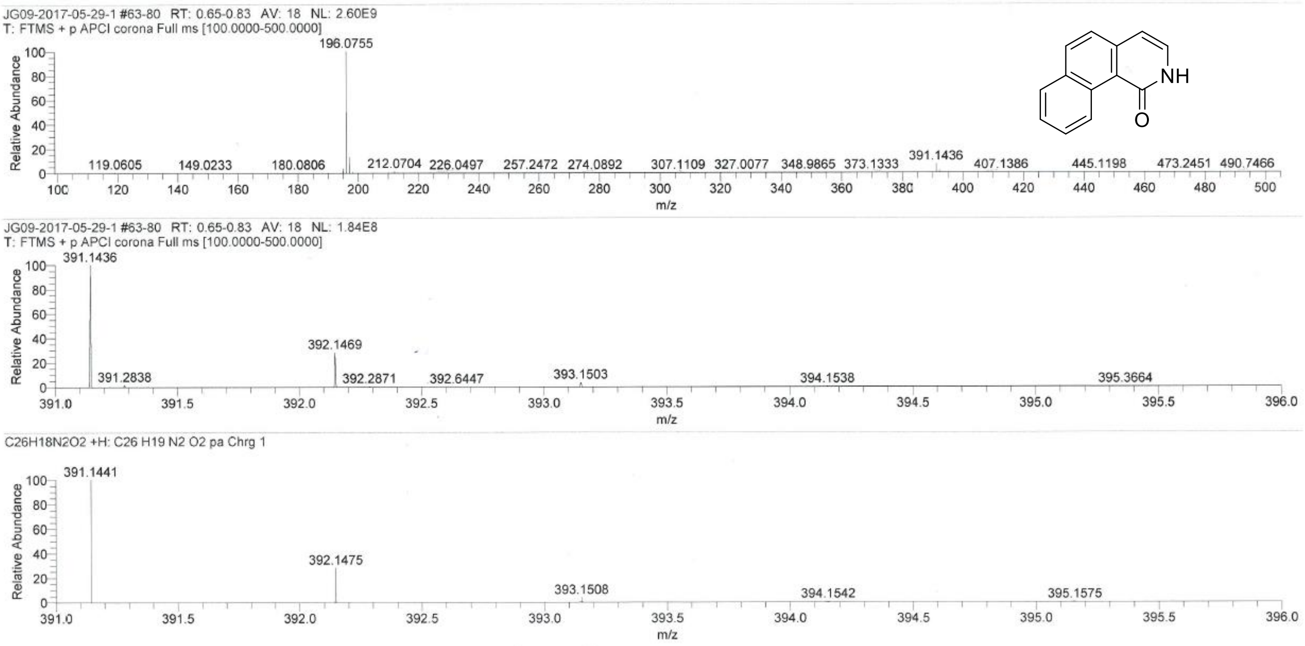

Figure S9. HRMS (APCI) spectrum of compound **4**.

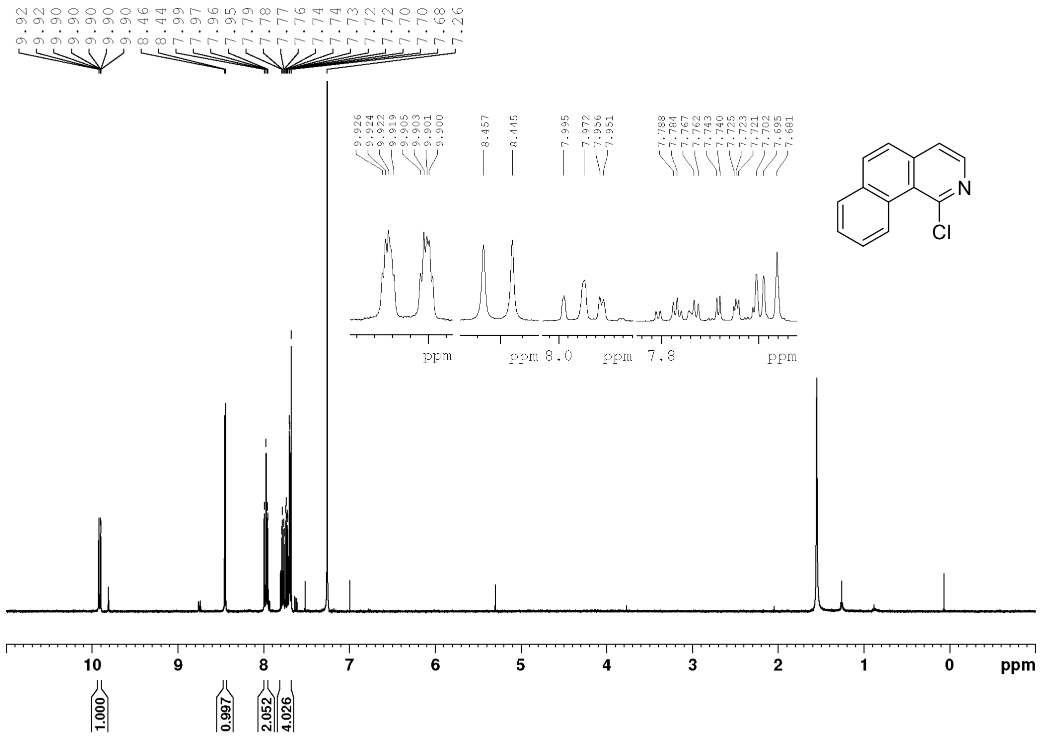

Figure S10. ^{1}H NMR of compound **BIQ-Cl** (method A) (400 MHz, $CDCl_3$, 25 °C).

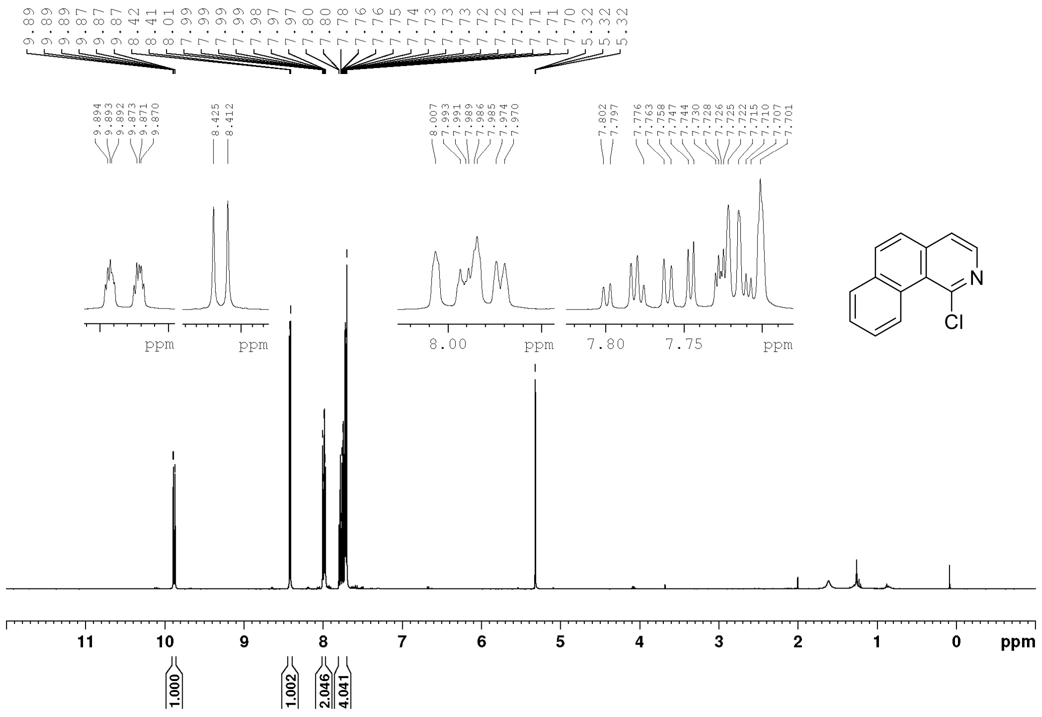

Figure S11. ^{1}H NMR of compound **BIQ-Cl** (method B) (400 MHz, CD_2Cl_2, 25 °C).

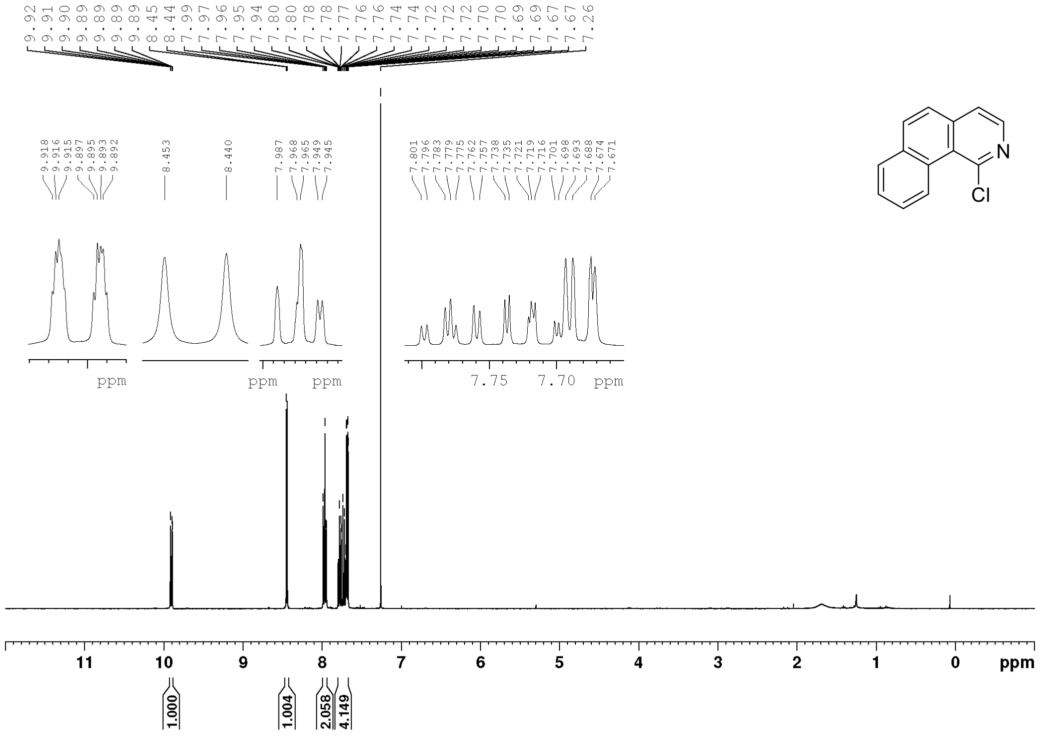

Figure S12. ^{1}H NMR of compound **BIQ-Cl** (method B) (400 MHz, $CDCl_3$, 25 °C).

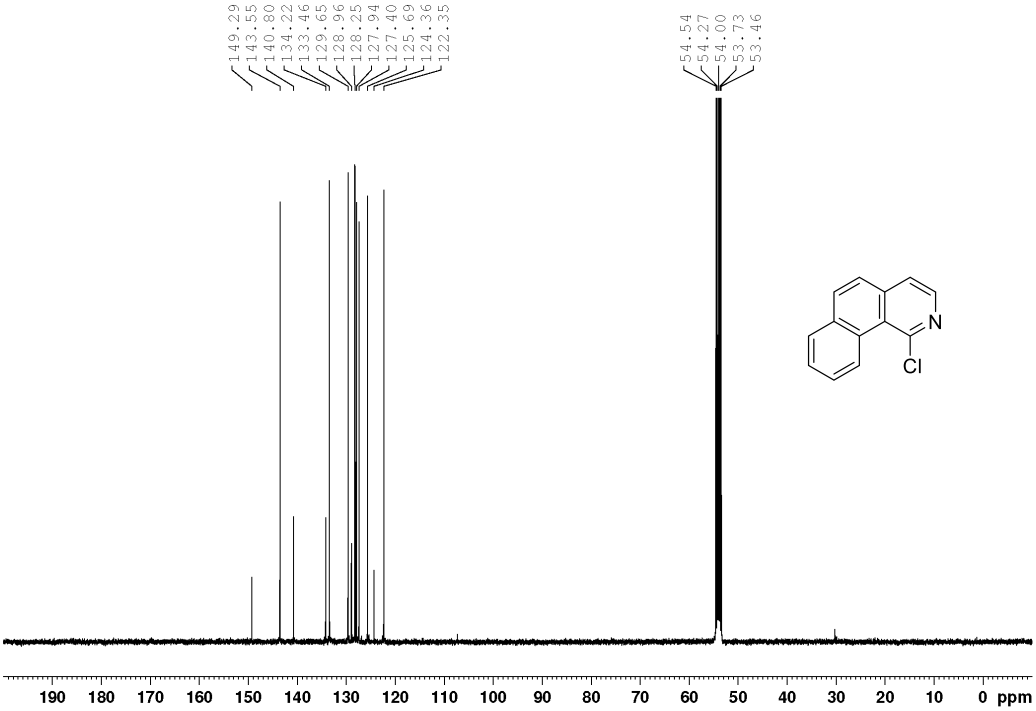

Figure S13. ^{13}C NMR of compound **BIQ-Cl** (method B) (101 MHz, CD_2Cl_2, 25 °C).

Acquisition Parameter

Source Type	ESI	Ion Polarity	Positive	Set Nebulizer	0.2 Bar
Focus	Not active	Set Funnel 1 RF	70.0 Vpp	Set Dry Heater	200 °C
Scan Begin	50 m/z	Set Funnel 2 RF	120.0 Vpp	Set Dry Gas	4.0 l/min
Scan End	2500 m/z	Set Hexapole RF	120.0 Vpp	Set Divert Valve	Source

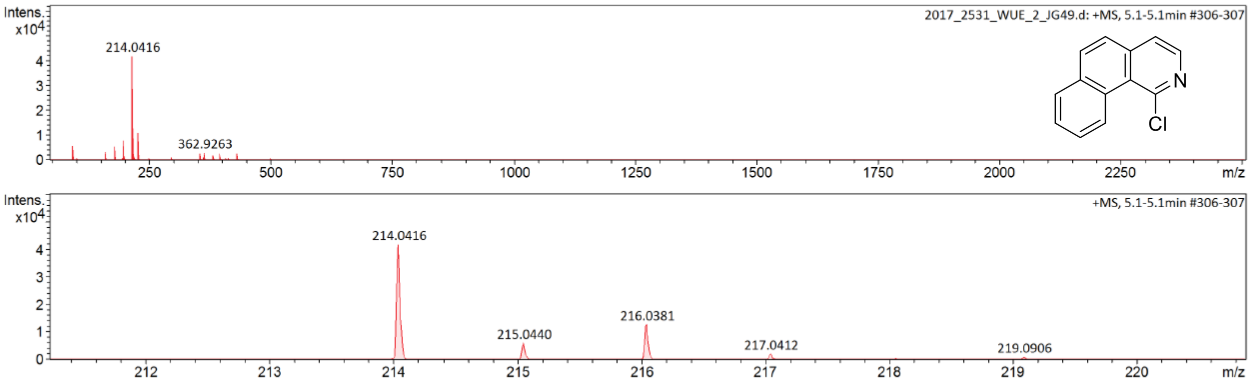

Meas. m/z	#	Ion Formula	m/z	err [ppm]	mSigma	# mSigma	Score	rdb	e⁻ Conf	N-Rule
214.0416	1	C13H9ClN	214.0418	0.9	15.9	1	100.00	9.5	even	ok

Figure S14. HRMS (ESI) of compound **BIQ-Cl** (method B).

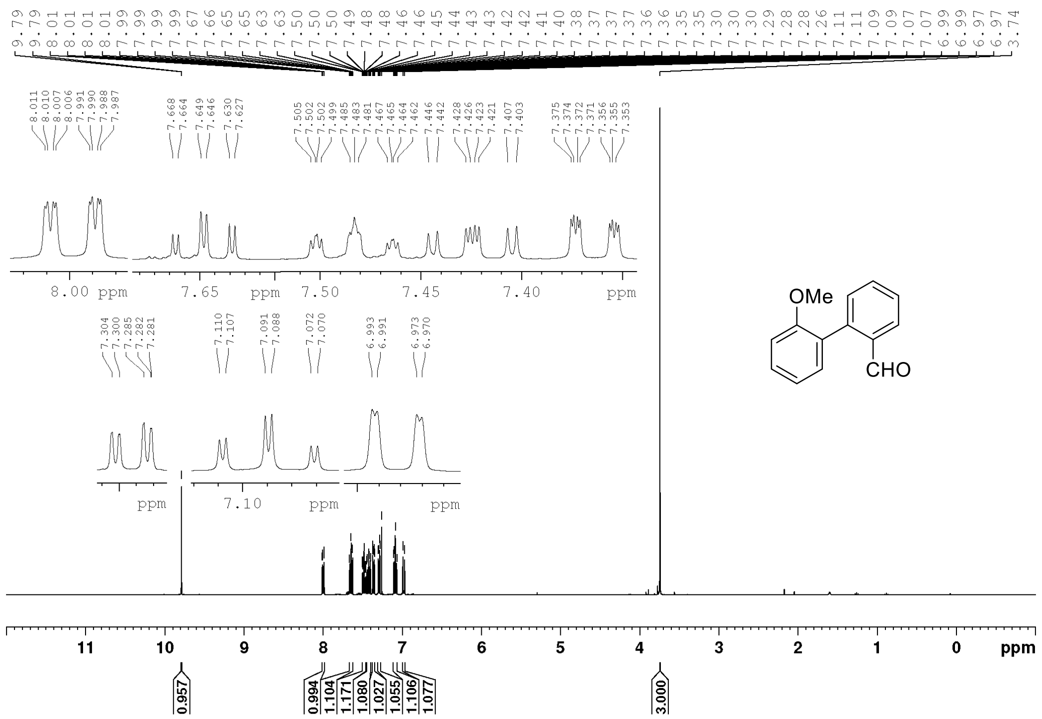

Figure S15. ^{1}H NMR of compound **7** (400 MHz, $CDCl_3$, 25 °C).

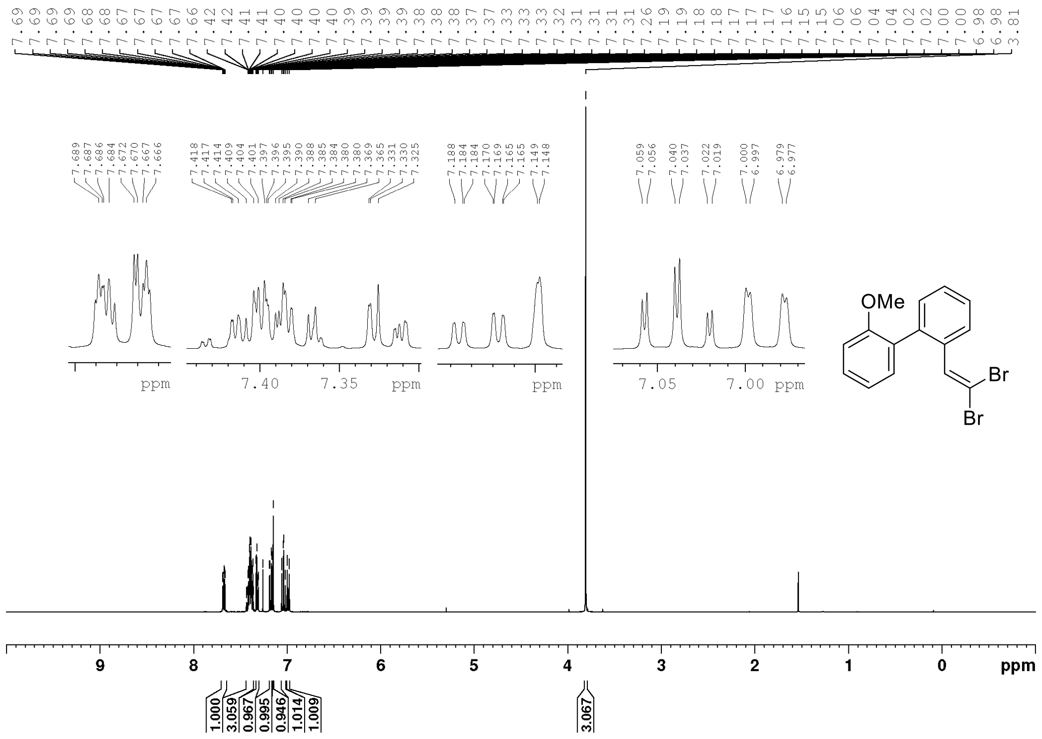

Figure S16. ^{1}H NMR of compound **8** (400 MHz, $CDCl_3$, 25 °C).

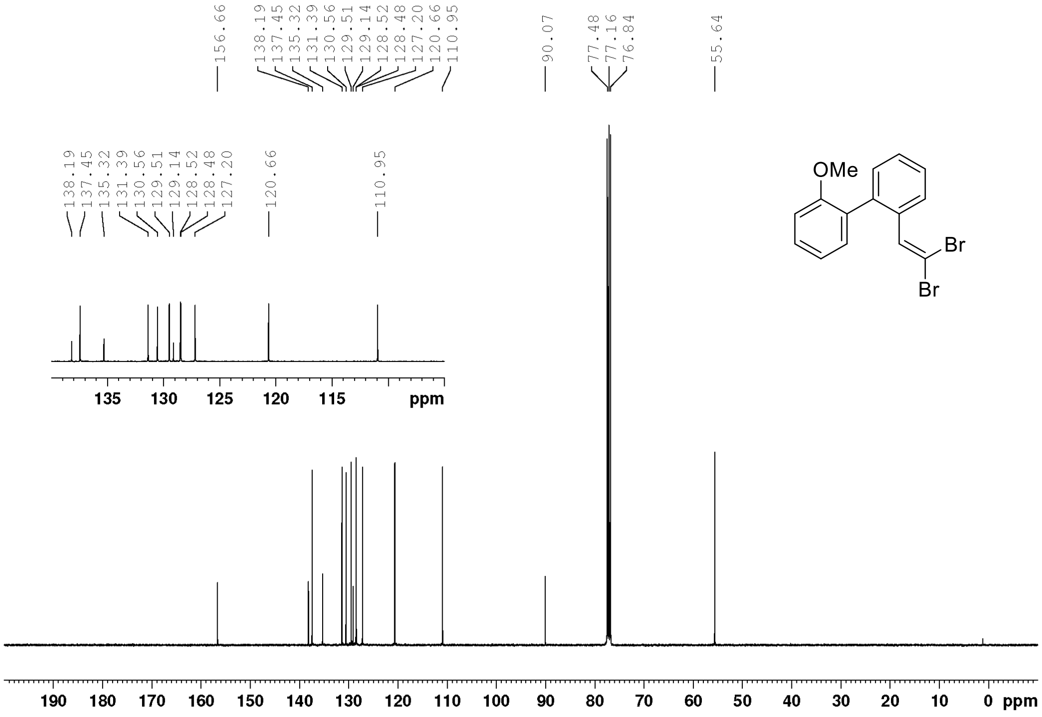

Figure S17. ^{13}C NMR of compound **8** (101 MHz, $CDCl_3$, 25 °C).

Acquisition Parameter

Source Type	APCI	Ion Polarity	Positive	Set Nebulizer	2.0 Bar
Focus	Not active	Set Funnel 1 RF	50.0 Vpp	Set Dry Heater	250 °C
Scan Begin	50 m/z	Set Funnel 2 RF	100.0 Vpp	Set Dry Gas	2.0 l/min
Scan End	2000 m/z	Set Hexapole RF	100.0 Vpp	Set Divert Valve	Source

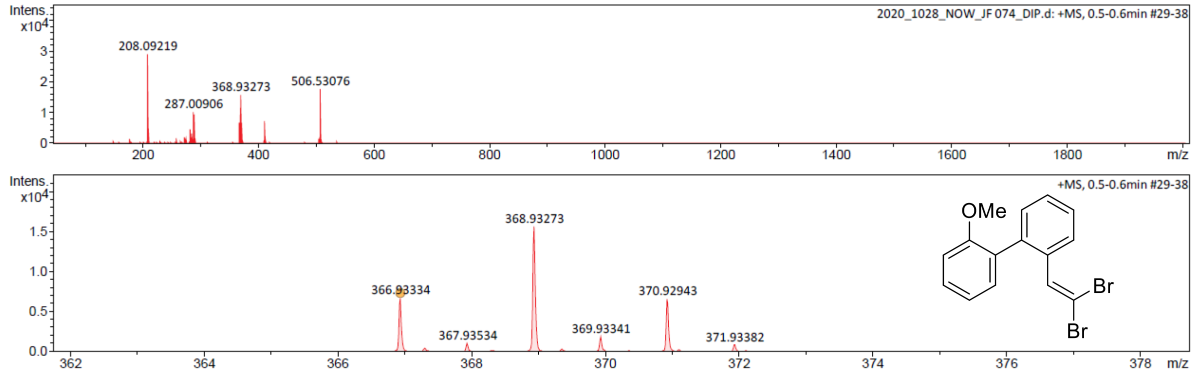

Meas. m/z	#	Ion Formula	m/z	err [ppm]	mSigma	# mSigma	Score	rdb	e⁻ Conf	N-Rule
366.93334	1	C15H13Br2O	366.93277	-1.56	51.9	1	100.00	8.5	even	ok

Figure S18. HRMS (APCI) of compound **8**.

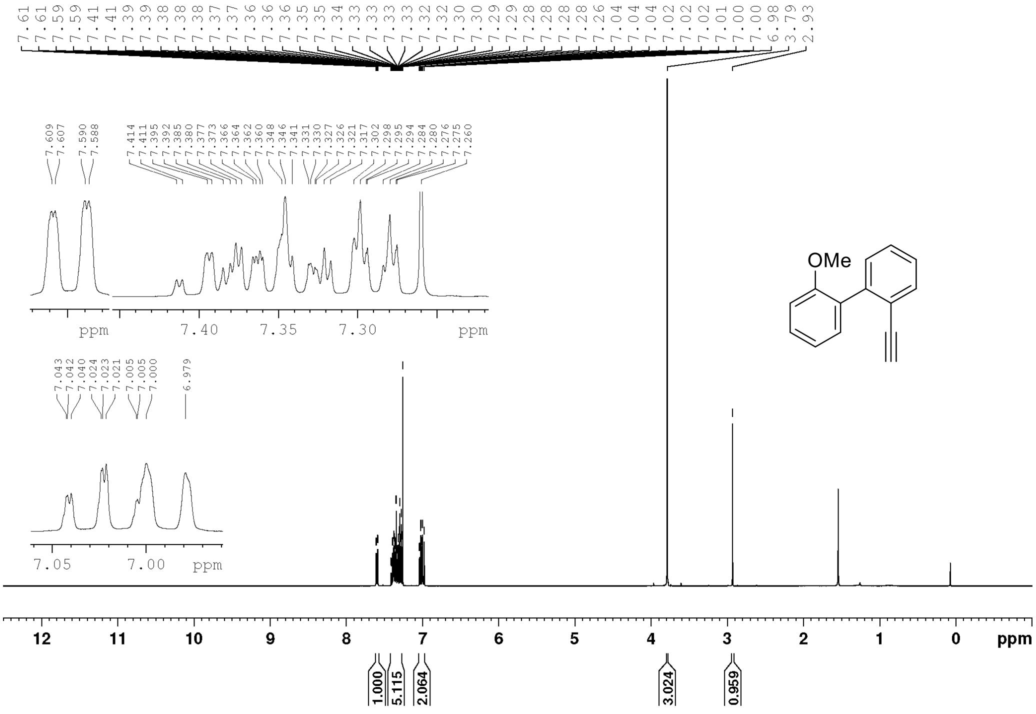

Figure S19. ^{1}H NMR of compound **9** (400 MHz, $CDCl_3$, 25 °C).

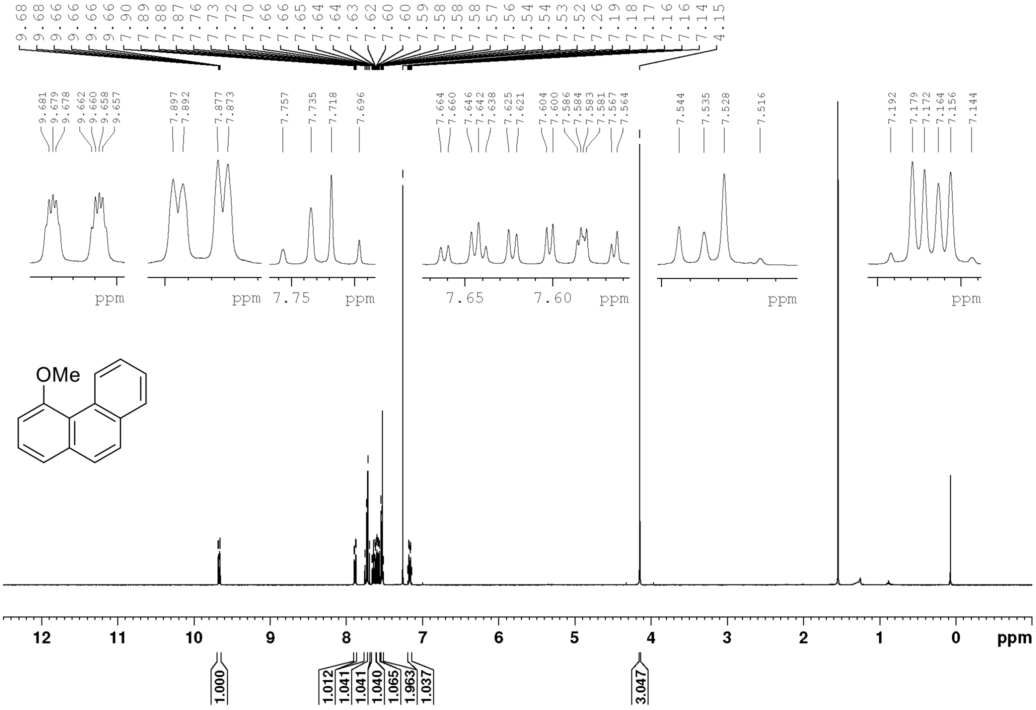

Figure S20. ^{1}H NMR of compound **PHE-OMe** (400 MHz, $CDCl_3$, 25 °C).

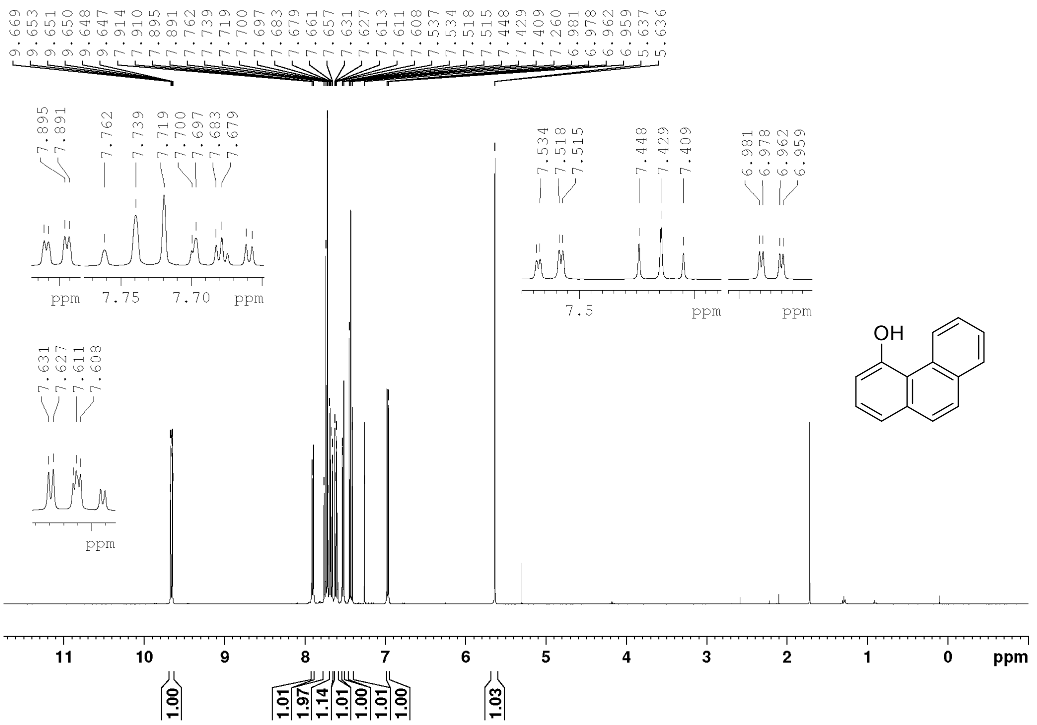

Figure S21. ^{1}H NMR of compound **PHE-OH** (400 MHz, $CDCl_3$, 25 °C).

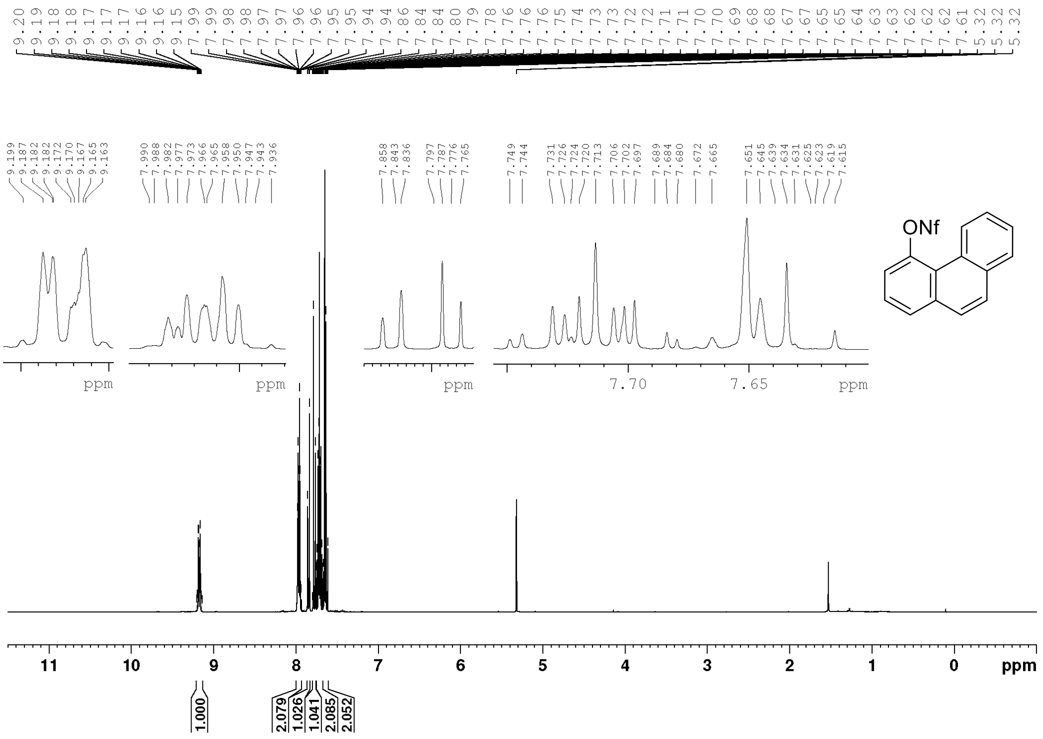

Figure S22. ^{1}H NMR of compound **PHE-ONf** (400 MHz, CD_2Cl_2, 25 °C).

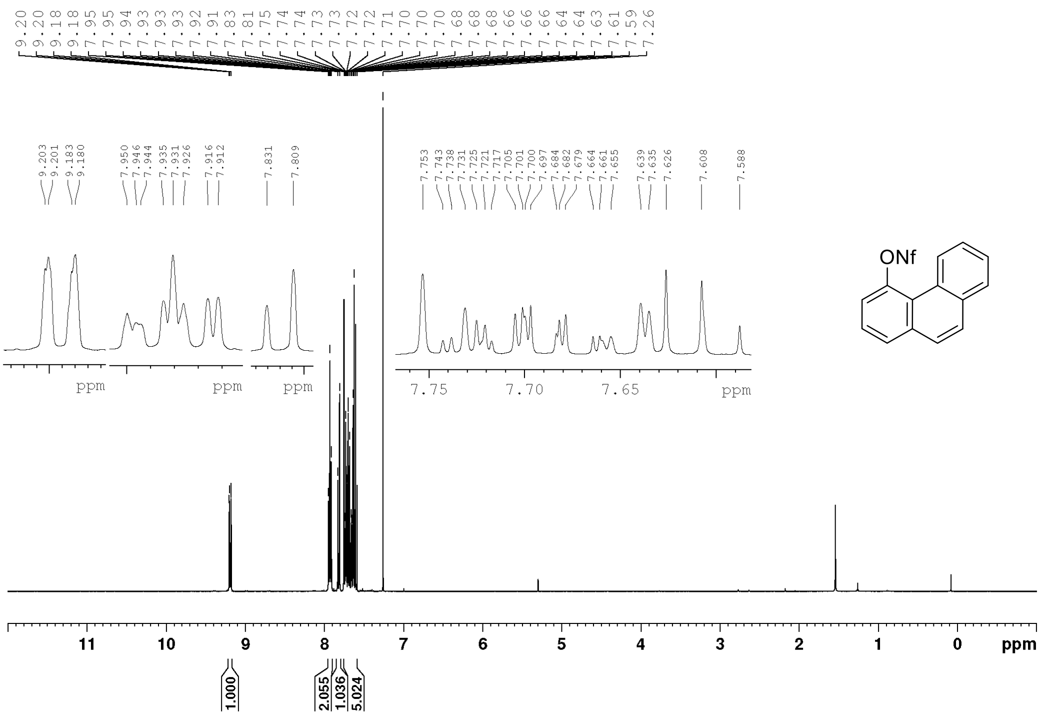

Figure S23. ^{1}H NMR of compound **PHE-ONf** (400 MHz, $CDCl_3$, 25 °C).

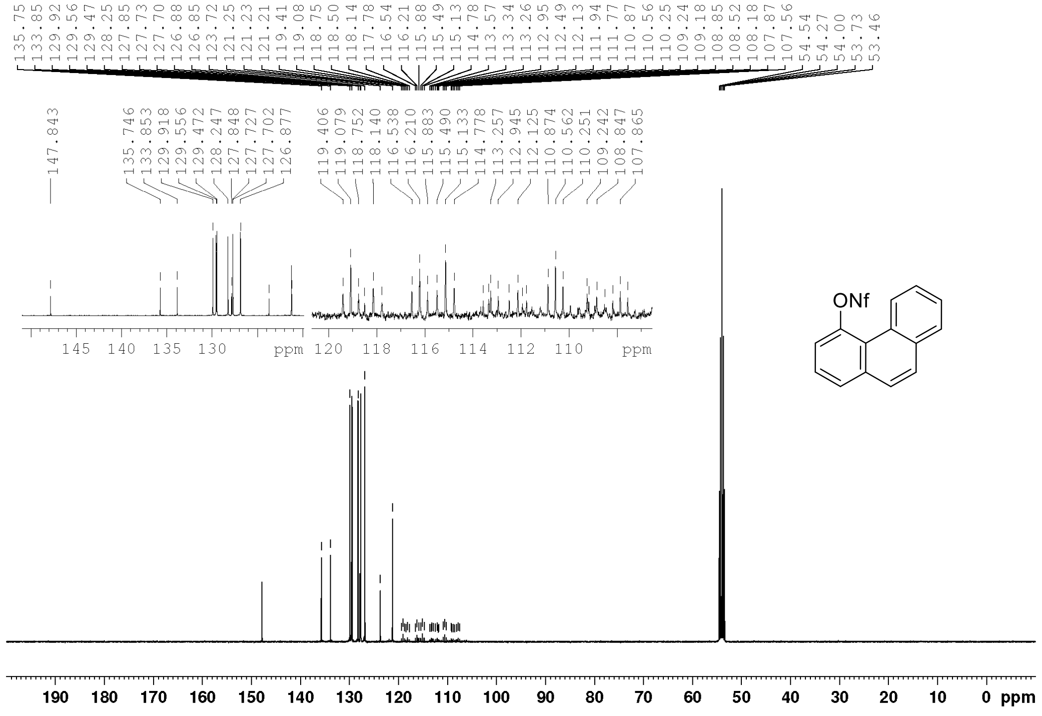

Figure S24. ^{13}C NMR of compound **PHE-ONf** (101 MHz, CD_2Cl_2, 25 °C).

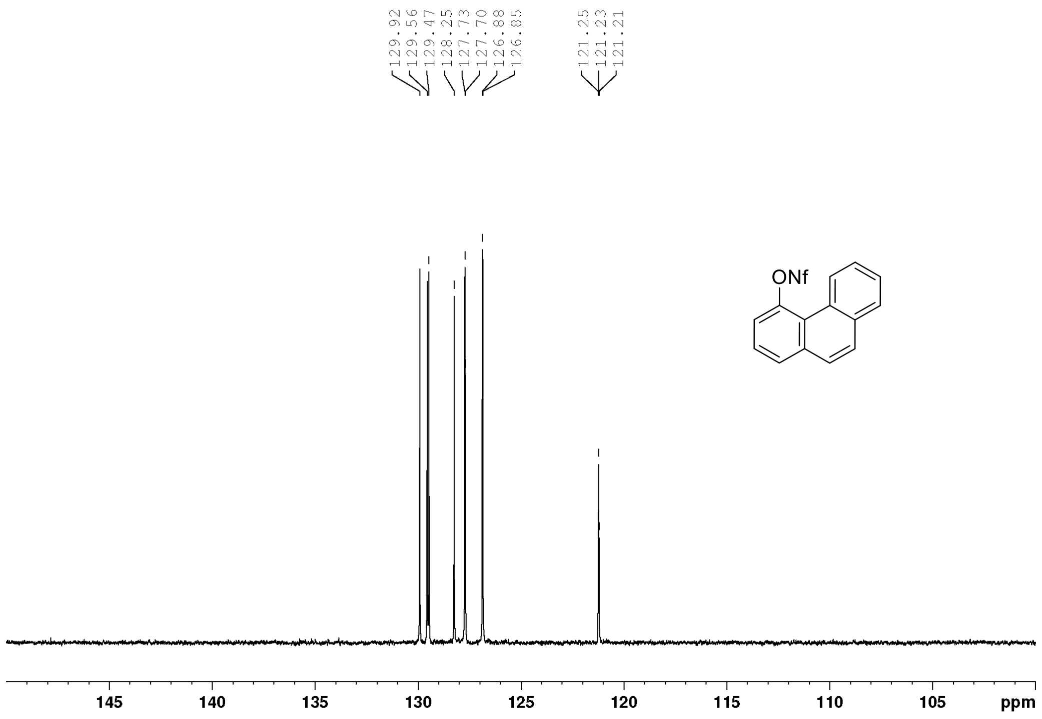

Figure S25. DEPT-135 spectrum of compound **PHE-ONf** (101 MHz, CD_2Cl_2, 25 °C).

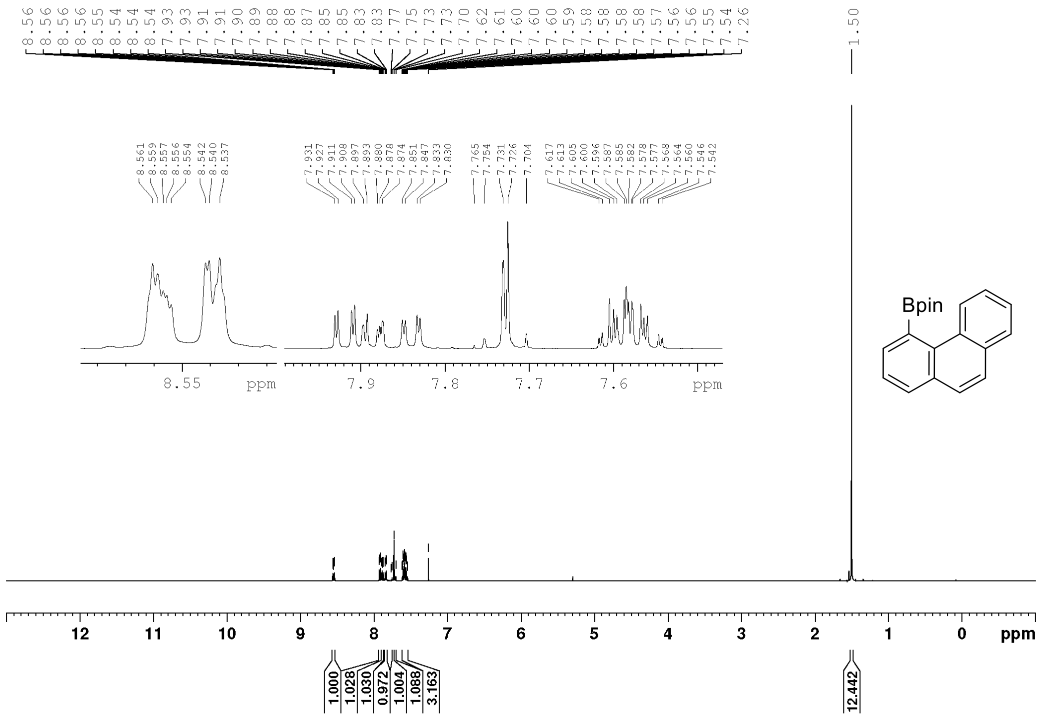

Figure S26. ^{1}H NMR of compound **PHE-Bpin** (400 MHz, $CDCl_3$, 25 °C).

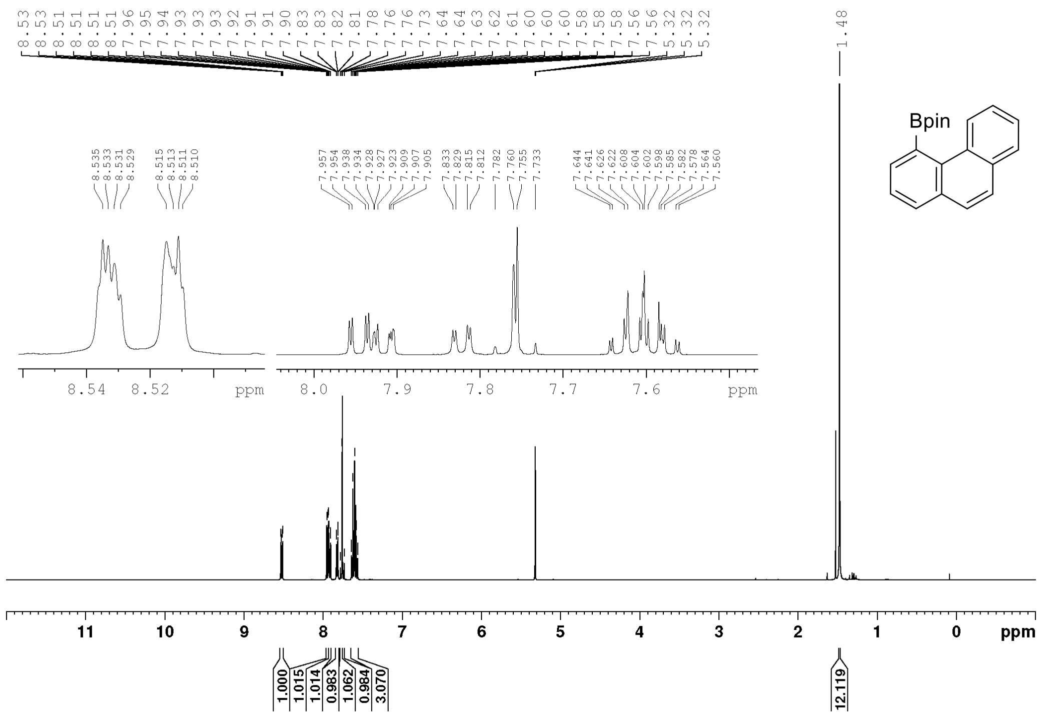

Figure S27. ^{1}H NMR of compound **PHE-Bpin** (400 MHz, CD_2Cl_2, 25 °C).

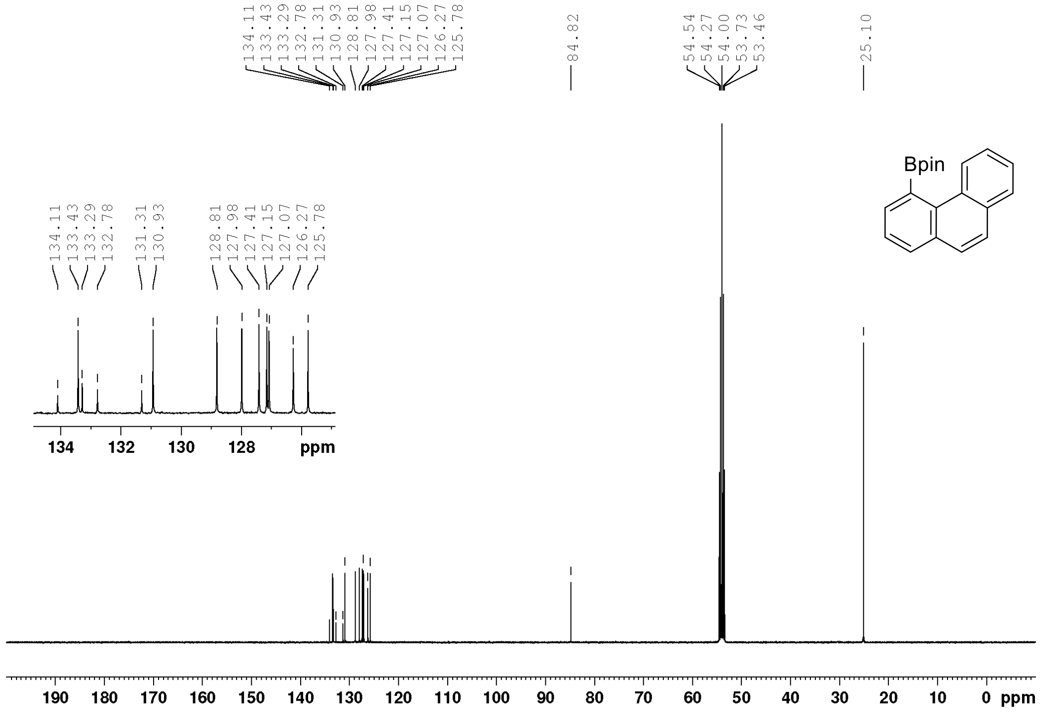

Figure S28. ^{13}C NMR of compound **PHE-Bpin** (101 MHz, CD_2Cl_2, 25 °C).

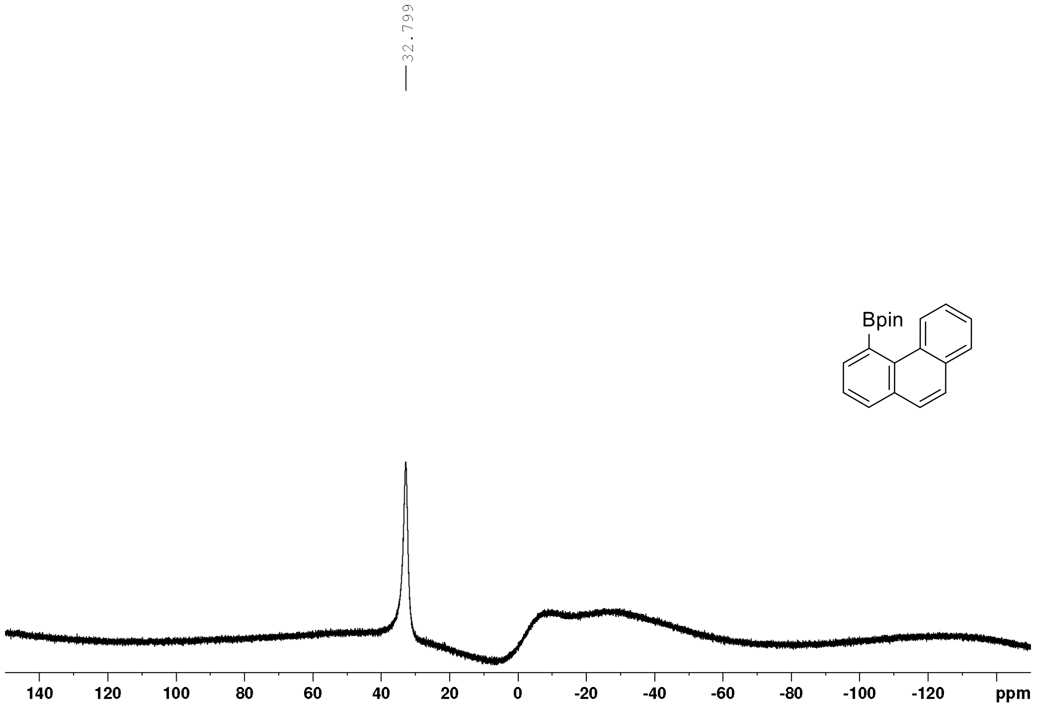

Figure S29. ^{11}B NMR of compound **PHE-Bpin** (128 MHz, CD_2Cl_2, 25 °C)

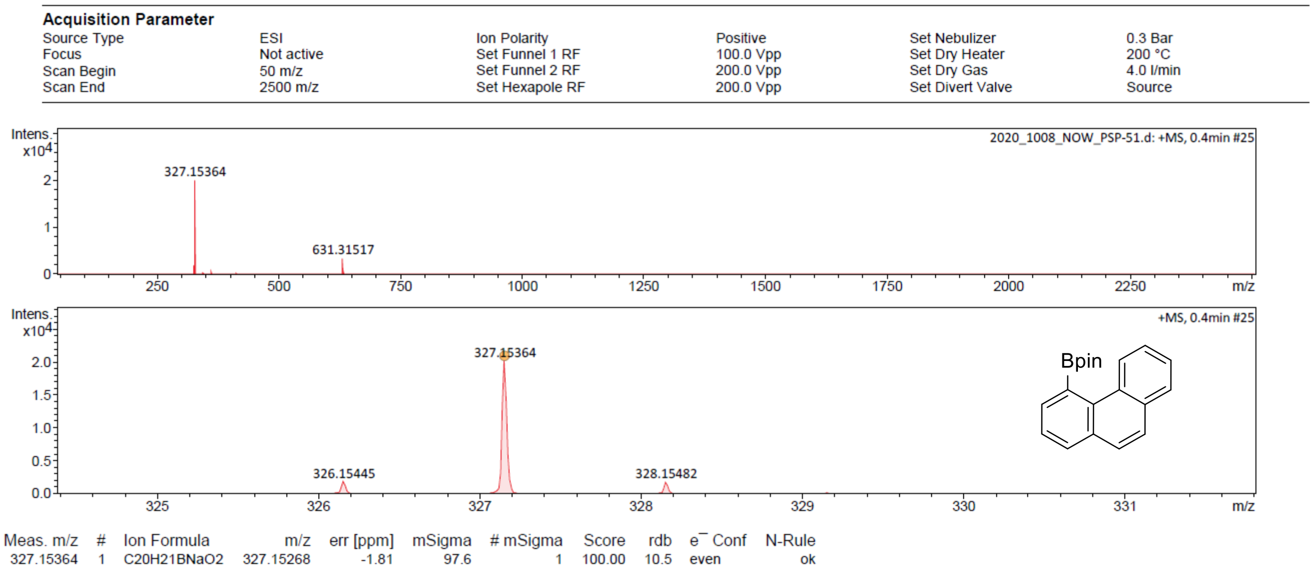

Figure S30. HRMS (ESI) spectrum of compound **PHE-Bpin**.

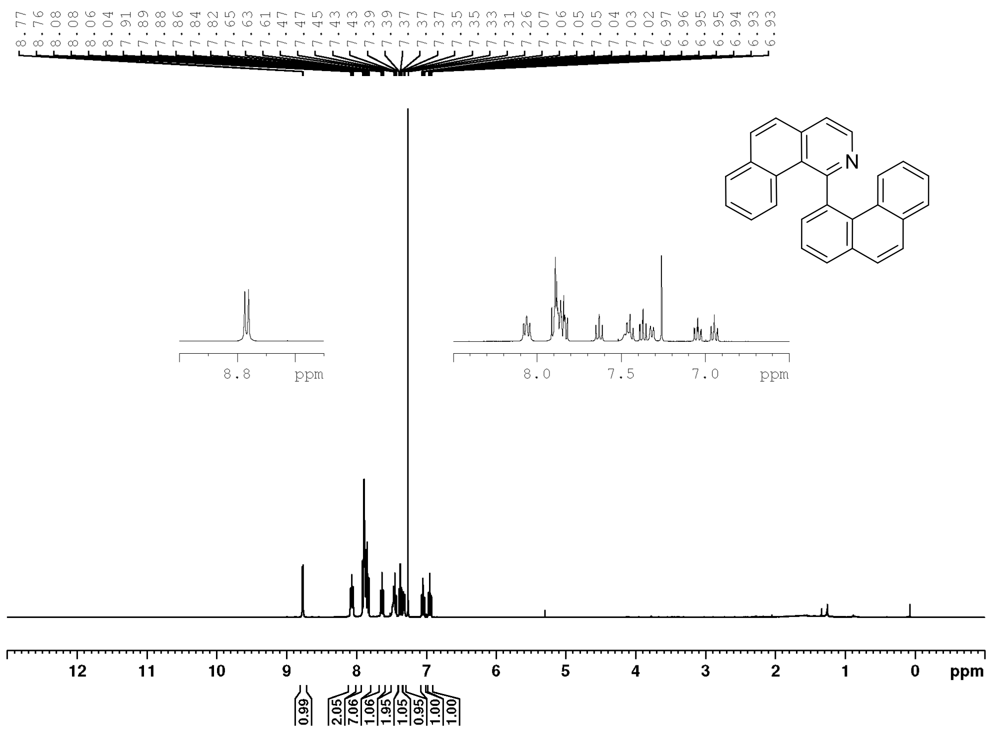

Figure S31. ^{1}H NMR of compound **BA** (400 MHz, $CDCl_3$, 25 °C) (method A).

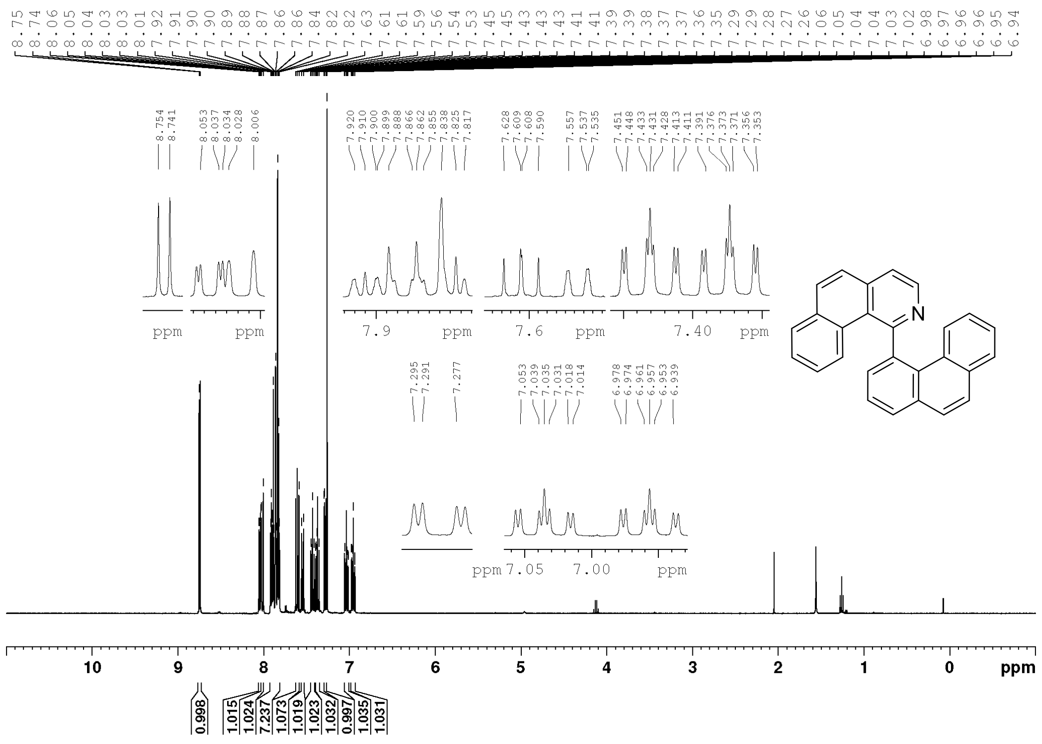

Figure S32. ^{1}H NMR of compound **BA** (400 MHz, $CDCl_3$, 25 °C) (method B).

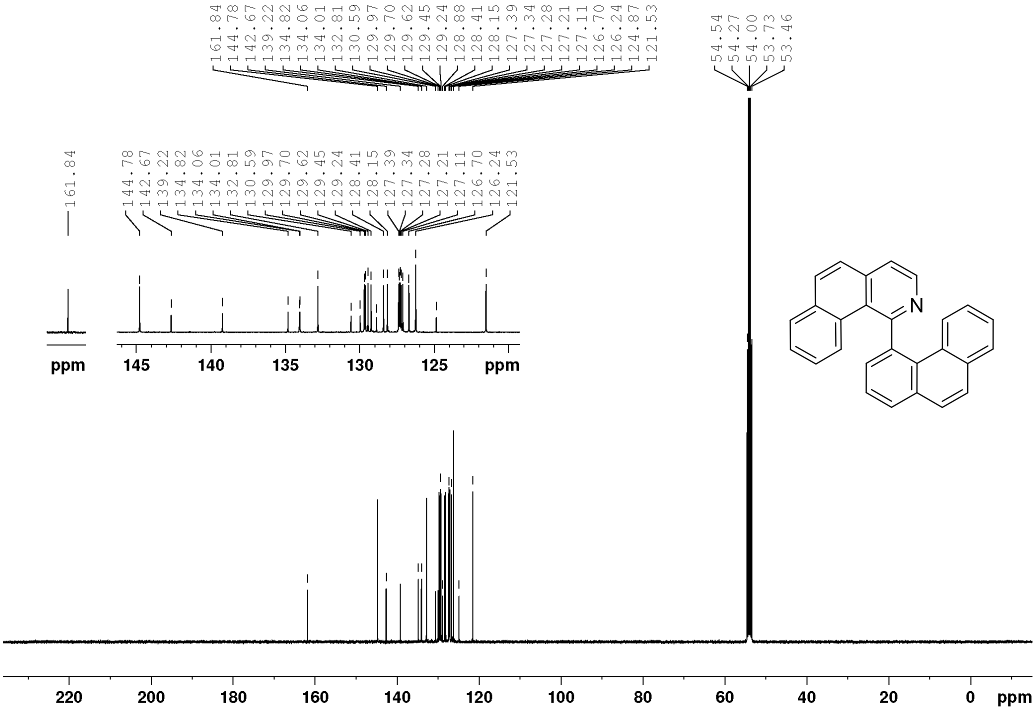

Figure S33. ^{13}C NMR of compound **BA** (101 MHz, $CDCl_3$, 25 °C) (method B).

Acquisition Parameter

Source Type	ESI	Ion Polarity	Positive	Set Nebulizer	0.3 Bar
Focus	Not active	Set Funnel 1 RF	200.0 Vpp	Set Dry Heater	200 °C
Scan Begin	50 m/z	Set Funnel 2 RF	300.0 Vpp	Set Dry Gas	3.5 l/min
Scan End	4000 m/z	Set Hexapole RF	400.0 Vpp	Set Divert Valve	Source

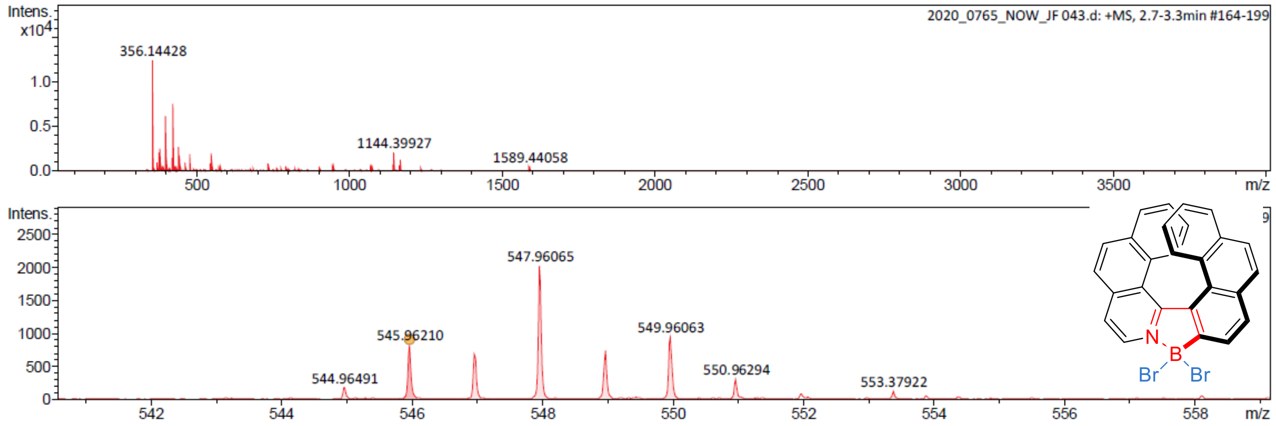

Meas. m/z	#	Ion Formula	m/z	err [ppm]	mSigma	# mSigma	Score	rdb	e⁻ Conf	N-Rule
545.96210	1	C27H16BBr2NNa	545.96347	3.39	41.6	2	100.00	19.5	even	ok

Figure S34. HRMS (ESI) spectrum of compound **H1-Br2**.

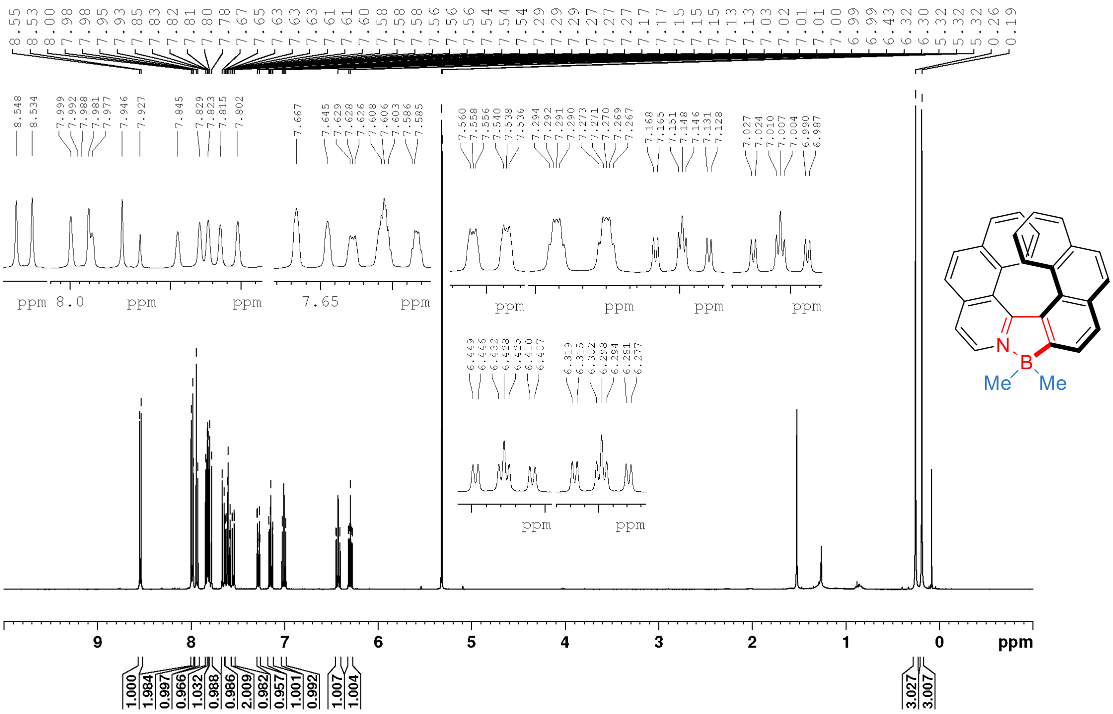

Figure S35. ^{1}H NMR of compound **H1-Me$_2$** (400 MHz, CD_2Cl_2, 25 °C).

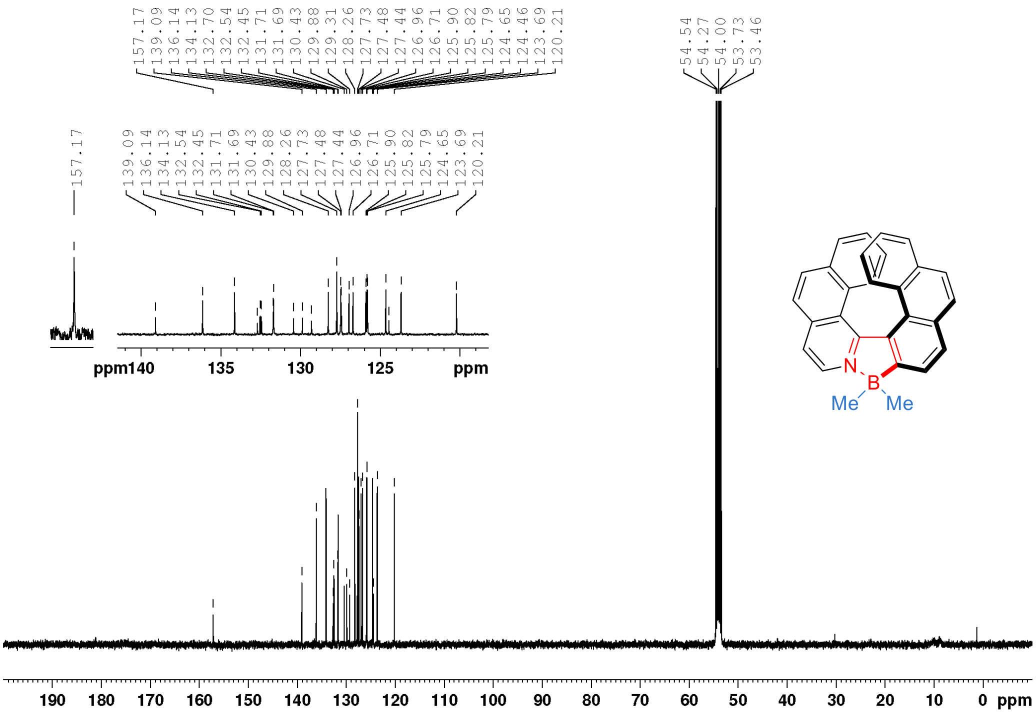

Figure S36. ^{13}C NMR of compound **H1-Me$_2$** (101 MHz, CD_2Cl_2, 25 °C).

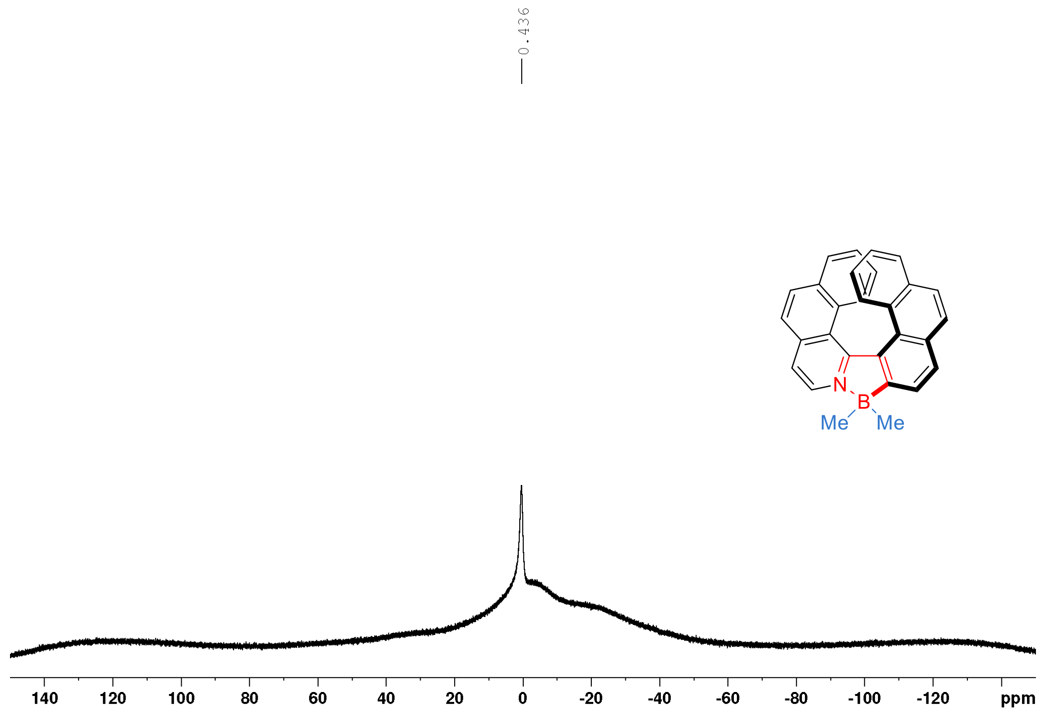

Figure S37. ^{11}B NMR of compound **H1-Me$_2$** (128 MHz, CD_2Cl_2, 25 °C).

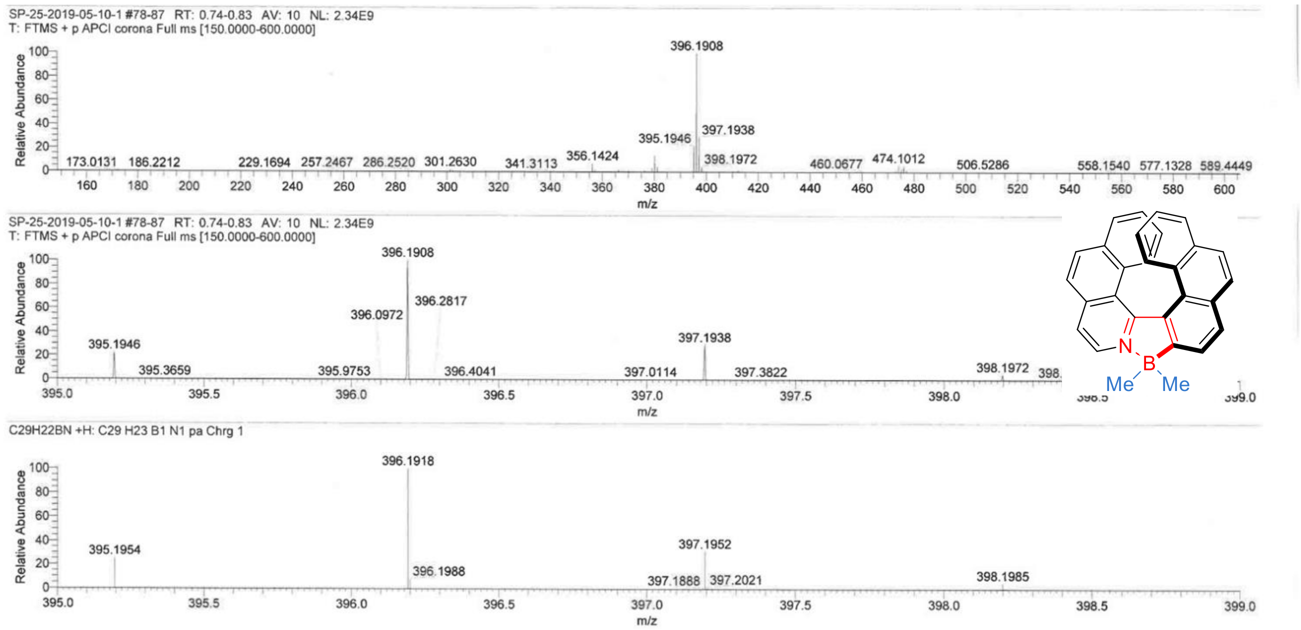

Figure S38. HRMS (APCI) spectrum of compound **H1-Me$_2$**.

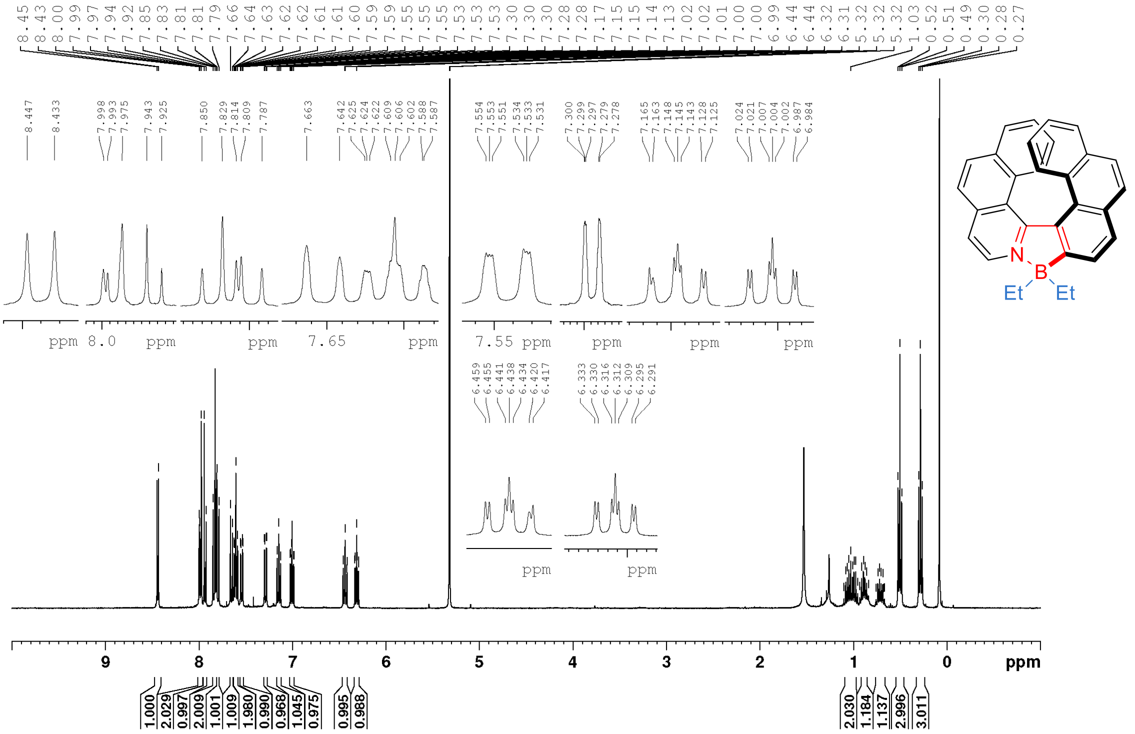

Figure S39. ^{1}H NMR of compound **H1-Et$_2$** (400 MHz, CD_2Cl_2, 25 °C) (method A).

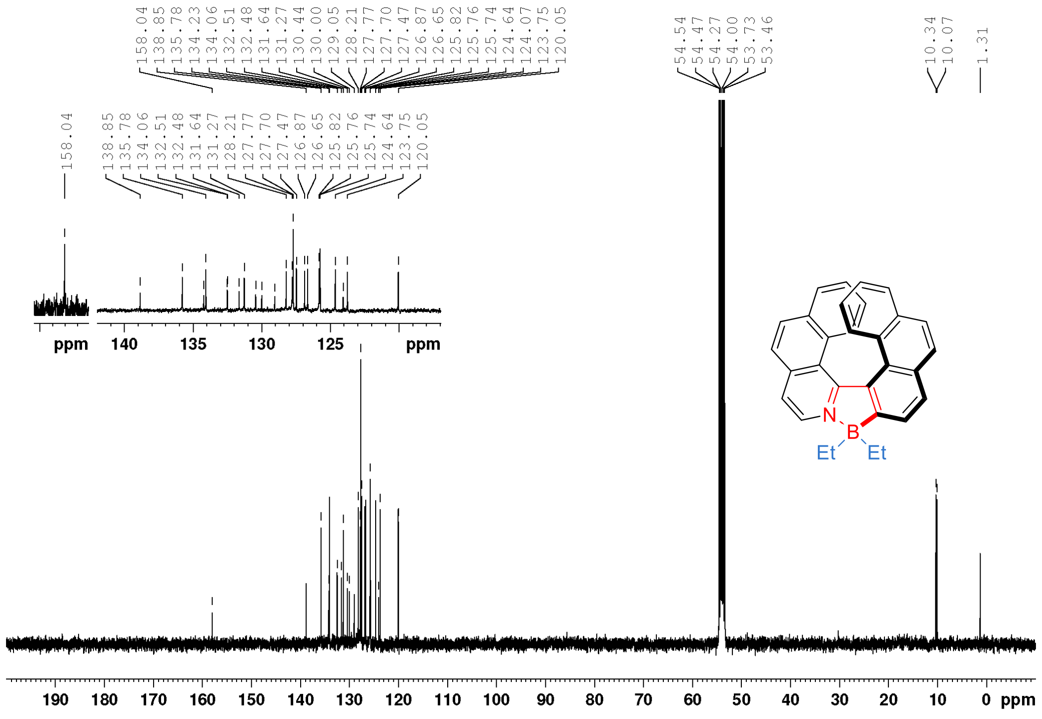

Figure S40. ^{13}C NMR of compound **H1-Et$_2$** (101 MHz, CD_2Cl_2, 25 °C) (method A).

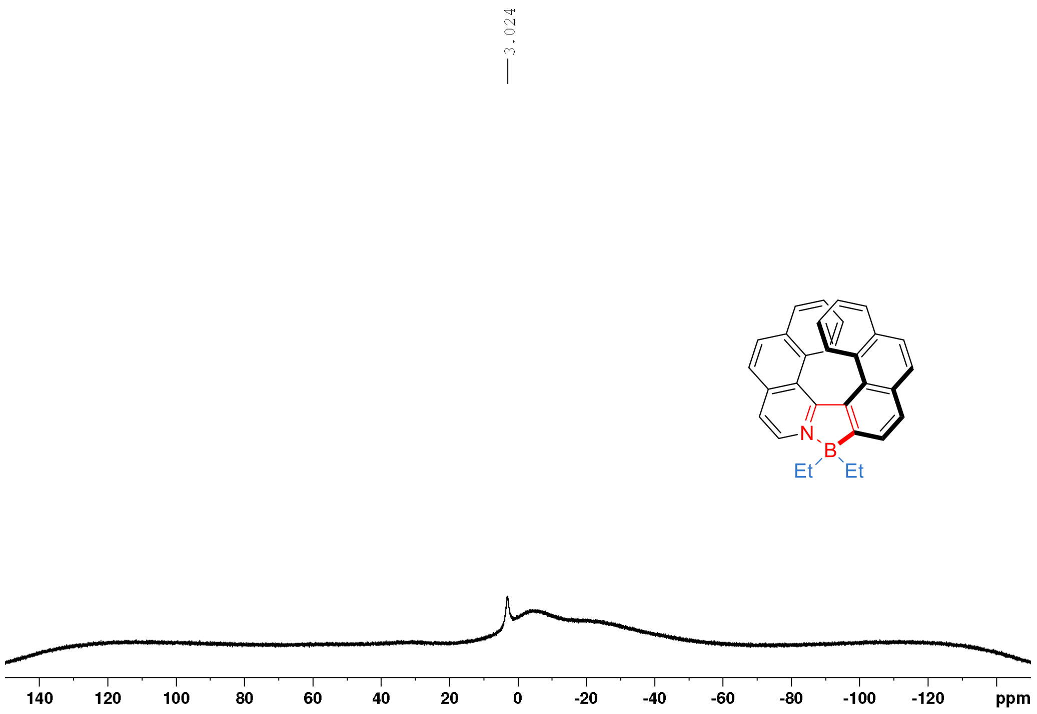

Figure S41. ^{11}B NMR of compound **H1-Et$_2$** (128 MHz, CD_2Cl_2, 25 °C) (method A).

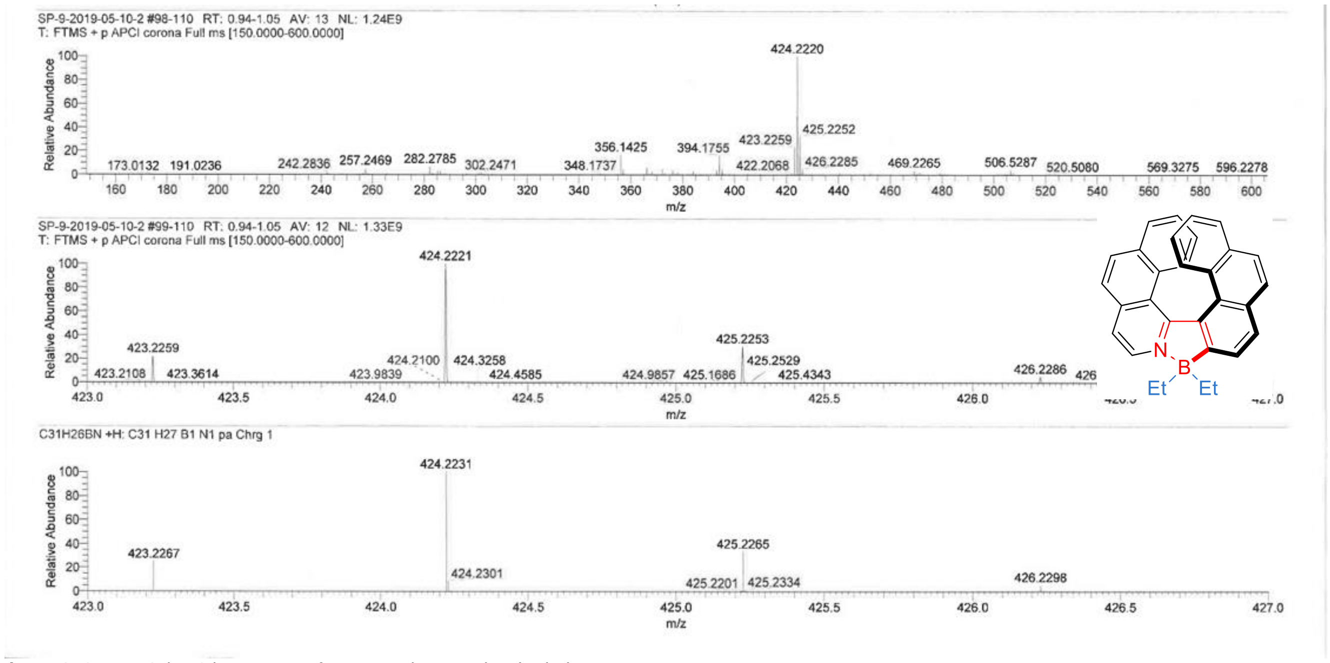

Figure S42. HRMS (APCI) spectrum of compound **H1-Et$_2$** (method A).

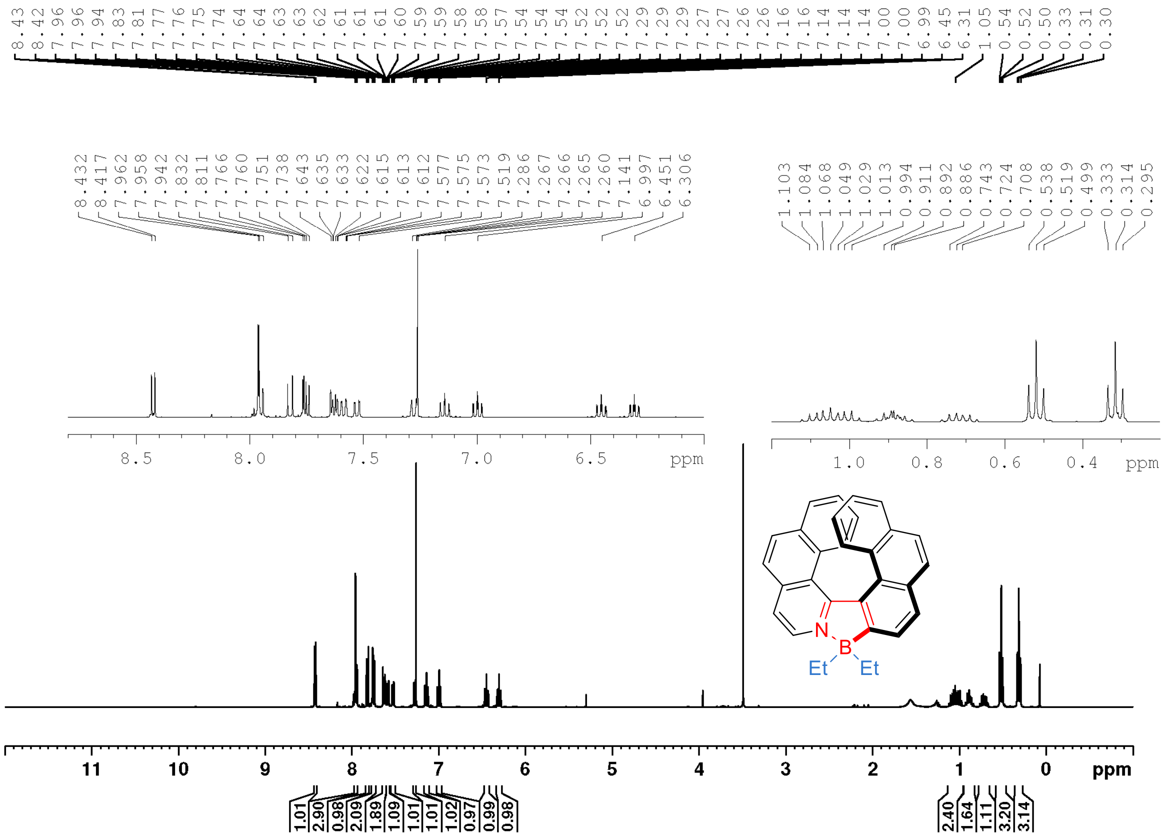

Figure S43. ^{1}H NMR of compound **H1-Et₂** (400 MHz, $CDCl_3$, 25 °C) (method B).

Acquisition Parameter					
Source Type	APCI	Ion Polarity	Positive	Set Nebulizer	2.0 Bar
Focus	Not active	Set Funnel 1 RF	300.0 Vpp	Set Dry Heater	250 °C
Scan Begin	50 m/z	Set Funnel 2 RF	400.0 Vpp	Set Dry Gas	3.0 l/min
Scan End	3000 m/z	Set Hexapole RF	400.0 Vpp	Set Divert Valve	Source

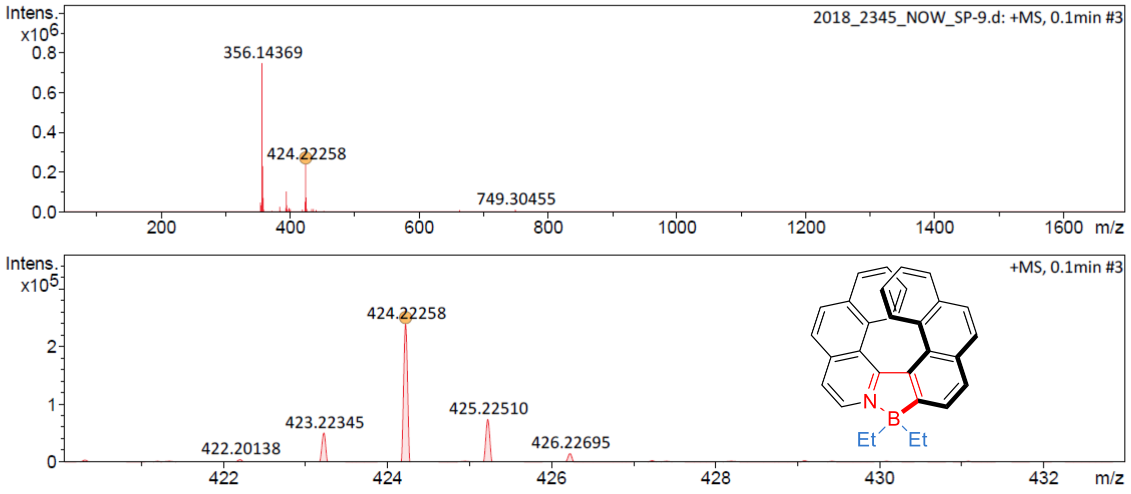

Meas. m/z	#	Ion Formula	m/z	err [ppm]	mSigma	# mSigma	Score	rdb	e⁻ Conf	N-Rule
424.22258	1	C31H27BN	424.22311	2.52	14.7	1	100.00	19.5	even	ok

Figure S44. HRMS (APCI) spectrum of compound **H1-Et₂** (method B).

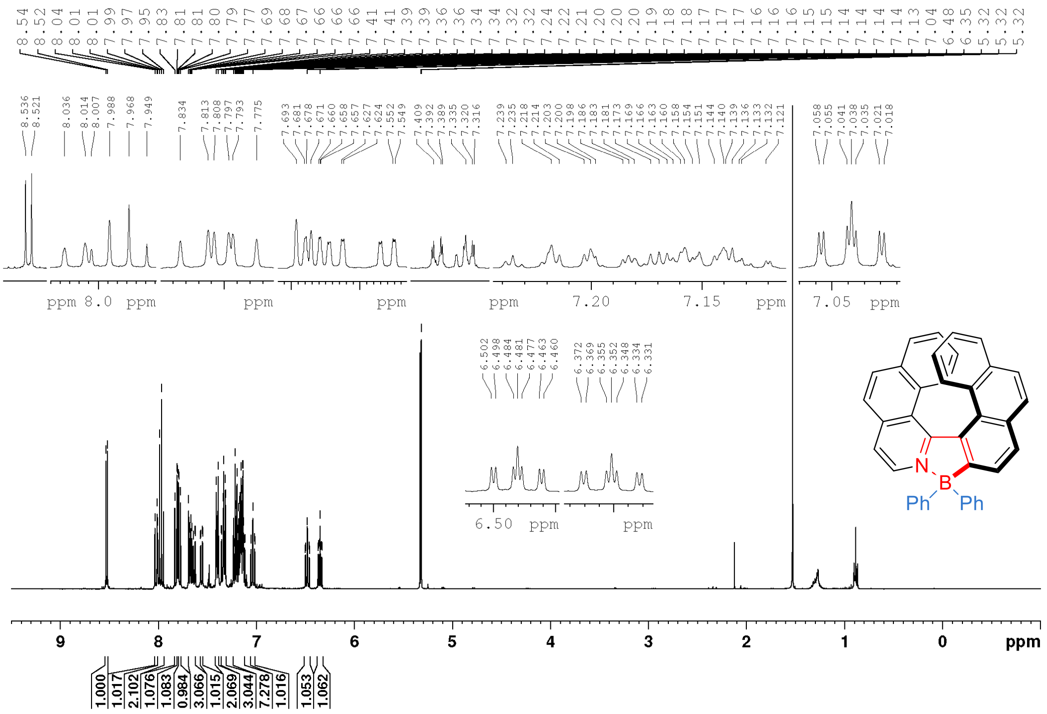

Figure S45. ^1H NMR of compound **H1-Ph$_2$** (400 MHz, CD_2Cl_2, 25 °C).

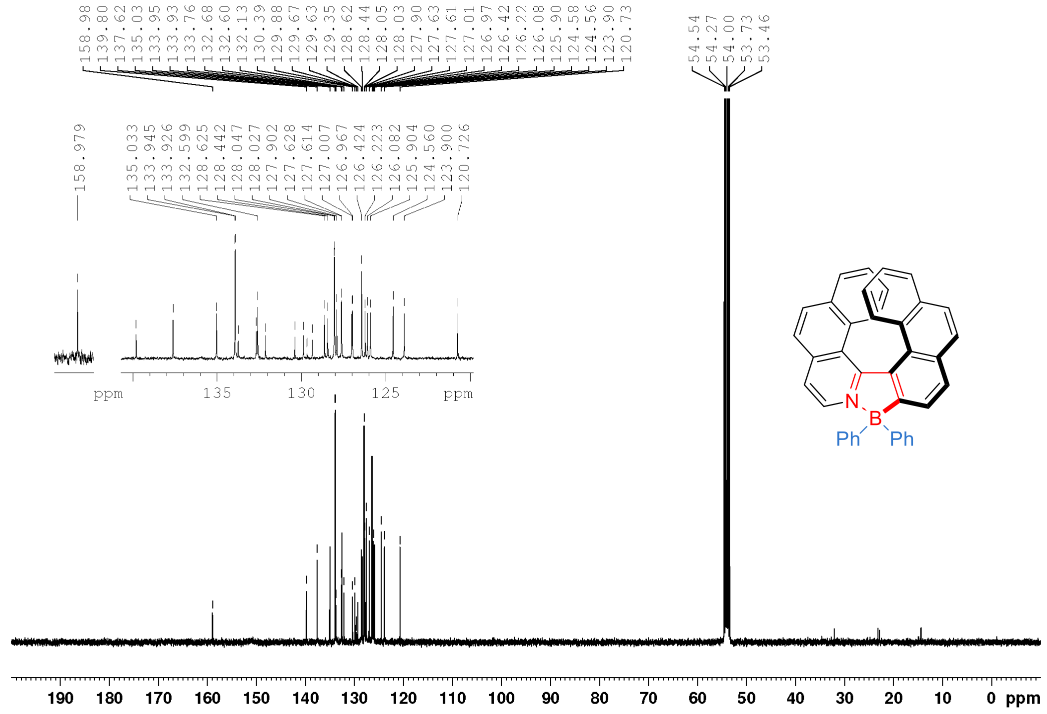

Figure S46. ^{13}C NMR of compound **H1-Ph$_2$** (101 MHz, CD_2Cl_2, 25 °C).

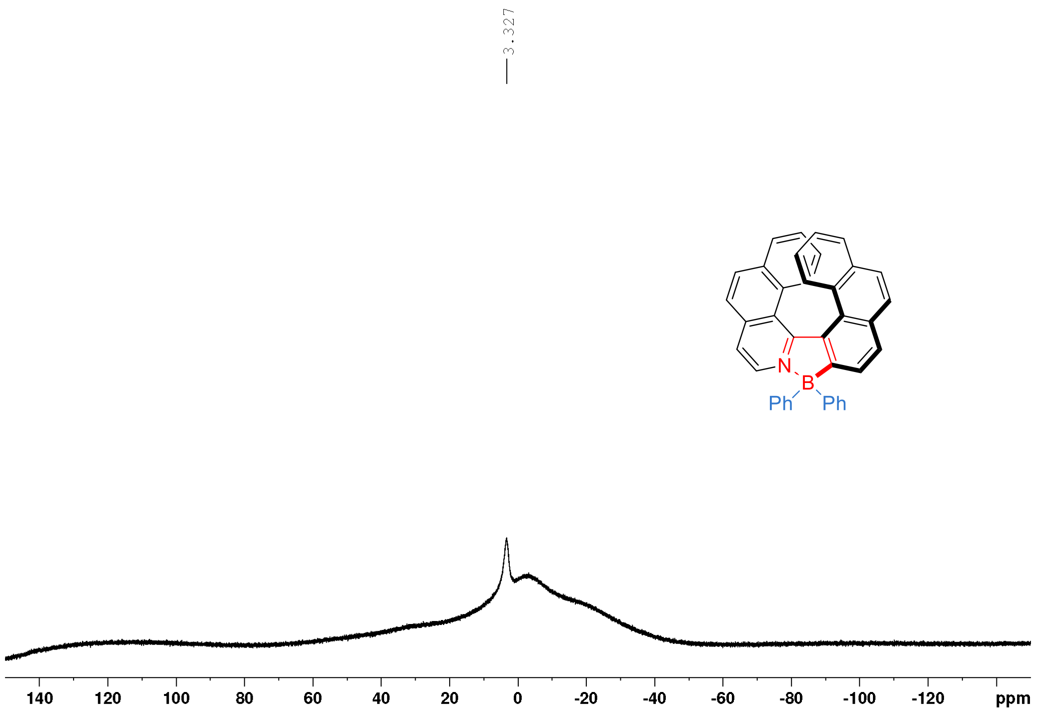

Figure S47. ^{11}B NMR of compound **H1-Ph$_2$** (128 MHz, CD_2Cl_2, 25 °C).

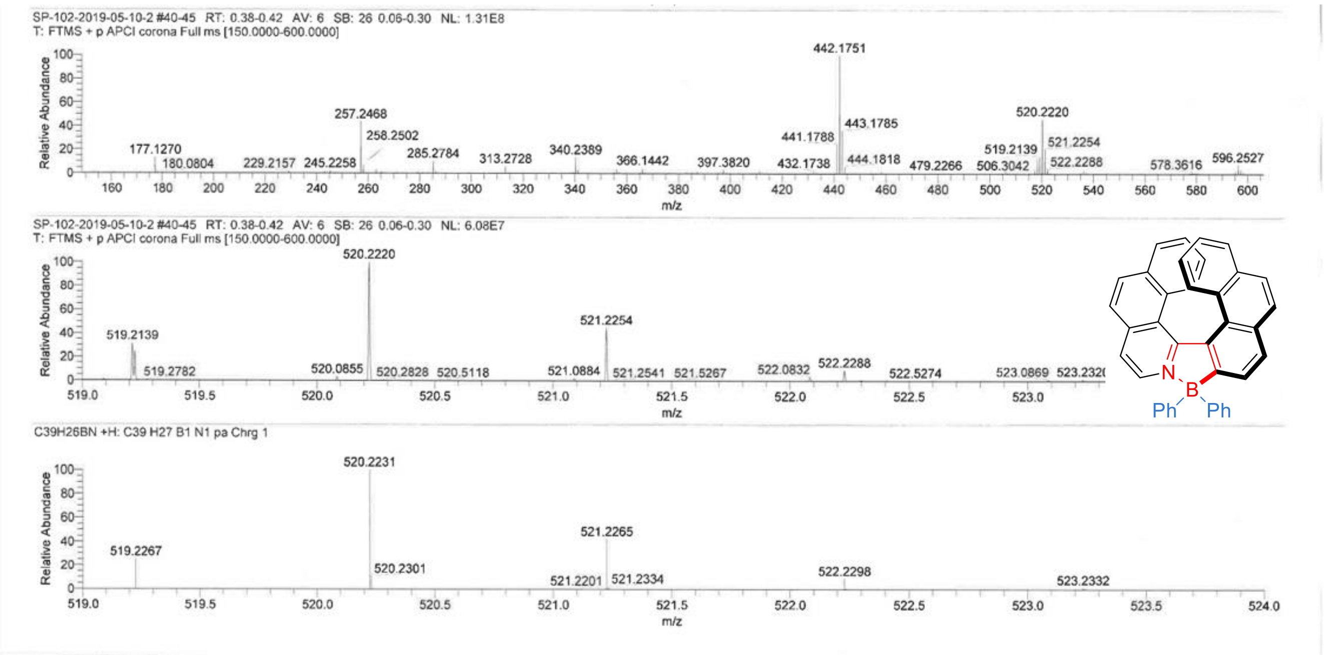

Figure S48. HRMS (APCI) spectrum of compound **H1-Ph$_2$**.

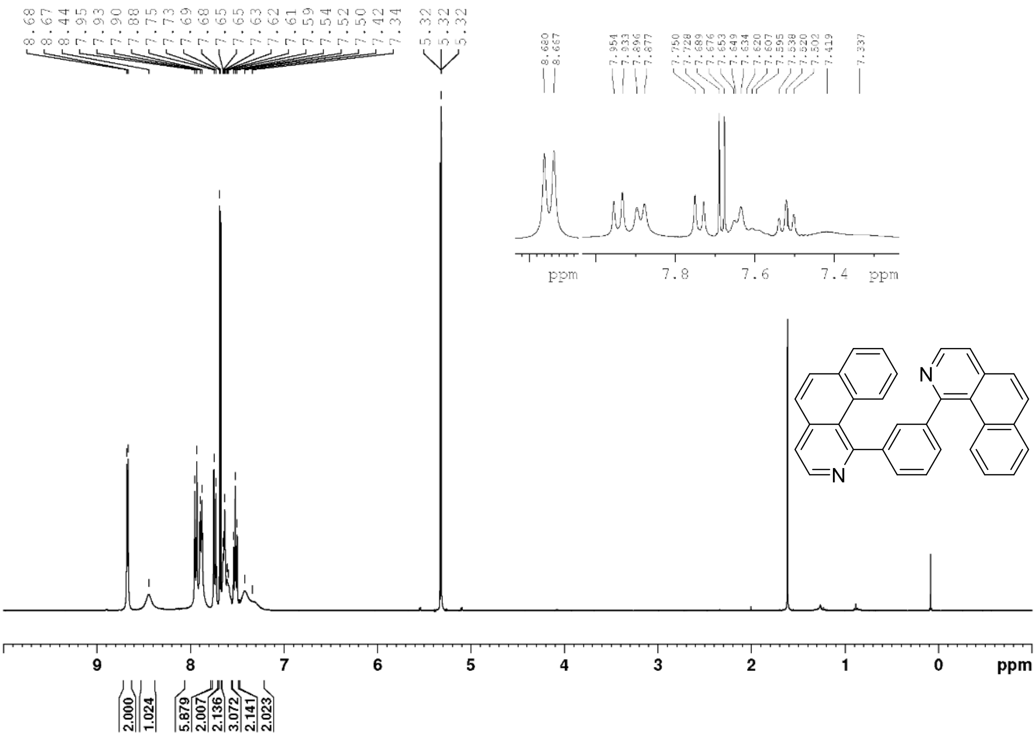

Figure S49. ^{1}H-NMR of compound **TA** (400 MHz, CD_2Cl_2, 25 °C).

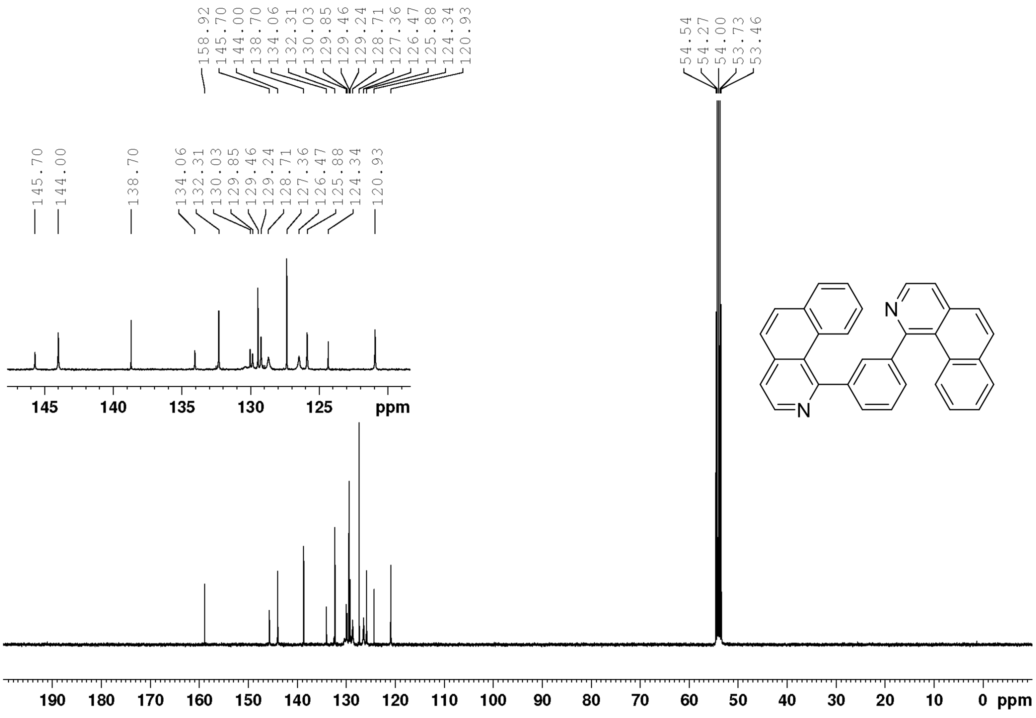

Figure S50. ^{13}C-NMR of compound **TA** (101 MHz, CD_2Cl_2, 25 °C).

Acquisition Parameter

Source Type	ESI	Ion Polarity	Positive	Set Nebulizer	0.3 Bar
Focus	Not active	Set Funnel 1 RF	100.0 Vpp	Set Dry Heater	200 °C
Scan Begin	50 m/z	Set Funnel 2 RF	200.0 Vpp	Set Dry Gas	4.0 l/min
Scan End	2500 m/z	Set Hexapole RF	200.0 Vpp	Set Divert Valve	Source

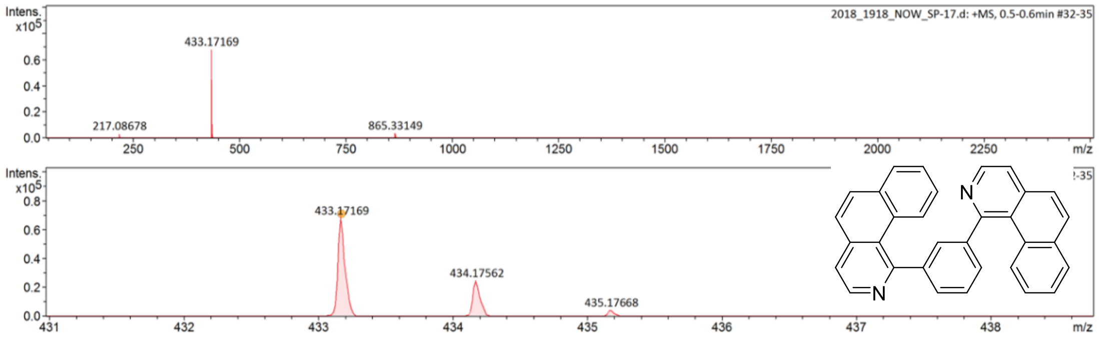

Meas. m/z	#	Ion Formula	m/z	err [ppm]	mSigma	# mSigma	Score	rdb	e⁻ Conf	N-Rule
433.17169	1	C32H21N2	433.17047	-2.81	3.2	1	100.00	23.5	even	ok

Figure S51. HRMS (ESI) spectrum of compound **TA**.

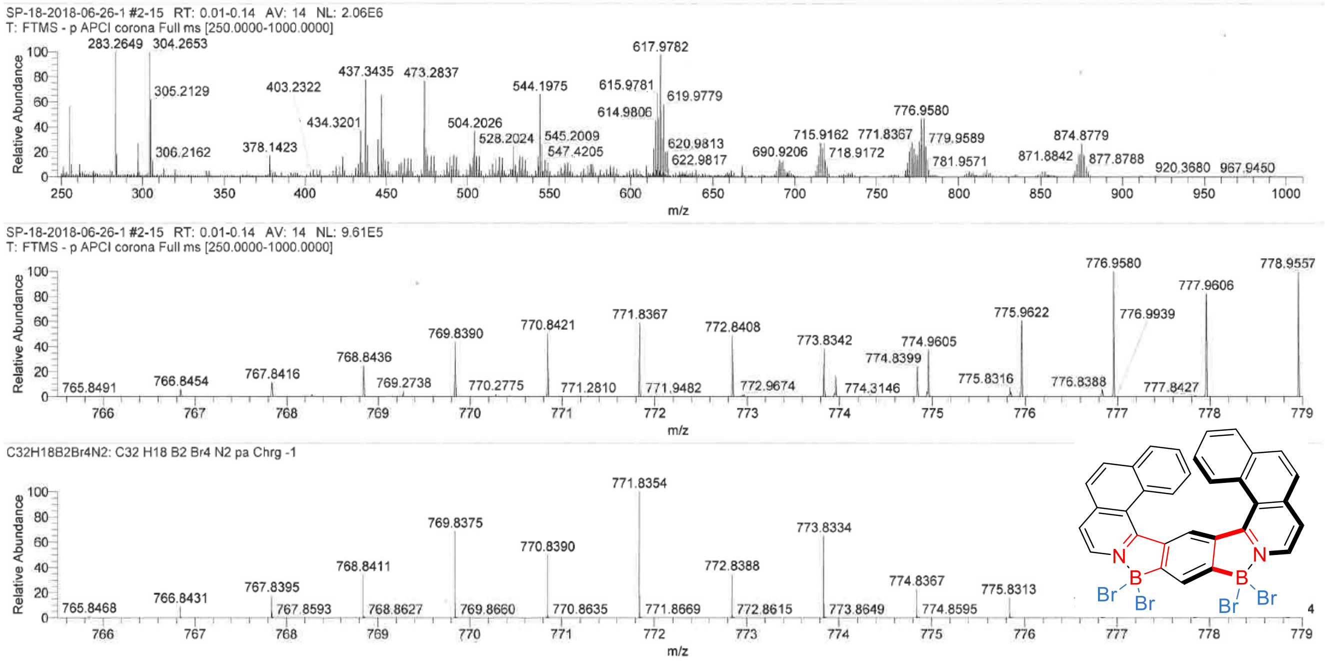

Figure S52. HRMS (APCI) spectrum of compound **H2-Br4**.

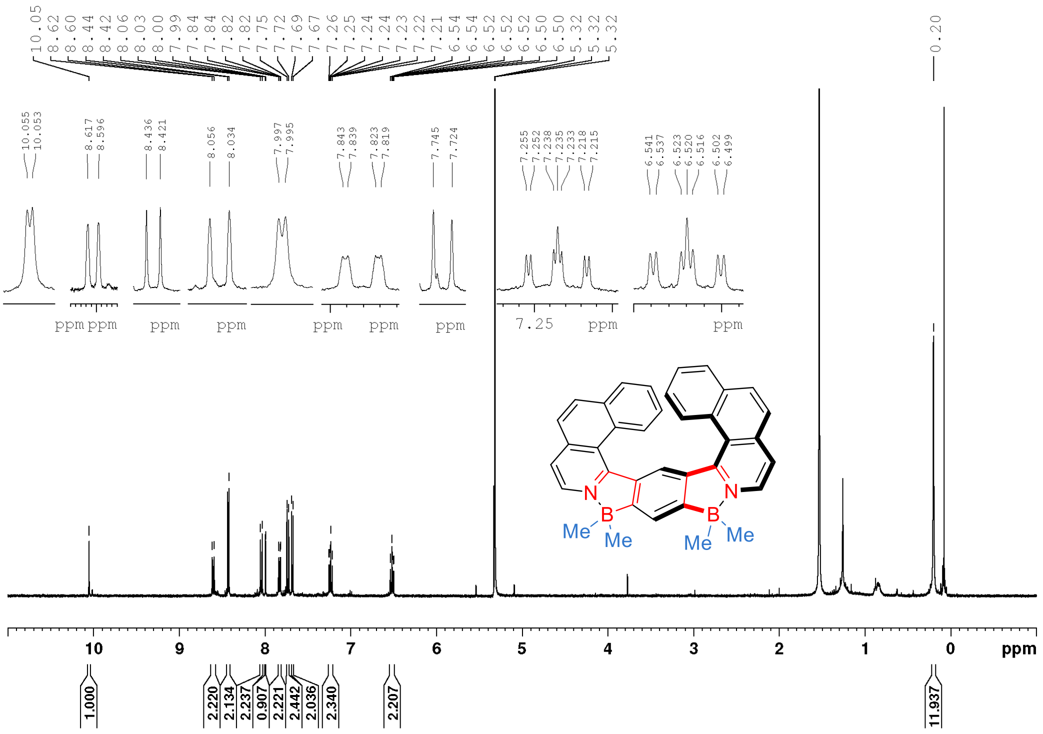

Figure S53. ^{1}H NMR of compound **H2-Me$_4$** (400 MHz, $CDCl_3$, 25 °C).

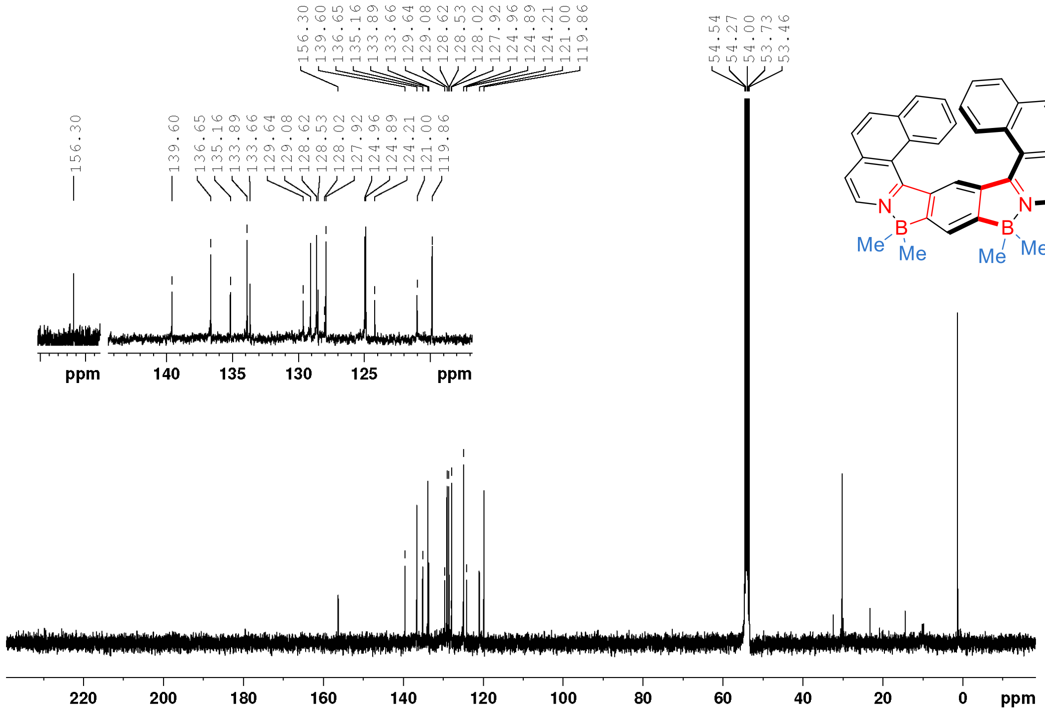

Figure S54. ^{13}C NMR of compound **H2-Me$_4$** (101 MHz, CD_2Cl_2, 25 °C).

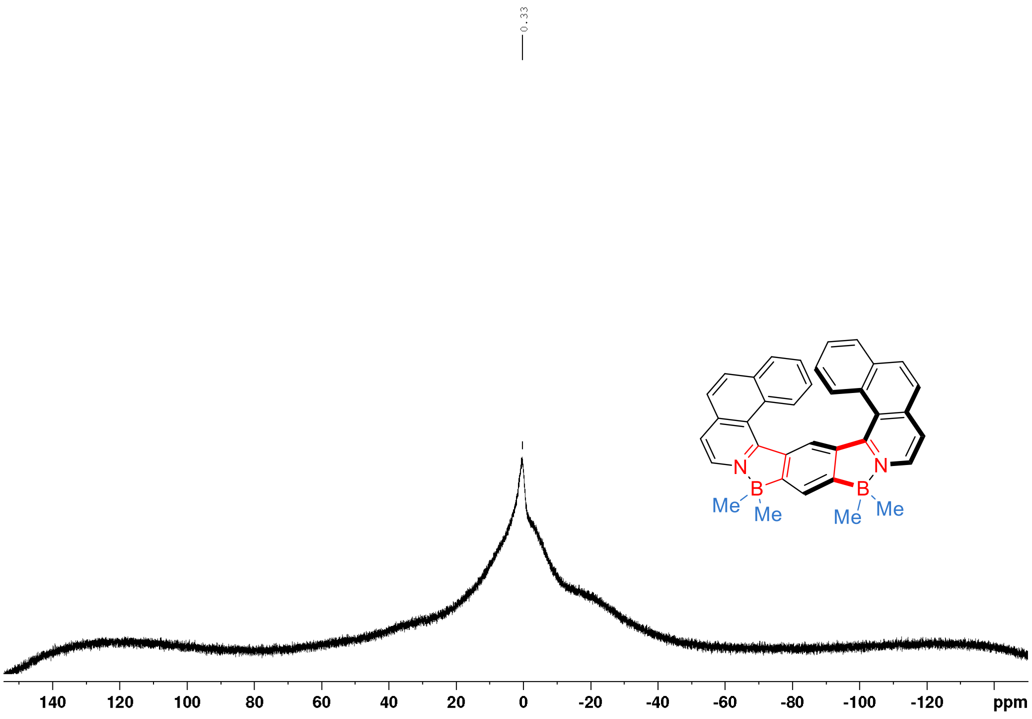

Figure S55. ^{11}B NMR of compound **H2-Me$_4$** (128 MHz, CD_2Cl_2, 25 °C).

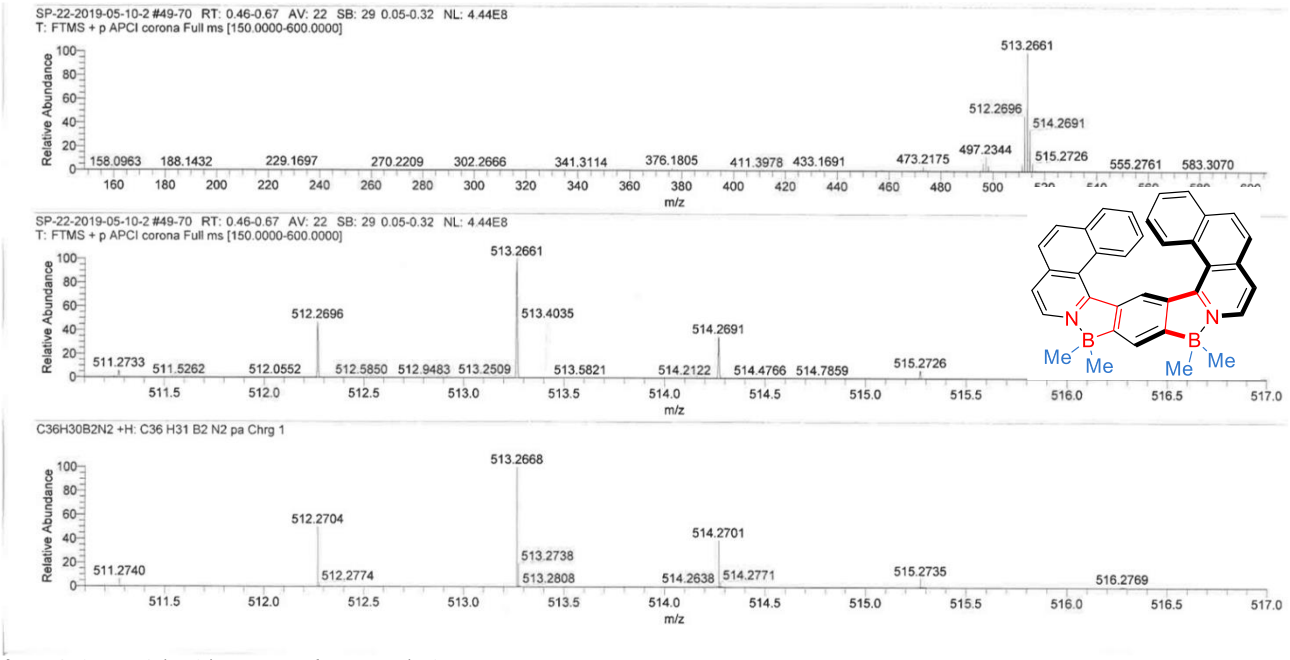

Figure S56. HRMS (APCI) spectrum of compound **H2-Me$_4$**.

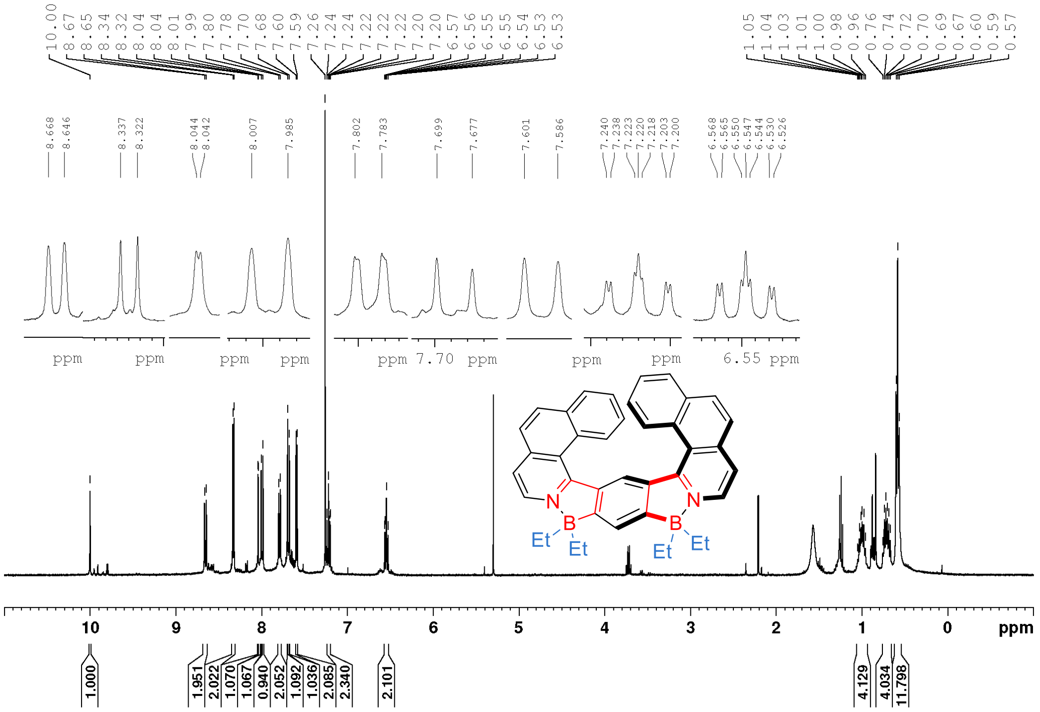

Figure S57. ^{1}H NMR of compound **H2-Et$_4$** (400 MHz, $CDCl_3$, 25 °C).

Acquisition Parameter

Source Type	APCI	Ion Polarity	Positive	Set Nebulizer	2.0 Bar
Focus	Not active	Set Funnel 1 RF	300.0 Vpp	Set Dry Heater	250 °C
Scan Begin	50 m/z	Set Funnel 2 RF	400.0 Vpp	Set Dry Gas	3.0 l/min
Scan End	3000 m/z	Set Hexapole RF	400.0 Vpp	Set Divert Valve	Source

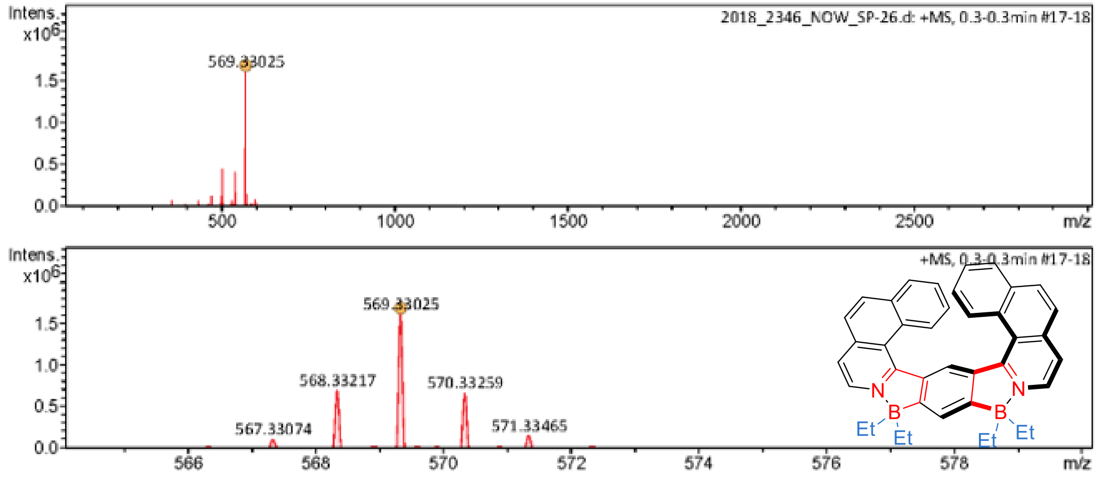

Meas. m/z	#	Ion Formula	m/z	err [ppm]	mSigma	# mSigma	Score	rdb	e⁻ Conf	N-Rule
569.33025	1	C40H39B2N2	569.32939	0.79	4.4	1	100.00	23.5	even	ok

Figure S58. HRMS (ESI) spectrum of compound **H2-Et$_4$**.

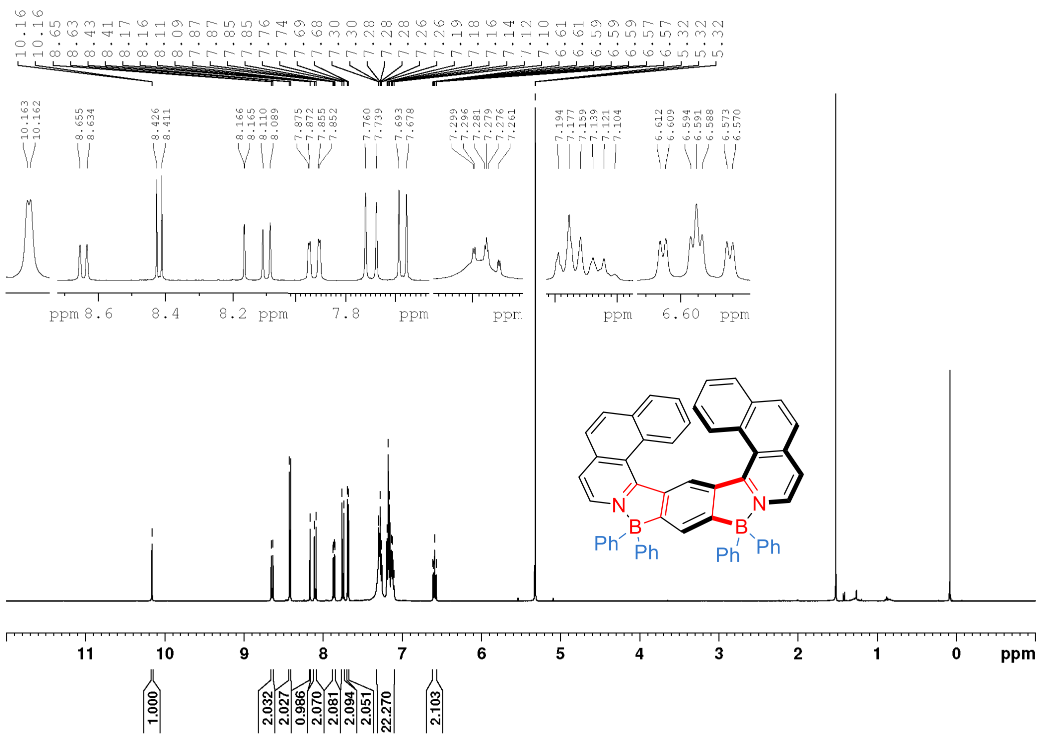

Figure S59. ^{1}H NMR of compound **H2-Ph$_4$** (400 MHz, $CDCl_3$, 25 °C).

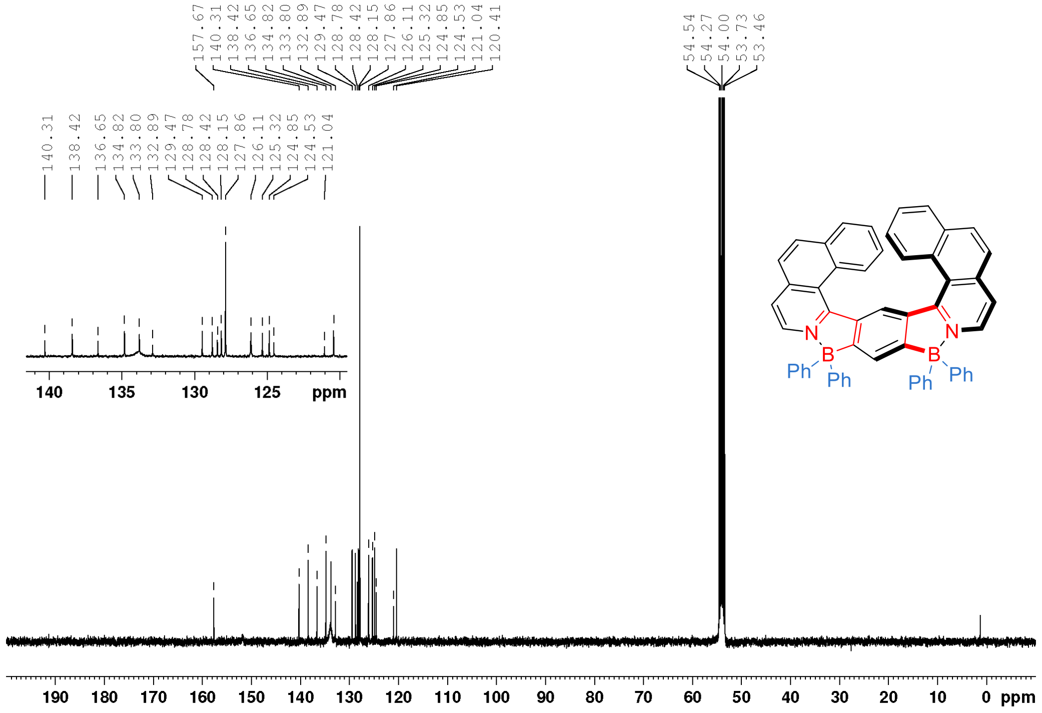

Figure S60. ^{13}C NMR of compound **H2-Ph$_4$** (101 MHz, CD_2Cl_2, 25 °C).

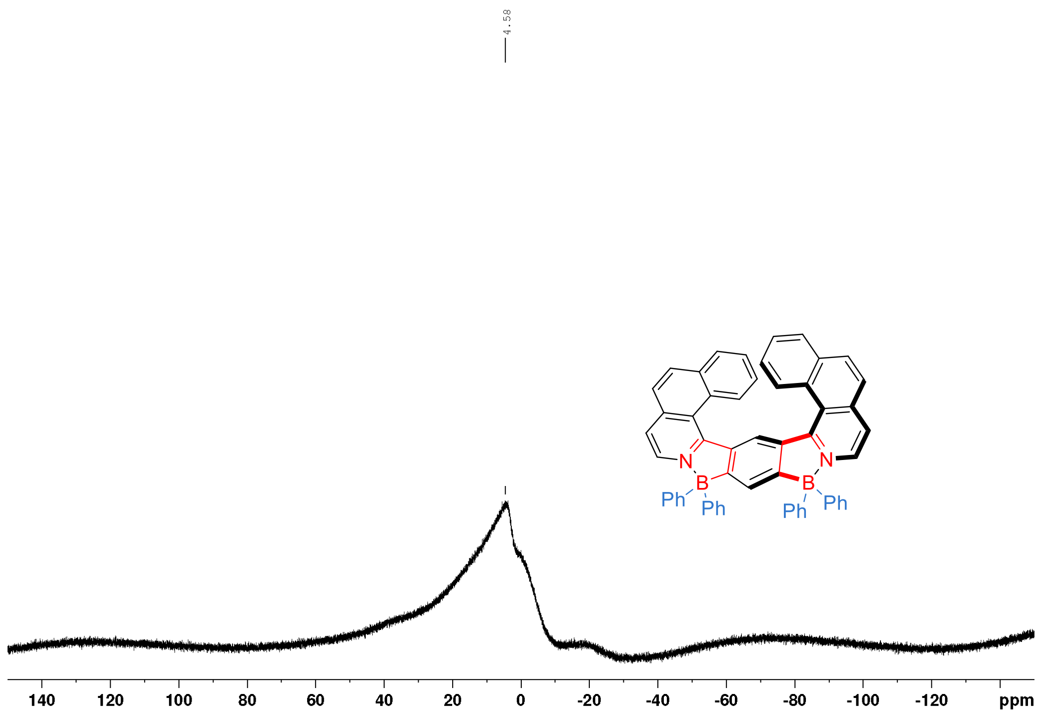

Figure S61. ^{11}B NMR of compound **H2-Ph$_4$** (128 MHz, CD_2Cl_2, 25 °C).

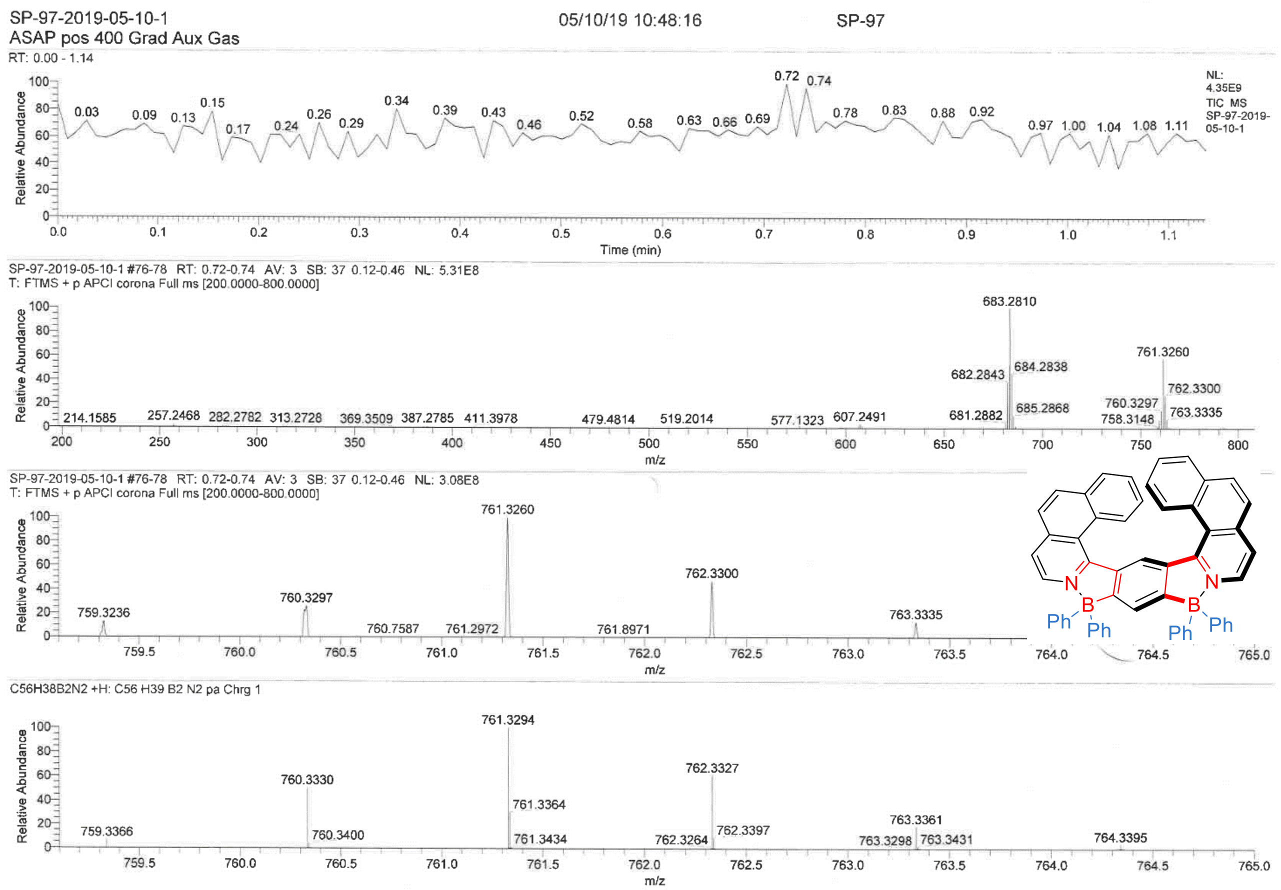

Figure S62. HRMS (ESI) spectrum of compound **H2-Ph$_4$**.

4. Single crystal X-ray analysis

Single crystal X-ray diffraction data for compounds **H1-Me₂**, **H1-Ph₂**, **H2-Me₄**, and **H2-Ph₄** were collected at 100 K on a Bruker D8 Quest Kappa Diffractometer with Photon II CPAD detector and multi-layered mirror monochromated $Cu_K\alpha$ radiation. The structures were solved using direct methods, expanded with Fourier techniques and refined with the Shelx software package.[10] All non-hydrogen atoms were refined anisotropically. Hydrogen atoms were included in the structure factor calculation on geometrically idealized positions.

All the CIF files of crystallographic data have been deposited with the Cambridge Crystallographic Data Centre under the CCDC numbers 1942991, 1942993, 1942995 and 1942994, respectively. These data can be obtained free of charge from The Cambridge Crystallographic Data Centre via www.ccdc.ac.uk/data.request/cif.

Crystal data for H1-Me$_2$: $C_{29}H_{22}BN$, *Mr* = 395.28, yellow block, 0.347×0.272×0.051 mm^3, monoclinic space group P2$_1$/*n*, *a* = 12.3755(6) Å, *b* = 11.0420(5) Å, *c* = 15.4876(8) Å, α = 90°, β = 99.498(2)°, γ = 90°, V = 2087.37(18) Å^3 , *Z* = 4, ρ_{calcd} = 1.258 g·cm^{-3}, μ = 0.543 mm^{-1}, $F_{(000)}$ = 832, T = 100(2) K, *GooF*(F^2) = 1.031, R_1 = 0.0393, wR^2 = 0.1007 for I>2σ(I), R_1 = 0.0428, wR^2 = 0.1037 for all data, 4123 unique reflections [$\theta \leq$ 72.294°] with a completeness of 99.7% and 282 parameters, 0 restraints. CCDC 1942991.

Crystal data for H1-Ph$_2$: $C_{39}H_{26}BN$, *Mr* = 519.42, yellow block, 0.121×0.095×0.043 mm^3, triclinic space group P$\bar{1}$, *a* = 10.1360(3) Å, *b* = 11.2403(4) Å, *c* = 12.8449(4) Å, α = 69.302(2)°, β = 82.133(2)°, γ = 86.657(2)°, V = 1356.02(8) Å^3, *Z* = 2, ρ_{calcd} = 1.272 g·cm^{-3}, μ = 0.551 mm^{-1}, $F_{(000)}$ = 544, T = 100(2) K, *GooF*(F^2) = 1.054, R_1 = 0.0488, wR^2 = 0.1055 for I>2σ(I), R_1 = 0.0671, wR^2 = 0.1174 for all data, 5331 independent reflections [$\theta \leq$ 72.221°] with a completeness of 99.6% and 371 parameters, 0 restraints. CCDC 1942993.

Crystal data for H2-Me$_4$: $C_{36}H_{30}B_2N_2$, *Mr* = 512.24, colorless plate, 0.172×0.050×0.014 mm^3, monoclinic space group P2$_1$/*n*, *a* = 13.8040(8) Å, *b* = 11.9514(7) Å, *c* = 16.7181(10) Å, α = 90°, β = 91.729(4)°, γ = 90°, V = 2756.8(3) Å^3, *Z* = 4, ρ_{calcd} = 1.234 g·cm^{-3}, μ = 0.535 mm^{-1} , $F_{(000)}$ = 1080, T = 100(2) K, *GooF*(F^2) = 1.027, R_1 = 0.0684, wR^2 = 0.1742 for I>2σ(I), R_1 = 0.1040, wR^2 = 0.2049 for all data, 5470 independent reflections [$\theta \leq$ 73.051°] with a completeness of 99.0% and 365 parameters, 0 restraints. CCDC 1942995.

Crystal data for H2-Ph$_4$: $C_{56}H_{38}B_2N_2$,CH_2Cl_2, *Mr* = 845.43, colorless block, 0.257×0.101×0.040 mm^3, triclinic space group P$\bar{1}$, *a* = 12.3206(11) Å, *b* = 13.3049(11) Å, *c* = 14.8111(13) Å, α = 102.329(4)°, β = 113.494(4)°, γ = 90.296(4)°, V = 2164.2(3) Å^3, *Z* = 2, ρ_{calcd} = 1.297 g·cm^{-3}, μ = 1.669 mm^{-1}, $F_{(000)}$ = 880, T = 100(2) K, *GooF*(F^2) = 1.019, R_1 = 0.0709, wR_2 = 0.1652 for I>2σ(I), R_1 = 0.0996, wR^2 = 0.1908 for all data, 8445 independent reflections [$\theta \leq$ 72.342°] with a completeness of 98.6% and 590 parameters, 11 restraints. CCDC 1942994.

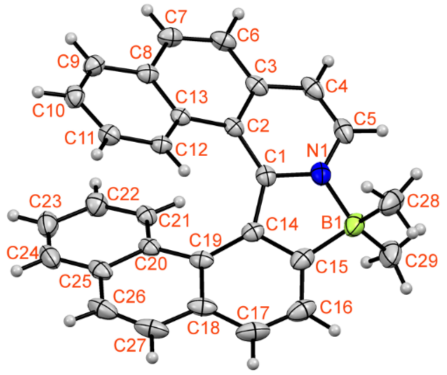

Angles

- ∢ (C15-B1-C28) 112.8(1)
- ∢ (C15-B1-C29) 114.4(1)
- ∢ (C15-B1-N1) 96.1(1)
- ∢ (C28-B1-C29) 113.7(1)
- ∢ (C28-B1-N1) 107.7(1)
- ∢ (C29-B1-N1) 110.5(1)

Bond Lengths / Å

B1-C15 1.589(2)	C11-C12 1.375(2)
B1-C28 1.629(2)	C12-C13 1.408(1)
B1-C29 1.621(2)	C14-C15 1.404(2)
B1-N1 1.607(2)	C14-C19 1.431(2)
C1-C2 1.430(2)	C15-C16 1.400(2)
C1-C14 1.490(2)	C16-C17 1.367(2)
C1-N1 1.362(2)	C17-C18 1.416(2)
C2-C13 1.453(2)	C18-C27 1.433(2)
C3-C4 1.409(2)	C19-C20 1.454(2)
C3-C6 1.435(2)	C20-C21 1.410(2)
C4-C5 1.361(2)	C21-C22 1.376(2)
C5-N1 1.352(2)	C22-C23 1.404(2)
C6-C7 1.347(2)	C23-C24 1.367(2)
C7-C8 1.433(2)	C24-C25 1.409(2)
C8-C9 1.409(2)	C25-C26 1.431(2)
C9-C10 1.372(2)	C26-C27 1.347(2)
C10-C11 1.404(2)	

Figure S63. Molecular structure of **H1-Me$_2$** in the solid state with the selected bond lengths and angles. Thermal displacement parameters are displayed at the 50% probability.

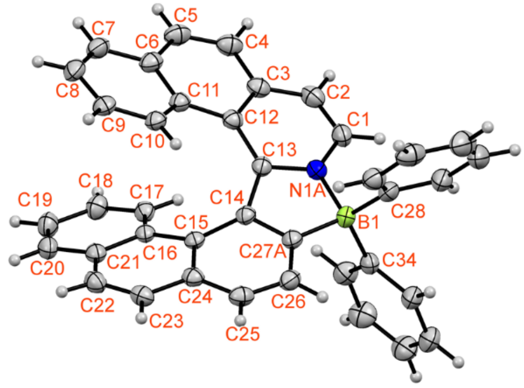

Angles

∢ (C28-B1-C34) 115.8(2)°
∢ (C28-B1-N1A) 110.5(2)°
∢ (C28-B1-C27A) 113.2(2)°
∢ (C34-B1-N1A) 111.1(2)°
∢ (C34-B1-C27A) 108.6(2)°
∢ (N1A-B1-C27A) 95.9(1)°

Bond Lengths / Å

B1-C28 1.618(3)	C13-C14 1.488(2)
B1-C34 1.629(2)	C13-N1A 1.381(2)
B1-N1A 1.597(3)	C14-C15 1.433(3)
B1-C27A 1.595(3)	C14-C27A 1.383(3)
C1-C2 1.356(3)	C15-C16 1.454(3)
C1-N1A 1.377(2)	C16-C17 1.406(3)
C2-C3 1.403(3)	C17-C18 1.382(3)
C3-C4 1.429(3)	C18-C19 1.403(3)
C4-C5 1.346(3)	C19-C20 1.367(3)
C5-C6 1.425(3)	C20-C21 1.409(3)
C6-C7 1.407(3)	C21-C22 1.430(3)
C7-C8 1.360(3)	C22-C23 1.349(3)
C8-C9 1.409(3)	C23-C24 1.433(3)
C9-C10 1.376(3)	C24-C25 1.406(3)
C10-C11 1.407(2)	C25-C26 1.361(3)
C11-C12 1.456(2)	C26-C27A 1.375(2)
C12-C13 1.439(2)	

Figure S64. Molecular structure of **H1-Ph$_2$** in the solid state with the selected bond lengths and angles. Thermal displacement parameters are displayed at the 50% probability.

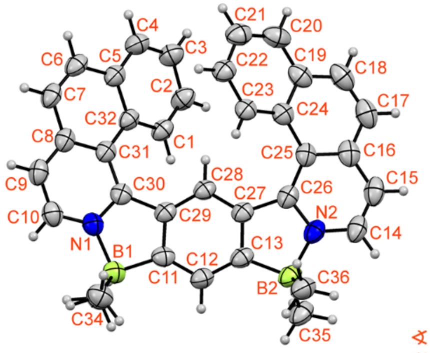

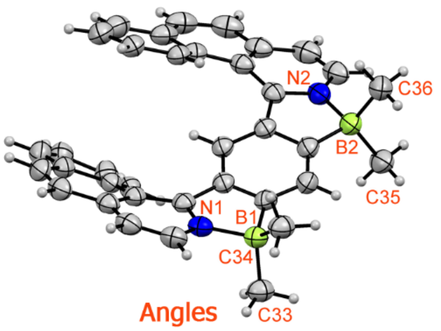

Bond Lengths / Å

B1-C11 1.594(4)	C13-C27 1.418(4)
B1-C33 1.626(4)	C14-C15 1.352(4)
B1-C34 1.618(4)	C14-N2 1.350(4)
B1-N1 1.626(4)	C15-C16 1.412(4)
B2-C13 1.598(5)	C16-C17 1.426(4)
B2-C35 1.628(4)	C17-C18 1.357(5)
B2-C36 1.621(5)	C18-C19 1.430(4)
B2-N2 1.616(5)	C19-C20 1.411(4)
C1-C2 1.365(4)	C20-C21 1.370(4)
C1-C32 1.414(4)	C21-C22 1.398(4)
C2-C3 1.402(4)	C22-C23 1.377(4)
C3-C4 1.370(4)	C23-C24 1.415(4)
C4-C5 1.405(4)	C24-C25 1.461(4)
C5-C6 1.428(4)	C25-C26 1.428(4)
C6-C7 1.345(4)	C26-C27 1.481(4)
C7-C8 1.435(4)	C26-N2 1.352(3)
C8-C9 1.407(4)	C27-C28 1.395(4)
C9-C10 1.359(4)	C28-C29 1.393(4)
C10-N1 1.348(3)	C29-C30 1.479(4)
C11-C12 1.390(4)	C30-C31 1.427(4)
C11-C29 1.415(4)	C30-N1 1.349(3)
C12-C13 1.389(4)	C31-C32 1.444(4)

Angles

∢ (C11-B1-C33) 112.4(2)°	∢ (C13-B2-C35) 113.1(3)°
∢ (C11-B1-C34) 115.6(2)°	∢ (C13-B2-C36) 113.4(3)°
∢ (C11-B1-N1) 95.8(2)°	∢ (C13-B2-N2) 96.4(2)°
∢ (C33-B1-C34) 114.6(2)°	∢ (C35-B2-C36) 114.4(3)°
∢ (C33-B1-N1) 106.5(2)°	∢ (C35-B2-N2) 109.8(3)°
∢ (C34-B1-N1) 109.8(2)°	∢ (C36-B2-N2) 108.0(3)°

Figure S65. Molecular structure of **H2-Me$_4$** in the solid state with the selected bond lengths and angles. Thermal displacement parameters are displayed at the 50% probability.

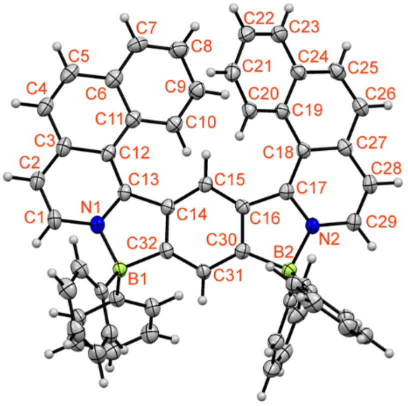

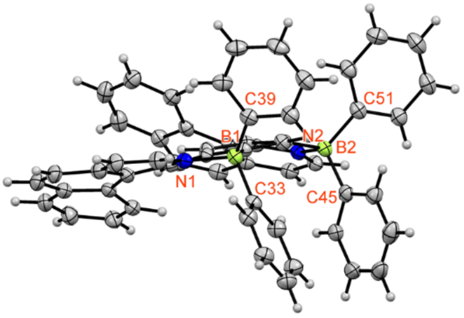

Bond Lengths / Å

B1-N1 1.609(5)	C11-C12 1.453(4)
B1-C32 1.590(5)	C12-C13 1.432(4)
B1-C33 1.636(5)	C13-C14 1.484(4)
B1-C39 1.616(4)	C14-C15 1.397(5)
B2-N2 1.612(6)	C14-C32 1.415(5)
B2-C30 1.602(4)	C15-C16 1.399(4)
B2-C45 1.614(5)	C16-C17 1.478(4)
B2-C51 1.620(4)	C16-C30 1.417(5)
N1-C1 1.363(4)	C17-C18 1.418(5)
N1-C13 1.356(4)	C18-C19 1.456(5)
N2-C17 1.358(4)	C19-C20 1.414(3)
N2-C29 1.356(3)	C20-C21 1.374(5)
C1-C2 1.358(6)	C21-C22 1.410(7)
C2-C3 1.404(6)	C22-C23 1.368(5)
C3-C4 1.425(6)	C23-C24 1.412(6)
C4-C5 1.351(6)	C24-C25 1.437(3)
C5-C6 1.432(4)	C25-C26 1.348(4)
C6-C7 1.406(6)	C26-C27 1.440(5)
C7-C8 1.368(5)	C27-C28 1.408(4)
C8-C9 1.402(6)	C28-C29 1.361(5)
C9-C10 1.381(5)	C30-C31 1.389(5)
C10-C11 1.410(3)	C31-C32 1.393(4)

Angles

∢ (N1-B1-C32) 96.0(2)°	∢ (N2-B2-C30) 95.9(2)°
∢ (N1-B1-C33) 108.0(2)°	∢ (N2-B2-C45) 110.7(3)°
∢ (N1-B1-C39) 112.4(3)°	∢ (N2-B2-C51) 108.6(3)°
∢ (C32-B1-C33) 110.7(3)°	∢ (C30-B2-C45) 107.9(3)°
∢ (C32-B1-C39) 114.0(3)°	∢ (C30-B2-C51) 118.0(3)°
∢ (C33-B1-C39) 114.3(3)°	∢ (C45-B2-C51) 114.2(3)°

Figure S66. Molecular structure of **H2-Ph$_4$** in the solid state with the selected bond lengths and angles. Thermal displacement parameters are displayed at the 50% probability.

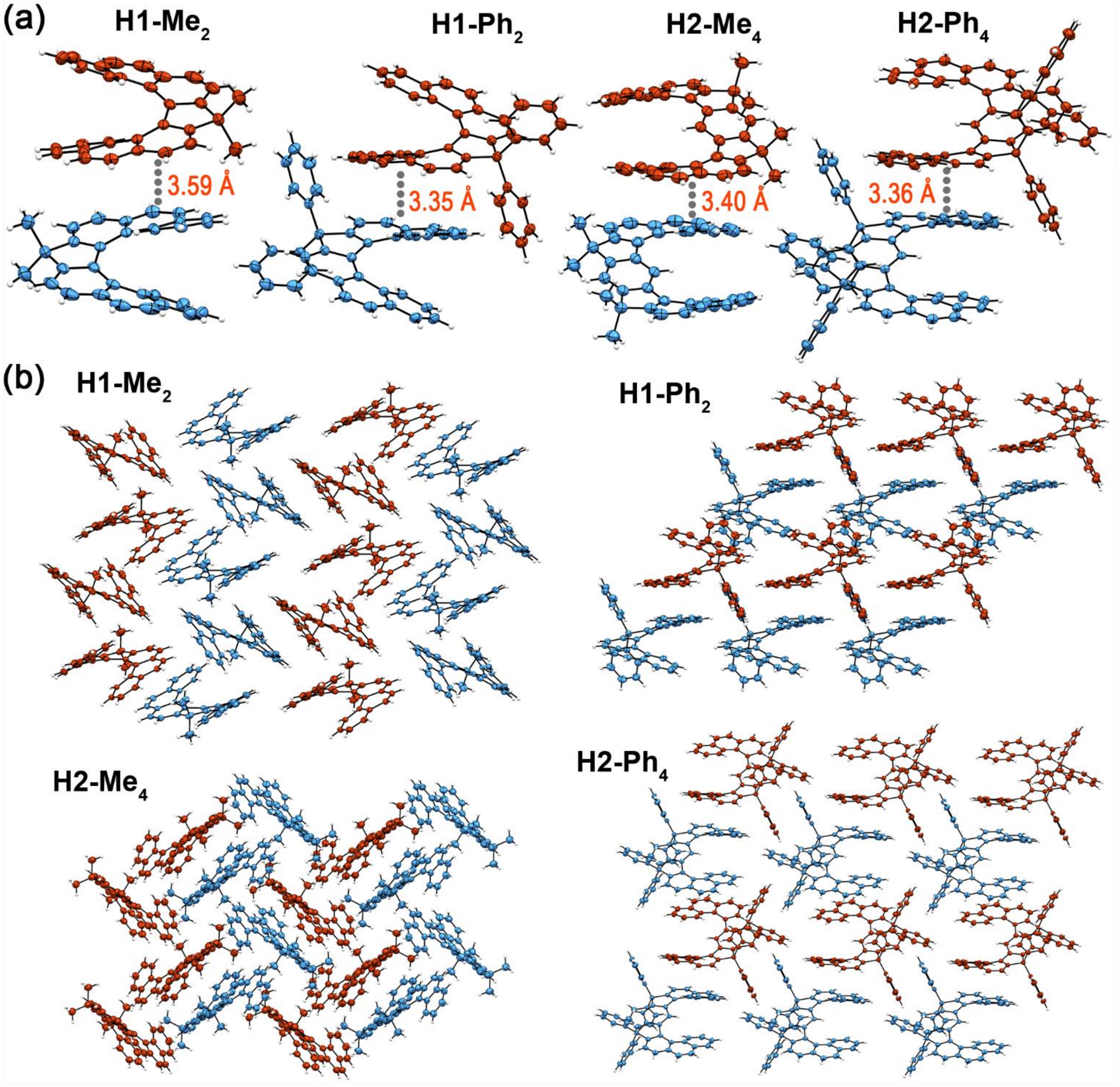

Figure S67. a) Dimers and b) packing arrangements of **H1-Me$_2$**, **H1-Ph$_2$**, **H2-Me$_4$**, and **H2-Ph$_4$**. Enantiomers with *P*- and *M*-helicity are colored in maroon and blue, respectively. The ORTEP drawings are shown with 50% probability. Co-crystallized solvent molecules, i.e. CH_2Cl_2, in the crystal strcture of **H2-Ph$_4$** are omitted for clarity.

5. Optical and Electrochemical Properties

Table S1. Optical and electrochemical properties of helical compounds.

Cpd	λ_{abs}[a] / nm	ε_{max}[b] / 10^3 M^{-1} cm^{-1}	λ_{em}[c] / nm	Stokes shift / cm^{-1}	Φ_{fl}[d]	λ_{abs}[e] / nm	λ_{em}[f] / nm	Φ_{fl}[g]	$E_{1/2}$ (red1)[h] / V	$E_{1/2}$ (red2)[h] / V	$E_{1/2}$ (ox1)[h] / V	$E_{1/2}$ (ox1)[h] / V
**H1-Me₂**	426<br>407	9.7<br>8.7	459	1700	0.20	433<br>412	504	0.17	–2.11 (–2.11)	---	+0.93[i]<br>(+0.89)	---
**H1-Et₂**	427<br>408	7.6<br>6.9	462	1800	0.18	433<br>413	508	0.03	–2.13 (–2.13)	---	+0.90[i]<br>(+0.88)	+1.57[i]<br>(+1.52)
**H1-Ph₂**	432<br>412	8.1<br>8.0	477	2200	0.24	438<br>418	555	0.23	–1.99 (–1.99)	---	+1.09[i]<br>(+1.01)	---
**H2-Me₄**	403	12.6	443	2250	0.43	413	465	0.08	(–2.24)	---	+0.86[i]<br>(+0.82)	+1.22[i]<br>(+1.17)
**H2-Ph₄**	406	19.7	446	2200	0.47	412<br>401	459	0.25	–2.09 (–2.09)	(–2.31)	+1.08[i]<br>(+1.14)	---

[a] Absorption maximum in CH_2Cl_2 solution. [b] Molar absorption coefficient. [c] Fluorescence maximum in CH_2Cl_2 solution. [d] Relative fluorescence quantum yields determined by the optical dilution method. [e] Absorption maximum in the thin film [f] Fluorescence maximum for a powder sample. [g] Effective quantum yield for powders without correction for reabsorption; Φ_{fl} error < 2%. [h] Redox potentials were measured in dry CH_2Cl_2 (c ~ 10^{-4} – 10^{-5} M) at a scan rate of 100 mV s^{-1} and with 50% of iR compensation; supporting electrolyte Bu_4NPF_6 (c = 0.1 M). Measurements were calibrated vs. the ferrocenium/ferrocene (Fc^+/Fc) redox couple as an internal standard. The values in parentheses correspond do DPV and SW measurements. [i] Peak potential.

Absorption and Emission in the Solid State

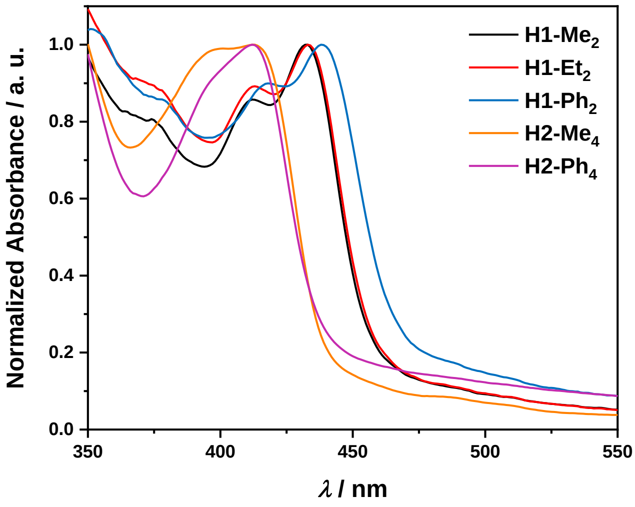

Figure S68. Absorption spectra of **H1-Me₂** (black line), **H1-Et₂** (red line), **H1-Ph₂** (blue line), **H2-Me₄** (orange line), and **H2-Ph₄** (purple line) in spin-coated thin films (from $CHCl_3$ for **H1-Me₂**, **H1-Et₂**, **H2-Ph₄** and CH_2Cl_2 for **H1-Ph₂**, **H2-Me₄**, 9.8 × 10^{-3} - 1.9 × 10^{-2} M, 2000 rpm, 60 sec) on quartz substrates normalized to the 0-0 vibronic progression.

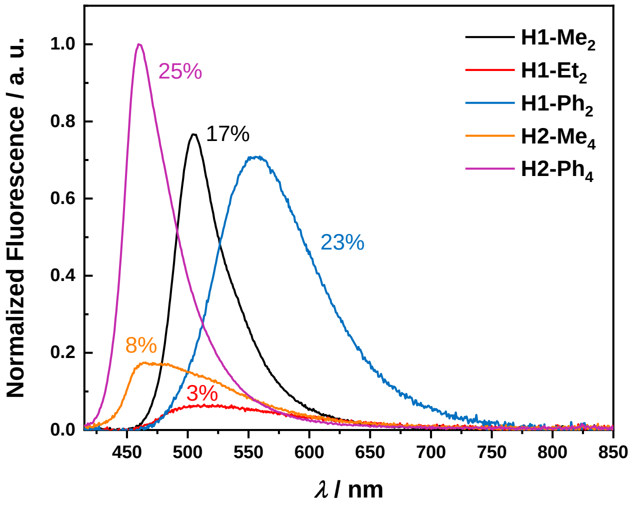

Figure S69. Fluorescence spectra of **H1-Me₂** (black line), **H1-Et₂** (red line), **H1-Ph₂** (blue line), **H2-Me₄** (orange line), and **H2-Ph₄** (purple line) of amorphous powders upon excitation at λ_{ex} of 405 nm for **H1-Me₂** and **H1-Et₂**, 410 nm for **H1-Ph₂**, and 380 nm for **H2-Me₄** and **H2-Ph₄**. The spectra are scaled to their absolute quantum yields determined by an integrating sphere setup.

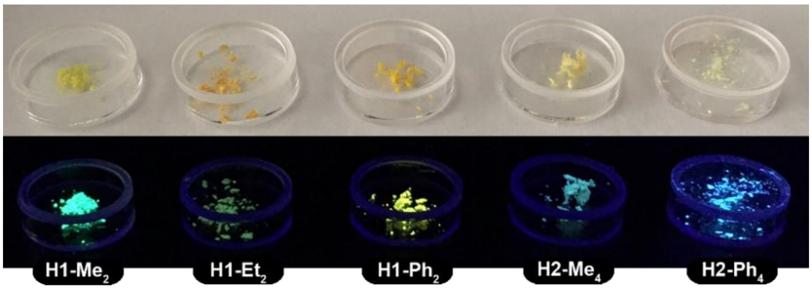

Figure S70. Photographs of the powders of **H1** and **H2** upon ambient (top) and UV (bottom) irradiation.

Cyclic Voltammetry and Pulse Technique

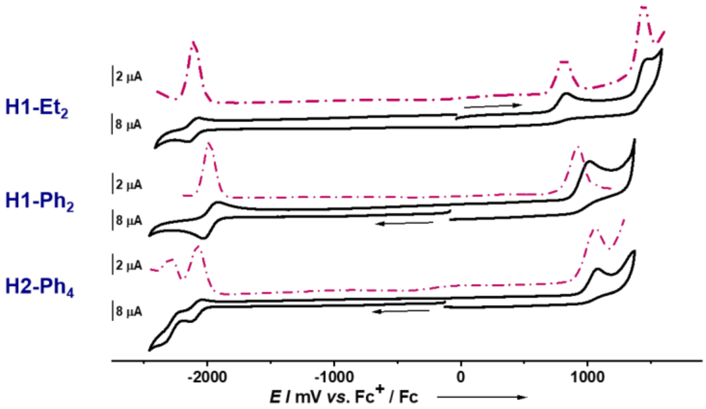

Figure S71. Cyclic (solid lines) and differential pulse or square-wave (dashed lines) voltammograms of **H1-Et₂**, **H1-Ph₂**, and **H2-Ph₄** in CH_2Cl_2 with Bu_4NPF_6 as a supporting electrolyte and calibrated versus the ferrocenium/ferrocene (Fc^+/Fc) redox couple as an internal standard.

6. Resolution of Enantiomers

Analytical HPLC

Chiral resolution of racemates of **H1-Me₂**, **H1-Et₂** and **H1-Ph₂** into their corresponding enantiomers was performed by HPLC using columns with a chiral stationary phase from Dr. Maisch GmbH (ReproSil Chiral-MIF).

Samples of helicenes **(*rac*)-H1-Me$_2$** and **(*rac*)-H1-Et$_2$** were dissolved in *n*-hexane/CH_2Cl_2 (70:30), injected on a column with a chiral stationary phase (ReproSil Chiral-MIF, 250 × 4.6 mm) and eluted with *n*-hexane/CH_2Cl_2 (93:7) at room temperature with a flow rate of 1 mL min^{-1}. A sample of **(*rac*)-H1-Ph$_2$** was dissolved in *n*-hexane/CH_2Cl_2 (70:30), injected on a column with a chiral stationary phase (ReproSil Chiral-MIF, 250 × 4.6 mm) and eluted in *n*-hexane/CH_2Cl_2 (85:15) at room temperature with a flow rate of 1 mL min^{-1}.

Semipreparative HPLC

Samples of helicenes **(*rac*)-H1-Me$_2$** and **(*rac*)-H1-Et$_2$** were dissolved in *n*-hexane/CH_2Cl_2 (85:15), injected on a column (0.83 mg mL^{-1}) with a chiral stationary phase (ReproSil Chiral-MIF, 250 × 10 mm) and eluted with *n*-hexane/CH_2Cl_2 (85:15). A sample of helicene **(*rac*)-H1-Ph$_2$** was dissolved in *n*-hexane/CH_2Cl_2 (70:30), injected on a column with a chiral stationary phase (ReproSil Chiral-MIF, 250 × 10 mm) and eluted with the same solvent system.

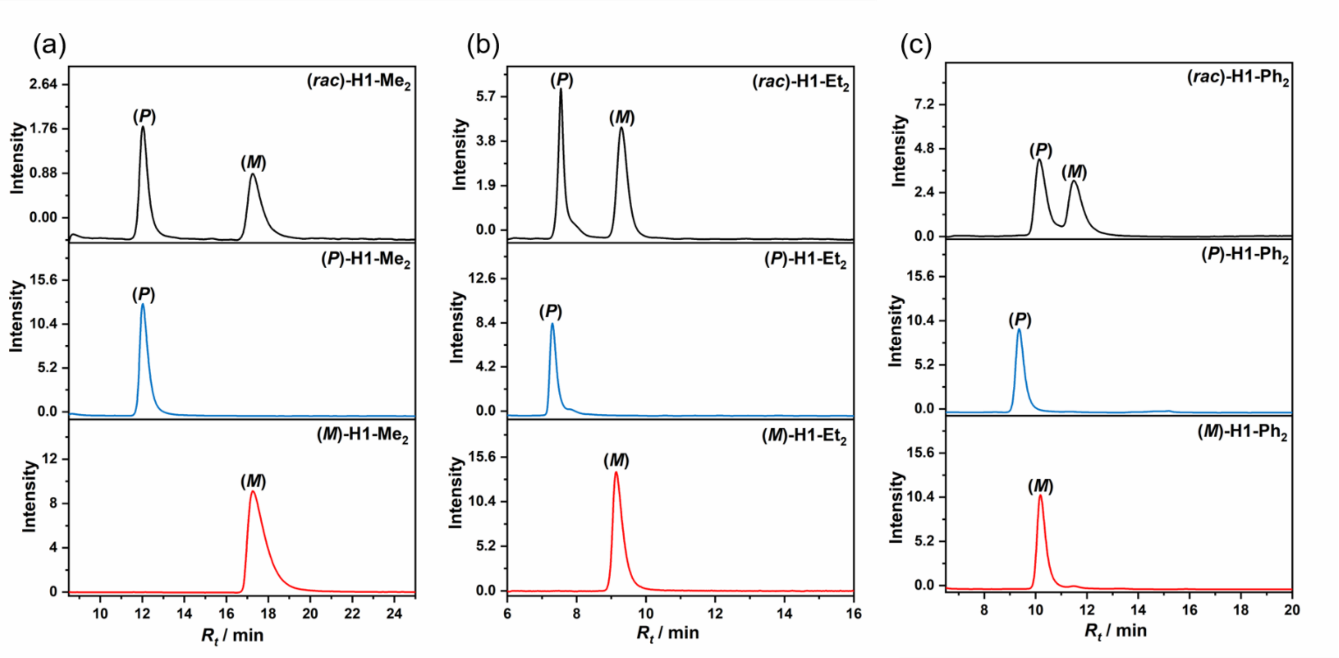

Figure S72. Chromatograms of a) **H1-Me$_2$**, b) **H1-Et$_2$**, and c) **H1-Ph$_2$**: racemates (top) and (*P*)- (middle), and (*M*)-enantiomers (bottom).

Table S2. Parameters for chiral resolution of enantiomers.

Compound	Column[a]	Eluent: *n*-hexane/CH_2Cl_2	First fraction	Second fraction	α[b]	R_s[c]
**(*rac*)-H1-Me$_2$**	ReproSil Chiral-MIF	93:7	*P*	*M*	2.28	6.25
**(*rac*)-H1-Et$_2$**	ReproSil Chiral-MIF	93:7	*P*	*M*	1.56	3.62
**(*rac*)-H1-Ph$_2$**	ReproSil Chiral-MIF	85:15	*P*	*M*	1.23	1.37

[a] Dr-Maisch GmbH, ReproSil Chiral-MIF (250 × 4.6 mm). Sample injection: 20 µL of each racemate **(*rac*)-H1-Me$_2$**, **(*rac*)-H1-Et$_2$** and **(*rac*)-H1-Ph$_2$** in *n*-hexane/CH_2Cl_2 (7:3, ~1 mg / 2.5 mL). Separation conditions: eluent, *n*-hexane/CH_2Cl_2, flow rate 1 mL/min, 20 ºC. [b] Selectivity parameter: $\alpha = (t_{R2} - t_{R0}) / (t_{R1} - t_{R0})$, where t_{R0}, t_{R1} and t_{R2} are elution times for void volume, first fraction and second fraction, respectively. [c] Resolution parameter: $R_s = 2(t_{R2} - t_{R1}) / (w_2 + w_1)$, where w_1 and w_2 denote peak widths for the first and second fractions, respectively.

7. Interconversion Barrier

The perfect baseline separation of the *P*- and *M*-enantiomers of **H1-Me$_2$** (see Section 4) allowed for the determination of the activation parameters for racemization by time-dependent HPLC measurements. To this end, an enantiopure sample of **(*P*)-H1-Me$_2$** (0.07 mg) was dissolved in 1,2-dichlorobenzene (0.60 mL) and heated at 180 °C. Aliquots (20 µL) were periodically removed from the solution and analyzed by HPLC on a chiral stationary phase (ReproSil Chiral-MIF) at room temperature to determine the enantiomeric ratio.

The sample proved chemically stable, as no decomposition was observed over a period of 5 h.
Our studies showed that the compound is configurationally stable at room temperature over a long period of time. In addition, we did not notice any isomerization even upon heating the sample at 160 °C for 20 min. Thus, the racemization process during the the resolution by HPLC can be neglected.

P-M isomerization is a reversible unimolecular reaction and follows firsrt-order kinetics:

$v = \frac{\mathrm{d}(A)}{A_0} = k\mathrm{d}t$,   [Eq. (1)]

where, *A* denotes the concentration of the enantiomer which is present in excess and A_0 is the initial concentration thereof. *k* denotes the reaction rate constant. Integration of the first-order rate law yields:

$\ln\frac{A}{A_0} = -kt$ [Eq. (2)]

Thus, the rate constant *k* at a given temperature can be determined from the exponential decay of the A/A_0 value over time.

$\frac{A}{A_0} = e^{-kt}$ [Eq. (3)]

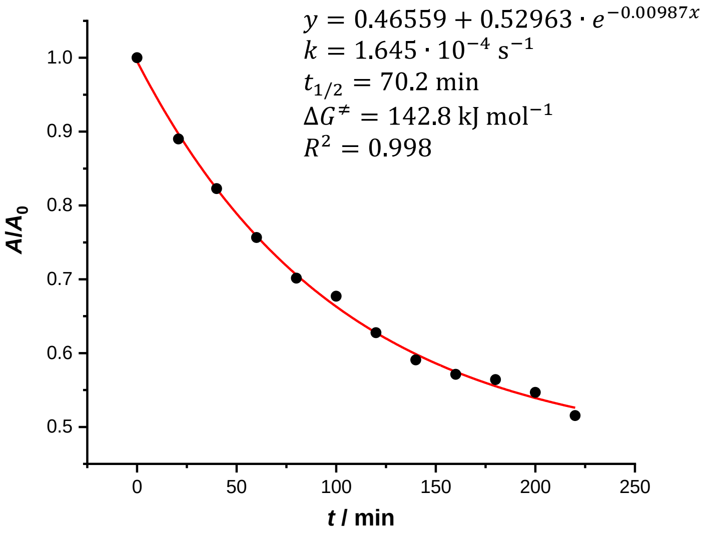

Figure S73. Exponential decay of the A/A_0 value for **(*P*)-H1-Me$_2$** at 180 °C in 1,2-dichlorobenzene over time.

Half-life $t_{1/2}$ can be calculated from the following equation:

$t_{1/2} = \frac{\ln 2}{k}$ [Eq. (4)]

The free activation enthalpy of racemization is determined from the Eyring equation:

$\Delta G^{\neq} = -RT\ln\frac{hk}{\sigma k_\mathrm{B}T}$ [Eq. (5)]

Where *R*, *T*, *h*, *k*, k_B are absolute temperature, gas constant, Planck's constant, rate constant, and Boltzmann constant, respectively. Parameter *σ* was assumed as 0.5, as the probability of conversion to *P*- or *M*-enantiomer is equal.

8. Computations

DFT calculations were performed using the Gaussian 09 program package[11] with B3LYP[12] as a functional and def2-TZVP[13] as a basis set with Grimme's D3BJ dispersion correction.[14] CH_2Cl_2 was used as a solvent for calculations (PCM model). The structures were geometry optimized followed by frequency calculations on the optimized structure. The optimized structures were proven to be minima. **(*P*)-H1-Me$_2$**, **(*P*)-H1-Ph$_2$**, and **(*M*,*M*)-H2-Me$_4$** had no imaginary frequency. Two small imaginary frequencies of −2i. And −3i cm^{-1}. were obtained for **(*M*,*M*)-H2-Ph$_4$** and are considered most likely to be artifacts. Contour plots of selected Kohn–Sham orbitals are presented in Figure S74.
Time-dependent (TD)-DFT calculations were carried out on the optimized structures employing a hybrid exchange-correlation functional using the Coulomb-attenuating method (CAM-B3LYP)[15] with Grimme's D3BJ dispersion correction and def2-TZVP as a basis set. CH_2Cl_2 was used as a solvent for calculations (PCM model). The most stable conformations of **H2** compounds were considered for calculations. The excited state energies as well as the corresponding oscillator strengths (*f*) are listed in Tables S3-S6. The UV/Vis spectra (Figure S75) were simulated using the GaussView 5[16] visualization software package. A half-width of 2000 cm^{-1} was assumed for a proper simulation. The ECD spectra (Figure S76) were simulated using the GaussSum-3.0.2.[17] A half-width of 1600 cm^{-1} was assumed for a proper simulation.

To optimize the stable isomers of **H1-Me₂** and **H2-Me₄** and the transition states of the *P* to *M* interconversion process, DFT calculations were carried out employing the B3LYP functional and the def2-SVP[13] basis set together with Grimme's D3BJ dispersion correction. CH_2Cl_2 was used as a solvent for calculations (PCM model). The character of the obtained structures has been verified by vibrational frequency analysis. **(*P*)-H1-Me$_2$**, **(*M*,*M*)-H2-Me$_4$**, **LM1**, **LM2**, had no imaginary frequency. For transition states **TS** (**H1-Me$_2$**), **TS1** (**H2-Me$_4$**), **TS2** (**H2-Me$_4$**), and **TS3** (**H2-Me$_4$**) one imaginary frequency was obtained in each case.

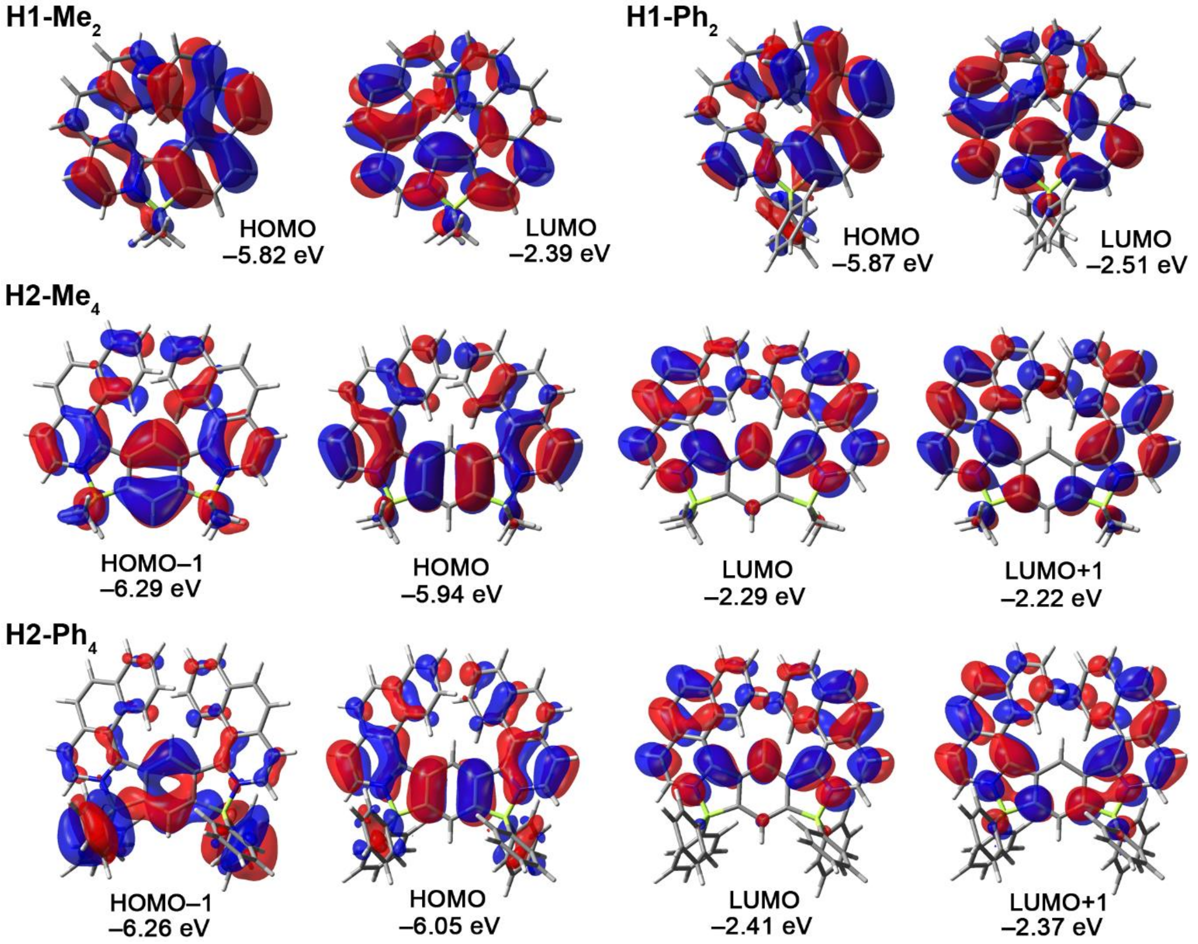

Figure S74. Contour plots of selected Kohn–Sham orbitals (isovalue=0.02 a.u.) of **H1-Me$_2$**, **H1-Ph$_2$**, **H2-Me$_4$**, and **H2-Ph$_4$** calculated at the B3LYP-D3BJ/def2-TZVP (solvent CH_2Cl_2, PCM model) level of theory.

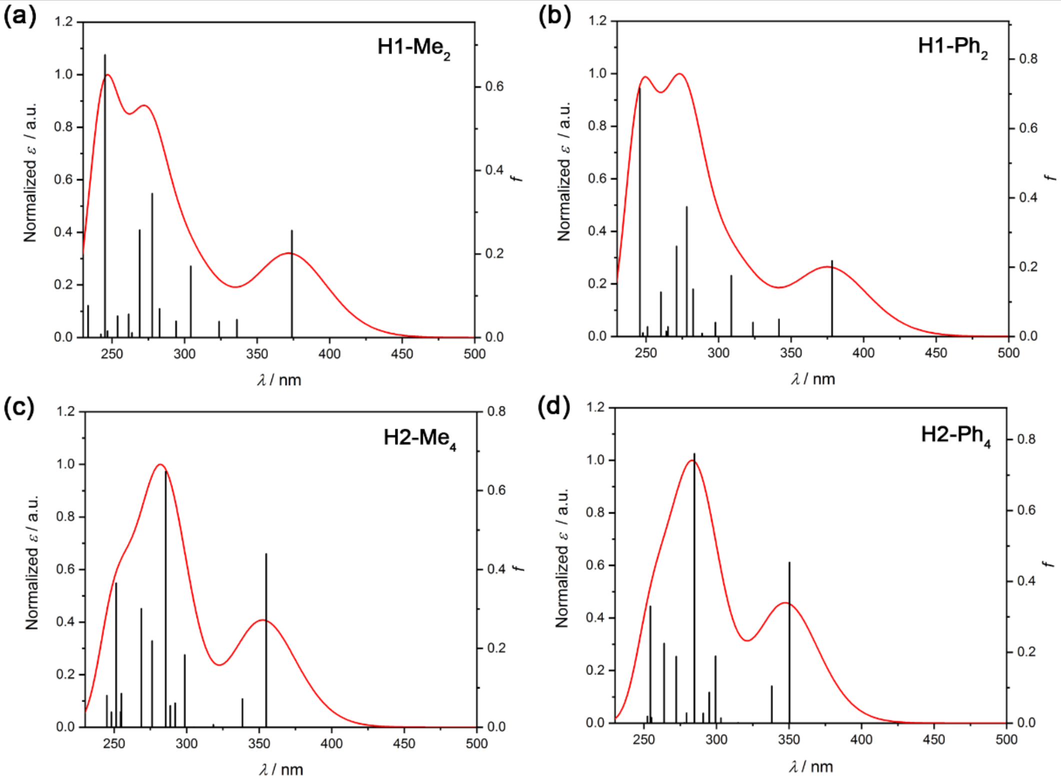

Figure S75. TD-DFT-calculated UV/Vis absorption spectra of **H1-Me$_2$**, **H1-Ph$_2$**, **H2-Me$_4$**, and **H2-Ph$_4$** at the CAM-B3LYP-D3BJ/def2-TZVP (solvent CH_2Cl_2) level of theory.

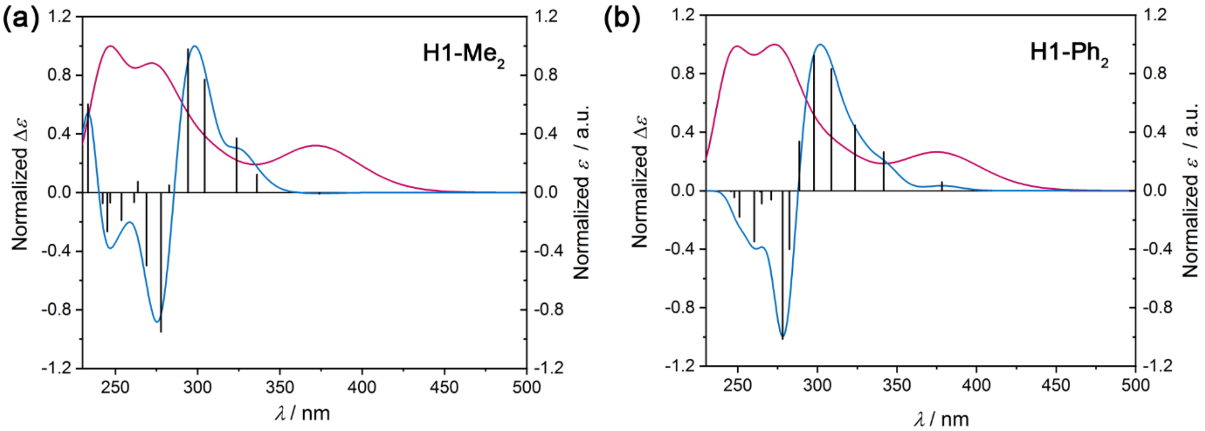

Figure S76. Simulated CD spectra (blue lines) and UV/Vis absorption spectra (magenta lines) of (a) **H1-Me$_2$** and (b) **H1-Ph$_2$** (CAM-B3LYP-D3BJ/def2-TZVP, CH_2Cl_2, PCM model).

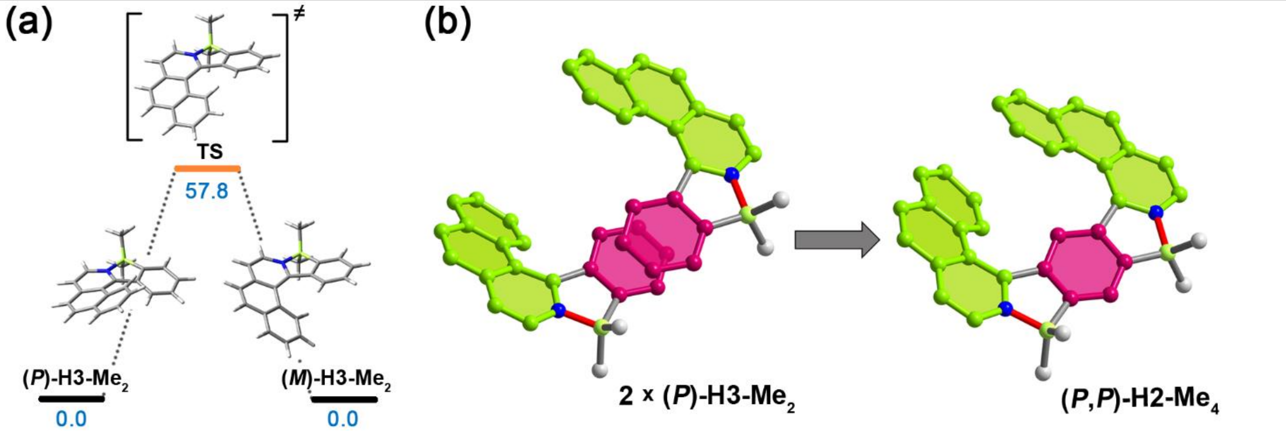

Figure S77. a) *P-M* interconversion pathway of **H3-Me$_2$**. The relative Gibbs free energy is given in kJ mol^{-1}. b) Compound **H2-Me$_4$** as a superposition of two azabora[5]helicenes **H3-Me$_2$** with a joint phenyl ring.

Cartesian coordinates (B3LYP-D3BJ/def2-TZVP, CH_2Cl_2, PCM model)

(*P*)-H1-Me$_2$

Total energy of the optimized structure *E* = -1198.59859520 Hartree

Symbol	X	Y	Z
C	1.230530	−0.824071	0.025604
C	1.297817	0.646738	−0.052916
N	2.582126	1.015454	−0.269754
B	3.622109	−0.181132	0.013964
C	2.524539	−1.313748	0.258699
C	2.700404	−2.657268	0.614121
C	1.606181	−3.476575	0.755724
C	0.318008	−3.037700	0.377807
C	0.128927	−1.719589	−0.120209
C	0.317019	1.666857	0.119369
C	0.650922	2.958072	−0.372211
C	1.979897	3.240633	−0.743834
C	2.928662	2.271106	−0.595580
C	−0.798894	−3.923020	0.487000
C	−2.033174	−3.555122	0.066842
C	−2.222706	−2.317050	−0.617360
C	−1.127062	−1.423868	−0.775890
C	−0.965181	1.492987	0.769691
C	−1.948387	2.509906	0.626446
C	−1.619321	3.724763	−0.046825
C	−0.357331	3.964133	−0.474345

C	−1.266766	0.405913	1.610516
C	−2.508074	0.272622	2.190263
C	−3.507493	1.228180	1.962159
C	−3.220336	2.339610	1.205039
C	−3.468465	−1.998307	−1.194620
C	−3.625144	−0.870380	−1.964521
C	−2.518442	−0.044484	−2.214170
C	−1.301426	−0.322332	−1.637196
C	4.497831	0.228964	1.320490
C	4.529663	−0.473928	−1.300232
H	3.694916	−3.037003	0.816647
H	1.716533	−4.492267	1.117066
H	2.250532	4.227821	−1.089478
H	3.982085	2.447598	−0.754798
H	−0.633900	−4.898432	0.927915
H	−2.880575	−4.219174	0.182755
H	−2.388703	4.479267	−0.153433
H	−0.087688	4.917931	−0.908478
H	−0.511383	−0.333999	1.816696
H	−2.709585	−0.578258	2.827412
H	−4.487334	1.105738	2.404324
H	−3.963226	3.114095	1.061349
H	−4.299501	−2.675283	−1.038568
H	−4.586105	−0.638104	−2.404739
H	−2.619174	0.812582	−2.867073
H	−0.459652	0.311638	−1.864806
H	5.152931	1.085227	1.127562
H	3.877042	0.475689	2.187527
H	5.142405	−0.607000	1.609487
H	5.169023	−1.345920	−1.132548
H	3.929274	−0.678900	−2.192093
H	5.193468	0.365976	−1.533436

(*P*)-H1-Ph$_2$
Total energy of the optimized structure *E* = -1582.25521135 Hartree

Symbol	X	Y	Z
C	−0.033466	−0.760451	0.273525
C	0.006762	0.582284	−0.336275

N	1.283015	0.867082	−0.686892
B	2.340753	−0.102853	0.031457
C	1.267389	−1.110998	0.657297
C	1.475126	−2.224480	1.477464
C	0.398116	−2.956571	1.916319
C	−0.900229	−2.706682	1.420017
C	−1.118614	−1.663828	0.478988
C	−0.988523	1.579662	−0.538680
C	−0.680236	2.602260	−1.477859
C	0.640291	2.744989	−1.946377
C	1.607870	1.914228	−1.461464
C	−1.999850	−3.507594	1.859361
C	−3.243817	−3.334762	1.351567
C	−3.461907	−2.433870	0.266161
C	−2.384233	−1.644468	−0.222015
C	−2.262292	1.639489	0.147505
C	−3.263375	2.519119	−0.347054
C	−2.961157	3.401999	−1.427268
C	−1.707487	3.483547	−1.931902
C	−2.536431	0.934286	1.333390
C	−3.769797	1.009368	1.940015
C	−4.786578	1.802397	1.391386
C	−4.526148	2.559641	0.273239
C	−4.717473	−2.364714	−0.370728
C	−4.899682	−1.597245	−1.496522
C	−3.810035	−0.903667	−2.044782
C	−2.583955	−0.935270	−1.422959
C	3.105321	0.734687	1.185826
C	3.301798	−0.771843	−1.083897
C	2.917674	−1.923087	−1.783453
C	3.704754	−2.466397	−2.793915
C	4.910410	−1.863867	−3.138405
C	5.315298	−0.717803	−2.462894
C	4.519930	−0.187019	−1.451731
C	2.639798	1.950447	1.699300
C	3.272306	2.591321	2.761391
C	4.396642	2.024450	3.349662
C	4.877764	0.812841	2.862525

C	4.240102	0.185427	1.798491
H	2.477813	−2.480590	1.796679
H	0.530977	−3.769705	2.620001
H	0.890388	3.535962	−2.638256
H	2.657464	2.028963	−1.686704
H	−1.813716	−4.252065	2.623502
H	−4.077944	−3.922523	1.713806
H	−3.744861	4.052669	−1.794751
H	−1.458859	4.211530	−2.692744
H	−1.766189	0.332182	1.785994
H	−3.951160	0.450941	2.848705
H	−5.759318	1.841093	1.863365
H	−5.283131	3.217974	−0.133830
H	−5.535079	−2.949856	0.032081
H	−5.867608	−1.555524	−1.978692
H	−3.930722	−0.346012	−2.964208
H	−1.754824	−0.417611	−1.877858
H	3.378891	−3.360599	−3.311749
H	6.252360	−0.239214	−2.721365
H	2.884430	3.533551	3.129798
H	5.752996	0.358035	3.311029
H	5.527981	−2.284255	−3.922327
H	1.981896	−2.408249	−1.532043
H	4.861198	0.698805	−0.929619
H	4.637521	−0.753484	1.431520
H	1.759967	2.416625	1.271584
H	4.892283	2.519087	4.175535

(*M*,*M*)-H2-Me$_4$
Total energy of the optimized structure E = -1549.99642702 Hartree

Symbol	X	Y	Z
C	1.190126	1.322517	0.123702
C	1.211934	2.737057	0.062677
C	0.000031	3.418522	0.000099
C	−1.211896	2.737080	0.062820
C	−1.190108	1.322546	0.123713
C	0.000000	0.614465	0.000038
C	2.559244	0.792423	0.150282

C	−2.559229	0.792449	0.150233
C	3.053074	0.542385	0.222570
C	4.373861	0.756401	0.253742
C	5.212961	0.350056	0.495079
C	4.725029	1.610675	0.312785
N	3.423793	1.812374	0.041343
C	2.314982	−1.686772	0.721534
C	2.783115	−2.992230	0.407125
C	4.042158	−3.152371	0.247410
C	4.837003	−2.085035	0.498018
N	−3.423770	1.812426	0.041357
C	−4.725006	1.610741	0.312768
C	−5.212965	0.350126	0.495040
C	−4.373885	0.756344	0.253706
C	−3.053069	0.542341	0.222548
C	0.924159	−3.976110	1.605768
C	0.534491	−2.697434	2.027396
B	2.715806	3.270667	0.007486
B	−2.715760	3.270711	0.007543
C	3.059438	4.089919	−1.350664
C	−3.215211	4.027895	−1.356537
C	1.220281	−1.582480	1.600949
C	2.047997	−4.117929	0.827345
C	−4.837076	−2.084968	0.497944
C	−4.042246	−3.152317	0.247353
C	−2.783175	−2.992192	0.407137
C	−2.314992	−1.686748	0.721519
C	−2.048082	−4.117915	0.827342
C	0.924219	−3.976133	−1.605735
C	0.534502	−2.697466	−2.027343
C	−1.220266	−1.582491	−1.600904
H	0.364083	−4.845850	−1.921673
H	0.306991	−2.582575	−2.697564
H	0.922826	0.611911	−1.964286
H	−2.402593	−5.102022	0.547699
H	−4.379764	−4.155380	0.476570
H	−5.828760	−2.214502	0.911048
H	−6.235436	0.198012	0.809516

H	0.306983	−2.582527	2.697638
H	0.922906	0.611886	1.964352
H	0.364005	−4.845811	1.921720
H	2.402463	−5.102049	0.547693
H	4.379648	−4.155436	0.476661
H	5.828660	−2.214594	0.911180
H	6.235414	0.197919	0.809603
H	5.329281	2.497467	0.432248
H	0.000038	0.457749	0.000117
H	0.000048	4.504557	0.000140
H	−5.329256	2.497537	0.432241
H	−3.025307	3.446937	−2.264644
H	−4.286583	4.255887	−1.327137
H	−2.687874	4.980806	−1.464385
H	4.118728	4.361003	−1.420384
H	2.799777	3.535613	−2.258382
H	2.491855	5.025337	−1.371442
C	−3.059263	4.089868	1.350747
H	−2.799787	3.535405	2.258413
H	−2.491541	5.025197	1.371709
H	−4.118524	4.361103	1.420404
C	3.215195	4.027728	1.356632
H	4.286554	4.255800	1.327105
H	2.687821	4.980582	1.464752
H	3.025479	3.446584	2.264651

(*M*,*M*)-H2-Ph$_4$

Total energy of the optimized structure *E* = -2317.31153905 Hartree

Symbol	X	Y	Z
C	1.181570	−0.311422	0.091104
C	1.249765	1.099299	0.041477
C	0.068857	1.829349	0.010135
C	−1.163776	1.190721	−0.025824
C	−1.202732	−0.221087	−0.081579
C	−0.037362	−0.974442	0.003423
C	2.532954	−0.887211	0.060710
C	−2.594480	−0.692731	−0.060177
C	2.983579	−2.236290	0.084926
C	4.277106	−2.482395	−0.449849

C	5.142154	−1.399269	−0.702813
C	4.705833	−0.127285	−0.476070
N	3.424554	0.107730	−0.143661
C	2.225018	−3.366000	0.586013
C	2.634510	−4.678018	0.222284
C	3.860511	−4.864204	−0.486140
C	4.682630	−3.819381	−0.744191
N	−3.408878	0.367342	0.139389
C	−4.707444	0.231245	0.459653
C	−5.242577	−1.003515	0.679729
C	−4.461021	−2.149554	0.432901
C	−3.147531	−2.003326	−0.090188
C	0.789536	−5.626041	1.469120
C	0.460196	−4.345867	1.934096
B	2.774455	1.578137	−0.005283
B	−2.647456	1.784049	0.011435
C	3.175322	2.458324	−1.300120
C	3.219559	2.223322	1.409856
C	−2.992281	2.687882	1.305809
C	−3.026611	2.464083	−1.406411
C	1.169693	−3.244987	1.509622
C	1.878065	−5.787862	0.645570
C	−4.971360	−3.451317	0.721755
C	−4.229541	−4.556306	0.471659
C	−2.986578	−4.465098	−0.225654
C	−2.474839	−3.188538	−0.586225
C	−2.314345	−5.629935	−0.643209
C	−1.210864	−5.552707	−1.459097
C	−0.781946	−4.301968	−1.922989
C	−1.407906	−3.149663	−1.503516
C	2.441387	2.377275	−2.490199
C	2.807858	3.085756	−3.629957
C	3.933495	3.903170	−3.610421
C	4.680958	4.003474	−2.442077
C	4.300521	3.291465	−1.308263
C	3.888111	1.520319	2.416882
C	4.189149	2.104778	3.645598
C	3.819071	3.418606	3.903783

C	3.147335	4.139510	2.919801
C	2.857626	3.547077	1.697116
C	−4.043652	3.612507	1.303051
C	−4.381131	4.346400	2.436547
C	−3.663620	4.175684	3.615474
C	−2.610575	3.266848	3.645876
C	−2.286966	2.537242	2.506449
C	−3.723892	1.811335	−2.427521
C	−3.962422	2.418288	−3.659061
C	−3.498852	3.704481	−3.905306
C	−2.796197	4.374685	−2.907193
C	−2.569107	3.760357	−1.682057
H	−0.696818	−6.451913	−1.770334
H	0.041709	−4.238058	−2.621458
H	−1.085764	−2.200989	−1.902164
H	−2.700667	−6.591639	−0.329886
H	−4.600891	−5.538753	0.734995
H	−5.953652	−3.529673	1.168524
H	−6.258861	−1.104255	1.032167
H	−0.351867	−4.218934	2.637490
H	0.923212	−2.274392	1.909532
H	0.210570	−6.483112	1.785229
H	2.186918	−6.776530	0.330409
H	4.152600	−5.872176	−0.753073
H	5.651366	−3.972706	−1.201045
H	6.144472	−1.577504	−1.064440
H	5.329152	0.744085	−0.607590
H	−0.077530	−2.045532	−0.000907
H	0.110117	2.913002	0.011657
H	−5.263002	1.147806	0.587839
H	−1.458617	1.839989	2.549772
H	−2.039470	3.129682	4.556330
H	−3.918243	4.748001	4.498867
H	−5.200802	5.054051	2.398923
H	−4.605580	3.770742	0.390129
H	2.339359	4.128529	0.943501
H	2.850360	5.164554	3.106937
H	4.050300	3.876535	4.857328

H	4.712688	1.530787	4.400984
H	4.184993	0.491246	2.251869
H	4.887831	3.395413	−0.403537
H	5.557436	4.639867	−2.412628
H	4.221571	4.459234	−4.493913
H	2.213196	3.004131	−4.531972
H	1.556750	1.752519	−2.524853
H	−4.091357	0.803867	−2.271671
H	−4.510343	1.883549	−4.425859
H	−3.681470	4.179998	−4.860806
H	−2.426576	5.377494	−3.085268
H	−2.024382	4.302272	−0.917791

Table S3. TD-DFT-calculated UV/Vis absorption data for ***(P)*-H1-Me$_2$** at the CAM-B3LYP-D3BJ/def2-TZVP (solvent CH_2Cl_2) level.

Calcd. *λ* [nm]	*f*	composition	coefficient	contribution
373.87	0.2564	HOMO–2 –> LUMO	0.14063	4%
		HOMO–1 –> LUMO	0.12976	3%
		HOMO –> LUMO	0.64961	84%
335.91	0.0430	HOMO–2 –> LUMO	0.28061	16%
		HOMO–1 –> LUMO	0.50706	51%
		HOMO–1 –> LUMO+2	0.14988	4%
		HOMO –> LUMO	–0.10647	2%
		HOMO –> LUMO+1	–0.28393	16%
		HOMO –> LUMO+3	–0.10238	2%
323.70	0.0382	HOMO–3 –> LUMO+1	–0.16898	6%
		HOMO–2 –> LUMO	–0.27516	15%
		HOMO–1 –> LUMO	0.36973	27%
		HOMO–1 –> LUMO+1	0.23769	11%
		HOMO –> LUMO+1	0.39133	31%
304.16	0.1706	HOMO–3 –> LUMO	0.30246	18%
		HOMO–2 –> LUMO	0.46039	42%
		HOMO–2 –> LUMO+1	–0.23064	11%
		HOMO –> LUMO	–0.12845	3%
		HOMO –> LUMO+1	0.27167	15%
		HOMO –> LUMO+3	0.12898	3%
294.06	0.0391	HOMO–3 –> LUMO	0.32364	21%
		HOMO–2 –> LUMO	–0.17541	6%
		HOMO–2 –> LUMO+1	–0.20009	8%

		HOMO–1 –> LUMO+2	–0.14197	4%
		HOMO–1 –> LUMO+3	0.11075	2%
		HOMO –> LUMO+1	–0.24426	12%
		HOMO –> LUMO+2	0.43978	39%
282.60	0.0690	HOMO–4 –> LUMO	0.1285	3%
		HOMO–3 –> LUMO	–0.16714	6%
		HOMO–3 –> LUMO+1	0.23187	11%
		HOMO–2 –> LUMO	0.1152	3%
		HOMO–2 –> LUMO+1	0.34922	24%
		HOMO–1 –> LUMO+2	–0.26201	14%
		HOMO –> LUMO	–0.12231	3%
		HOMO –> LUMO+1	0.18950	7%
		HOMO –> LUMO+2	0.27881	16%
		HOMO –> LUMO+4	0.14446	4%
277.61	0.3449	HOMO–3 –> LUMO+1	–0.17590	6%
		HOMO–3 –> LUMO+2	0.12079	3%
		HOMO–2 –> LUMO	0.15616	5%
		HOMO–2 –> LUMO+1	0.15006	5%
		HOMO–1 –> LUMO	–0.24944	12%
		HOMO–1 –> LUMO+1	0.33476	22%
		HOMO–1 –> LUMO+2	0.21462	9%
		HOMO –> LUMO+2	0.26025	14%
		HOMO –> LUMO+3	–0.22891	10%
268.89	0.2577	HOMO–3 –> LUMO	0.29257	17%
		HOMO–2 –> LUMO+1	0.29267	17%
		HOMO–1 –> LUMO+1	0.35699	25%
		HOMO–1 –> LUMO+2	–0.17379	6%
		HOMO –> LUMO+1	–0.1869	7%
		HOMO –> LUMO+2	–0.30170	18%
263.66	0.0114	HOMO–6 –> LUMO	–0.10267	2%
		HOMO–4 –> LUMO	0.15045	5%
		HOMO–3 –> LUMO	–0.35155	25%
		HOMO–2 –> LUMO+1	–0.32518	21%
		HOMO–1 –> LUMO+1	0.38549	30%
		HOMO–1 –> LUMO+2	–0.12659	3%
		HOMO –> LUMO+3	0.16116	5%
261.29	0.0560	HOMO–4 –> LUMO	0.37123	28%
		HOMO–3 –> LUMO	0.12164	3%

		HOMO–3 –> LUMO+1	0.29752	18%
		HOMO–2 –> LUMO+3	–0.16293	5%
		HOMO–1 –> LUMO+2	0.28102	16%
		HOMO –> LUMO+1	0.14370	4%
		HOMO –> LUMO+3	–0.19907	8%
253.71	0.0518	HOMO–6 –> LUMO	–0.34812	24%
		HOMO–4 –> LUMO	0.35406	25%
		HOMO–4 –> LUMO+1	0.23752	11%
		HOMO–3 –> LUMO	0.11130	2%
		HOMO–3 –> LUMO+1	–0.25916	13%
		HOMO–2 –> LUMO+1	0.11931	3%
		HOMO–2 –> LUMO+2	0.10712	2%
246.79	0.0155	HOMO–6 –> LUMO	0.53657	58%
		HOMO–6 –> LUMO+2	0.10597	2%
		HOMO–4 –> LUMO	0.24483	12%
		HOMO–4 –> LUMO+1	0.15816	5%
		HOMO–3 –> LUMO+1	–0.13625	4%
245.00	0.6766	HOMO–3 –> LUMO+1	0.11599	3%
		HOMO–2 –> LUMO+1	0.15121	5%
		HOMO–1 –> LUMO+2	0.37631	28%
		HOMO –> LUMO+3	0.50935	52%
242.26	0.0082	HOMO–5 –> LUMO	0.52520	55%
		HOMO–5 –> LUMO+1	0.10573	2%
		HOMO–3 –> LUMO+1	–0.20634	9%
		HOMO–2 –> LUMO+2	–0.12510	3%
		HOMO –> LUMO+4	0.11797	3%
		HOMO –> LUMO+5	0.10849	2%
233.37	0.0762	HOMO–5 –> LUMO	–0.18452	7%
		HOMO–4 –> LUMO	0.17112	6%
		HOMO–4 –> LUMO+3	–0.10095	2%
		HOMO–3 –> LUMO+1	–0.18667	7%
		HOMO–3 –> LUMO+2	0.18081	7%
		HOMO–2 –> LUMO+2	–0.31909	20%
		HOMO–1 –> LUMO+3	0.36239	26%
		HOMO–1 –> LUMO+4	0.14651	4%

Table S4. TD-DFT-calculated UV/Vis absorption data for ***(P)*****-H1-Ph$_2$** at the CAM-B3LYP-D3BJ/def2-TZVP (solvent CH_2Cl_2) level.

Calcd. *λ* [nm]	*f*	composition	coefficient	contribution
378.24	0.2185	HOMO–2 –> LUMO	0.12191	3%
		HOMO–1 –> LUMO	0.14986	4%
		HOMO –> LUMO	0.64763	84%
341.55	0.0500	HOMO–2 –> LUMO	0.22850	10%
		HOMO–1 –> LUMO	0.55134	61%
		HOMO–1 –> LUMO+2	–0.13593	4%
		HOMO –> LUMO	–0.13008	3%
		HOMO –> LUMO+1	0.24304	12%
323.64	0.0406	HOMO–4 –> LUMO+1	0.14919	4%
		HOMO–3 –> LUMO	0.10953	2%
		HOMO–2 –> LUMO	0.30853	19%
		HOMO–1 –> LUMO	–0.30650	19%
		HOMO–1 –> LUMO+1	0.24936	12%
		HOMO –> LUMO+1	0.39451	31%
308.69	0.1758	HOMO–6 –> LUMO	–0.13273	4%
		HOMO–4 –> LUMO	–0.25223	13%
		HOMO–2 –> LUMO	0.47142	44%
		HOMO–2 –> LUMO+1	0.20461	8%
		HOMO –> LUMO	–0.11919	3%
		HOMO –> LUMO+1	–0.28056	16%
		HOMO –> LUMO+3	–0.12312	3%
297.74	0.0403	HOMO–4 –> LUMO	0.24394	12%
		HOMO–3 –> LUMO	0.37396	28%
		HOMO–2 –> LUMO+1	–0.15832	5%
		HOMO –> LUMO+1	–0.27041	15%
		HOMO –> LUMO+2	0.35377	25%
288.51	0.0089	HOMO–6 –> LUMO	−0.10388	2%
		HOMO–4 –> LUMO	–0.15946	5%
		HOMO–4 –> LUMO+1	0.17058	6%
		HOMO–3 –> LUMO	0.41520	34%
		HOMO–3 –> LUMO+2	–0.10449	2%
		HOMO–2 –> LUMO	–0.14044	4%
		HOMO–1 –> LUMO+2	0.21626	9%
		HOMO –> LUMO+2	–0.26572	14%
		HOMO –> LUMO+3	–0.11875	3%
		HOMO –> LUMO+4	–0.11859	3%

		HOMO–6 –> LUMO	–0.12543	3%
		HOMO–4 –> LUMO	–0.19692	8%
		HOMO–3 –> LUMO	0.13403	4%
		HOMO–2 –> LUMO	–0.17372	6%
282.39	0.1370	HOMO–2 –> LUMO+1	0.37453	28%
		HOMO–1 –> LUMO+1	0.14212	4%
		HOMO–1 –> LUMO+2	–0.15911	5%
		HOMO –> LUMO+1	0.15211	5%
		HOMO –> LUMO+2	0.30858	19%
		HOMO–5 –> LUMO	0.10584	2%
		HOMO–4 –> LUMO+1	0.18610	7%
		HOMO–3 –> LUMO	–0.31638	20%
		HOMO–1 –> LUMO	0.19603	8%
278.05	0.3745	HOMO–1 –> LUMO+1	0.31720	20%
		HOMO–1 –> LUMO+2	0.22310	10%
		HOMO –> LUMO+2	0.21111	9%
		HOMO –> LUMO+3	–0.19415	8%
		HOMO–6 –> LUMO	0.11436	3%
		HOMO–4 –> LUMO	0.29242	17%
		HOMO–2 –> LUMO+1	0.27763	15%
270.88	0.2606	HOMO–1 –> LUMO+1	0.36986	27%
		HOMO–1 –> LUMO+2	–0.13361	4%
		HOMO –> LUMO+1	–0.19707	8%
		HOMO –> LUMO+2	–0.29658	18%
		HOMO–8 –> LUMO	0.22835	10%
		HOMO–5 –> LUMO	0.49719	49%
265.02	0.0284	HOMO–4 –> LUMO	–0.22059	10%
		HOMO–1 –> LUMO+2	–0.24333	12%
		HOMO –> LUMO+3	0.15677	5%
		HOMO–6 –> LUMO	0.15290	5%
		HOMO–5 –> LUMO	0.19447	8%
		HOMO–4 –> LUMO	0.26818	14%
		HOMO–3 –> LUMO+1	0.10914	2%
263.95	0.0151	HOMO–2 –> LUMO+1	0.35515	25%
		HOMO–1 –> LUMO+1	–0.34371	24%
		HOMO–1 –> LUMO+2	0.15045	5%
		HOMO –> LUMO+1	0.11951	3%

		HOMO –> LUMO+3	–0.16144	5%
		HOMO–8 –> LUMO	0.20243	8%
		HOMO–8 –> LUMO+1	0.16165	5%
		HOMO–6 –> LUMO+1	0.17349	6%
		HOMO–5 –> LUMO	–0.26822	14%
		HOMO–4 –> LUMO+1	0.30007	18%
		HOMO–3 –> LUMO+1	0.19998	8%
260.25	0.1279	HOMO–2 –> LUMO	–0.10557	2%
		HOMO–2 –> LUMO+1	0.10675	2%
		HOMO–2 –> LUMO+2	0.11727	3%
		HOMO–2 –> LUMO+3	0.16072	5%
		HOMO–1 –> LUMO+2	–0.15064	5%
		HOMO –> LUMO+1	–0.13971	4%
		HOMO –> LUMO+3	0.12450	3%
		HOMO–8 –> LUMO	0.42411	36%
		HOMO–8 –> LUMO+1	–0.16446	5%
		HOMO–7 –> LUMO	0.10880	2%
250.95	0.0284	HOMO–6 –> LUMO	0.19848	8%
		HOMO–5 –> LUMO	–0.24312	12%
		HOMO–5 –> LUMO+1	–0.13983	4%
		HOMO–3 –> LUMO+1	–0.22955	11%
		HOMO–8 –> LUMO	–0.20542	8%
247.70	0.0107	HOMO–7 –> LUMO	0.15113	5%
		HOMO–6 –> LUMO	0.55462	62%
		HOMO–4 –> LUMO	–0.25780	13%
		HOMO–4 –> LUMO+1	–0.10698	2%
245.64	0.7156	HOMO–2 –> LUMO+1	0.13776	4%
		HOMO–1 –> LUMO+2	0.39577	31%
		HOMO –> LUMO+3	0.49400	49%

Table S5. TD-DFT-calculated UV/Vis absorption data for ***(M,M)*-H2-Me$_4$** at the CAM-B3LYP-D3BJ/def2-TZVP (solvent CH_2Cl_2) level.

Calcd. *λ* [nm]	*f*	composition	coefficient	contribution
		HOMO–4 –> LUMO+1	–0.10492	2%
		HOMO–2 –> LUMO	0.11904	3%
354.70	0.4400	HOMO–1 –> LUMO+1	–0.14886	4%
		HOMO –> LUMO	0.63159	80%
		HOMO –> LUMO+2	0.11629	3%
338.34	0.0719	HOMO–4 –> LUMO	–0.10849	2%

		HOMO–3 –> LUMO	0.10688	2%
		HOMO–3 –> LUMO+2	0.10637	2%
		HOMO–2 –> LUMO+3	0.12545	3%
		HOMO–1 –> LUMO	–0.27525	15%
		HOMO –> LUMO+1	0.58185	68%
318.33	0.0069	HOMO–5 –> LUMO	–0.11396	3%
		HOMO–4 –> LUMO+3	–0.14219	4%
		HOMO–3 –> LUMO+1	–0.12322	3%
		HOMO–3 –> LUMO+3	–0.10648	2%
		HOMO–2 –> LUMO	–0.15861	5%
		HOMO–2 –> LUMO+2	–0.11207	3%
		HOMO–1 –> LUMO+1	0.30782	19%
		HOMO –> LUMO+2	0.50992	52%
304.91	0.0005	HOMO–4 –> LUMO+2	–0.13043	3%
		HOMO–3 –> LUMO	–0.34706	24%
		HOMO–2 –> LUMO+1	–0.30179	18%
		HOMO–1 –> LUMO	–0.13605	4%
		HOMO–1 –> LUMO+2	–0.29014	17%
		HOMO –> LUMO+3	0.35135	25%
298.55	0.1835	HOMO–5 –> LUMO	–0.10915	2%
		HOMO–3 –> LUMO+1	0.38625	30%
		HOMO–2 –> LUMO	0.45131	41%
		HOMO–2 –> LUMO+2	–0.16564	5%
		HOMO–1 –> LUMO+1	0.21358	9%
		HOMO–1 –> LUMO+3	0.11091	2%
291.92	0.0614	HOMO–4 –> LUMO+1	–0.22185	10%
		HOMO–2 –> LUMO	–0.10649	2%
		HOMO–2 –> LUMO+2	0.12541	3%
		HOMO–1 –> LUMO+1	0.47145	44%
		HOMO–1 –> LUMO+3	0.25261	13%
		HOMO–1 –> LUMO+4	–0.12038	3%
		HOMO –> LUMO	0.13832	4%
		HOMO –> LUMO+2	–0.24495	12%
288.66	0.0548	HOMO–4 –> LUMO	–0.13836	4%
		HOMO–3 –> LUMO	–0.23290	11%
		HOMO–2 –> LUMO+1	–0.14856	4%
		HOMO–1 –> LUMO	0.49555	49%
		HOMO–1 –> LUMO+2	0.19936	8%

		HOMO –> LUMO+1	0.28583	16%
285.47	0.6487	HOMO–5 –> LUMO+1	–0.19737	8%
		HOMO–4 –> LUMO	0.20932	9%
		HOMO–4 –> LUMO+2	–0.10126	2%
		HOMO–3 –> LUMO+2	–0.29455	17%
		HOMO–2 –> LUMO+1	0.33850	23%
		HOMO–2 –> LUMO+3	–0.20422	8%
		HOMO–1 –> LUMO	0.10816	2%
		HOMO–1 –> LUMO+2	–0.14345	4%
		HOMO –> LUMO+1	0.16356	5%
		HOMO –> LUMO+3	0.21415	9%
		HOMO –> LUMO+4	–0.14179	4%
276.08	0.2189	HOMO–5 –> LUMO	0.13587	4%
		HOMO–4 –> LUMO+1	–0.12194	3%
		HOMO–3 –> LUMO+3	0.28130	16%
		HOMO–2 –> LUMO	0.18474	7%
		HOMO–2 –> LUMO+2	0.43816	38%
		HOMO –> LUMO	–0.16640	6%
		HOMO –> LUMO+2	0.28055	16%
268.71	0.3010	HOMO–4 –> LUMO	–0.15711	5%
		HOMO–3 –> LUMO	0.30337	18%
		HOMO–3 –> LUMO+2	0.25848	13%
		HOMO–2 –> LUMO+3	0.20330	8%
		HOMO–1 –> LUMO	0.32973	22%
		HOMO–1 –> LUMO+2	–0.20024	8%
		HOMO –> LUMO+3	0.30205	18%
254.92	0.0856	HOMO–5 –> LUMO+1	0.10735	2%
		HOMO–5 –> LUMO+3	–0.10693	2%
		HOMO–3 –> LUMO+2	–0.14004	4%
		HOMO–1 –> LUMO+2	0.45246	41%
		HOMO –> LUMO+3	0.43553	38%
254.35	0.0389	HOMO–6 –> LUMO+1	0.13105	3%
		HOMO–5 –> LUMO	–0.11518	3%
		HOMO–5 –> LUMO+2	0.15725	5%
		HOMO–4 –> LUMO+1	0.27618	15%
		HOMO–3 –> LUMO+1	–0.27011	15%
		HOMO–2 –> LUMO	0.26150	14%
		HOMO–2 –> LUMO+2	0.14951	4%

		HOMO–1 –> LUMO+1	0.18718	7%
		HOMO–1 –> LUMO+3	–0.27191	15%
		HOMO –> LUMO+2	–0.18411	7%
251.37	0.3656	HOMO–4 –> LUMO	0.47694	45%
		HOMO–4 –> LUMO+2	0.23734	11%
		HOMO–3 –> LUMO	–0.18793	7%
		HOMO–3 –> LUMO+2	0.15566	5%
		HOMO–2 –> LUMO+3	0.21725	9%
		HOMO–1 –> LUMO	0.10341	2%
248.06	0.0384	HOMO–8 –> LUMO	0.16001	5%
		HOMO–7 –> LUMO	–0.14563	4%
		HOMO–6 –> LUMO+1	0.40108	32%
		HOMO–6 –> LUMO+3	0.13517	4%
		HOMO–6 –> LUMO+4	–0.14659	4%
		HOMO–3 –> LUMO+1	–0.16547	5%
		HOMO–1 –> LUMO+1	–0.10817	2%
		HOMO–1 –> LUMO+3	0.35281	25%
		HOMO –> LUMO+2	0.10982	2%
244.90	0.0806	HOMO–8 –> LUMO+1	0.14189	4%
		HOMO–6 –> LUMO	0.21751	9%
		HOMO–5 –> LUMO+1	0.28434	16%
		HOMO–4 –> LUMO	–0.21654	9%
		HOMO–4 –> LUMO+2	0.14528	4%
		HOMO–3 –> LUMO	–0.14941	4%
		HOMO–3 –> LUMO+2	–0.23580	11%
		HOMO–2 –> LUMO+1	0.16588	6%
		HOMO–1 –> LUMO+2	–0.24686	12%
		HOMO–1 –> LUMO+5	0.10329	2%
		HOMO –> LUMO+4	0.17126	6%

Table S6. TD-DFT-calculated UV/Vis absorption data for ***(M,M)*-H2-Ph$_4$** at the CAM-B3LYP-D3BJ/def2-TZVP (solvent CH_2Cl_2) level.

Calcd. *λ* [nm]	*f*	composition	coefficient	contribution
350.19	0.4536	HOMO−7 –> LUMO+1	0.11837	3%
		HOMO−4 –> LUMO	−0.14432	4%
		HOMO−2 –> LUMO+1	0.12904	3%
		HOMO−1 –> LUMO+1	−0.10345	2%
		HOMO –> LUMO	0.61622	76%
		HOMO –> LUMO+2	−0.10918	2%

337.97	0.1048	HOMO−7 −> LUMO	0.13830	4%
		HOMO−5 −> LUMO+2	−0.11056	2%
		HOMO−4 −> LUMO+3	−0.11067	2%
		HOMO−2 −> LUMO	0.18870	7%
		HOMO−1 −> LUMO	−0.18584	7%
		HOMO −> LUMO+1	0.58547	69%
314.74	0.0015	HOMO−13 −> LUMO	−0.11400	3%
		HOMO−5 −> LUMO+3	0.13356	4%
		HOMO−4 −> LUMO	−0.16550	5%
		HOMO−2 −> LUMO+1	0.23663	11%
		HOMO−1 −> LUMO+1	−0.33492	22%
		HOMO −> LUMO+2	0.43121	37%
302.95	0.0144	HOMO−7 −> LUMO+2	0.12179	3%
		HOMO−5 −> LUMO	0.36127	26%
		HOMO−4 −> LUMO+1	−0.33951	23%
		HOMO−2 −> LUMO	0.18005	6%
		HOMO−2 −> LUMO+2	0.13856	4%
		HOMO−1 −> LUMO+2	−0.15656	5%
		HOMO −> LUMO+1	−0.13798	4%
		HOMO −> LUMO+3	−0.29556	17%
299.27	0.1892	HOMO−6 −> LUMO	−0.10317	2%
		HOMO−5 −> LUMO+1	−0.38060	29%
		HOMO−4 −> LUMO	0.42517	36%
		HOMO−4 −> LUMO+2	0.10646	2%
		HOMO−2 −> LUMO+1	−0.10136	2%
		HOMO−1 −> LUMO+1	−0.25964	13%
		HOMO−1 −> LUMO+3	−0.11663	3%
294.95	0.0872	HOMO−12 −> LUMO+1	0.19792	8%
		HOMO−5 −> LUMO+1	−0.11608	3%
		HOMO−5 −> LUMO+3	0.16200	5%
		HOMO−4 −> LUMO	0.16340	5%
		HOMO−4 −> LUMO+2	0.14631	4%
		HOMO−1 −> LUMO+1	0.38538	30%
		HOMO−1 −> LUMO+3	0.16692	6%
		HOMO−1 −> 204	−0.10390	2%
		HOMO −> LUMO	0.13658	4%
		HOMO −> LUMO+2	0.34133	23%

		HOMO−6 −> LUMO+1	0.10860	2%
		HOMO−5 −> LUMO+2	0.10135	2%
		HOMO−4 −> LUMO+3	0.11528	3%
		HOMO−3 −> LUMO+1	0.18773	7%
290.77	0.0282	HOMO−2 −> LUMO	−0.11488	3%
		HOMO−2 −> LUMO+2	0.12073	3%
		HOMO−1 −> LUMO	0.50650	51%
		HOMO−1 −> LUMO+2	−0.11891	3%
		HOMO −> LUMO+1	0.27015	15%
		HOMO−13 −> LUMO+1	−0.14959	4%
		HOMO−12 −> LUMO	0.22391	10%
		HOMO−6 −> LUMO+1	−0.11535	3%
		HOMO−5 −> LUMO	−0.16266	5%
		HOMO−5 −> LUMO+2	−0.26031	14%
284.75	0.7605	HOMO−4 −> LUMO+1	0.29106	17%
		HOMO−4 −> LUMO+3	−0.14190	4%
		HOMO−1 −> LUMO	0.15956	5%
		HOMO−1 −> LUMO+2	−0.18457	7%
		HOMO −> LUMO+3	−0.28530	16%
		HOMO −> LUMO+4	0.10870	2%
		HOMO−12 −> LUMO+1	−0.10327	2%
		HOMO−8 −> LUMO+1	0.11535	3%
		HOMO−5 −> LUMO+1	−0.15729	5%
279.22	0.0284	HOMO−3 −> LUMO	0.33189	22%
		HOMO−2 −> LUMO+1	0.47334	45%
		HOMO−2 −> LUMO+3	0.12034	3%
		HOMO−1 −> LUMO+1	0.12889	3%
		HOMO−5 −> LUMO+2	−0.10050	2%
		HOMO−4 −> LUMO+3	−0.11038	2%
		HOMO−3 −> LUMO+1	0.39044	30%
277.80	0.0012	HOMO−2 −> LUMO	0.43346	38%
		HOMO−2 −> LUMO+2	−0.14177	4%
		HOMO−1 −> LUMO	0.14019	4%
		HOMO −> LUMO+1	−0.10787	2%
		HOMO−13 −> LUMO	−0.14317	4%
		HOMO−12 −> LUMO+1	0.14619	4%
272.13	0.1884	HOMO−5 −> LUMO+3	0.22572	10%
		HOMO−4 −> LUMO	−0.13347	4%

		HOMO−4 −> LUMO+2	0.40978	34%
		HOMO−2 −> LUMO+3	0.15317	5%
		HOMO −> LUMO	−0.19173	7%
		HOMO −> LUMO+2	−0.28962	17%
263.73	0.2250	HOMO−12 −> LUMO	0.13668	4%
		HOMO−7 −> LUMO	0.13879	4%
		HOMO−5 −> LUMO	0.27940	16%
		HOMO−5 −> LUMO+2	−0.16134	5%
		HOMO−4 −> LUMO+3	−0.20822	9%
		HOMO−3 −> LUMO+1	−0.21351	9%
		HOMO−2 −> LUMO	−0.10890	2%
		HOMO−2 −> LUMO+2	−0.23173	11%
		HOMO−1 −> LUMO	0.23131	11%
		HOMO−1 −> LUMO+2	0.11612	3%
		HOMO −> LUMO+3	0.26433	14%
254.98	0.0158	HOMO−12 −> LUMO+1	−0.25911	13%
		HOMO−8 −> LUMO+1	0.19505	8%
		HOMO−6 −> LUMO	0.35175	25%
		HOMO−4 −> LUMO	−0.13260	4%
		HOMO−4 −> LUMO+2	0.20976	9%
		HOMO−3 −> LUMO	0.14570	4%
		HOMO−2 −> LUMO+1	−0.22185	10%
		HOMO−2 −> LUMO+3	0.12080	3%
		HOMO−1 −> LUMO+3	−0.18548	7%
		HOMO −> LUMO+2	0.11049	2%
254.30	0.3305	HOMO−12 −> LUMO	−0.19542	8%
		HOMO−8 −> LUMO	0.10262	2%
		HOMO−6 −> LUMO+1	0.26372	14%
		HOMO−5 −> LUMO+2	−0.17675	6%
		HOMO−4 −> LUMO+3	−0.11236	3%
		HOMO−3 −> LUMO+1	0.16690	6%
		HOMO−2 −> LUMO	−0.22903	10%
		HOMO−1 −> LUMO+2	0.31542	20%
		HOMO −> LUMO+3	−0.28796	17%
252.26	0.0194	HOMO−12 −> LUMO	−0.11893	3%
		HOMO−8 −> LUMO	0.22912	10%
		HOMO−7 −> LUMO	0.26712	14%
		HOMO−6 −> LUMO+1	0.35656	25%

	HOMO−4 −> LUMO+1	0.13279	4%
	HOMO−2 −> LUMO+2	0.14781	4%
	HOMO−1 −> LUMO+2	−0.24975	12%
	HOMO −> LUMO+3	0.18383	7%

Cartesian coordinates (B3LYP-D3BJ/def2-SVP, CH_2Cl_2, PCM model): *P*-*M*-Isomerization Process of H1-Me$_2$

(*P*)-H1-Me$_2$
Total energy of the optimized structure *E* = -1197.32682538 Hartree

Symbol	X	Y	Z
C	1.241119	−0.827774	0.018216
C	1.310640	0.648408	−0.047800
N	2.599452	1.021997	−0.259202
B	3.641270	−0.180058	0.014048
C	2.542461	−1.321733	0.247795
C	2.720920	−2.674585	0.594637
C	1.621412	−3.500708	0.731009
C	0.325029	−3.056323	0.359667
C	0.133298	−1.727291	−0.130871
C	0.323085	1.672396	0.127893
C	0.659489	2.973866	−0.358225
C	1.997131	3.260407	−0.723856
C	2.950101	2.282423	−0.577653
C	−0.797151	−3.945464	0.467294
C	−2.041196	−3.570274	0.054410
C	−2.235229	−2.319012	−0.617904
C	−1.132530	−1.422027	−0.776840
C	−0.968286	1.490967	0.771569
C	−1.959448	2.511055	0.626366
C	−1.626334	3.737533	−0.037661
C	−0.354210	3.983554	−0.459473
C	−1.276504	0.388014	1.601544
C	−2.530904	0.242767	2.169337
C	−3.536502	1.201761	1.938643
C	−3.243698	2.328941	1.192158
C	−3.492424	−1.987310	−1.181072
C	−3.655087	−0.840626	−1.936543
C	−2.543555	−0.009300	−2.186311
C	−1.313997	−0.301331	−1.623867

C	4.516010	0.218847	1.330482
C	4.551589	−0.456211	−1.307369
H	3.725511	−3.057574	0.795097
H	1.734242	−4.528614	1.086428
H	2.272547	4.258616	−1.065454
H	4.014498	2.460390	−0.735750
H	−0.630114	−4.933773	0.902876
H	−2.896089	−4.240651	0.170899
H	−2.403000	4.498540	−0.145101
H	−0.082110	4.949448	−0.889885
H	−0.512669	−0.355792	1.812472
H	−2.737570	−0.624125	2.800502
H	−4.530143	1.069914	2.372372
H	−3.995005	3.108796	1.046740
H	−4.331200	−2.670407	−1.024620
H	−4.629268	−0.597244	−2.366857
H	−2.649358	0.866682	−2.830104
H	−0.464600	0.337172	−1.855588
H	5.175839	1.085795	1.146264
H	3.889443	0.456214	2.208010
H	5.168486	−0.626261	1.612728
H	5.196552	−1.338104	−1.148721
H	3.947457	−0.651733	−2.210590
H	5.222664	0.393194	−1.530989

TS
Total energy of the optimized structure *E* = -1197.26896773 Hartree

Symbol	X	Y	Z
H	−3.371296	1.190056	2.760546
H	−5.020142	2.094776	1.110753
C	−3.038608	1.492607	1.765734
C	−3.963078	2.020248	0.844946
C	−1.705990	1.354786	1.413530
C	−3.513311	2.511977	−0.369842
H	−4.204500	3.009990	−1.054715
C	−1.230283	1.764699	0.150001
C	−2.138783	2.469420	−0.704055
C	0.186561	1.728905	−0.172601

C	−1.614901	3.271633	−1.772722
H	−2.309394	3.780120	−2.445616
C	−0.275309	3.518554	−1.824591
H	0.130456	4.253834	−2.524190
C	0.652289	2.818469	−0.975459
C	1.196725	0.811992	0.303834
C	2.010001	3.217101	−0.915365
C	2.528372	1.278295	0.406494
C	2.900610	2.531507	−0.110572
C	3.670395	0.020435	2.505713
H	4.233598	0.886692	2.894221
H	2.693860	0.004352	3.020153
H	4.224325	−0.887645	2.804968
C	4.958279	0.081730	0.117709
H	5.595844	−0.764141	0.432674
H	5.525750	0.997731	0.360301
H	4.867952	0.040737	−0.982339
C	1.253359	−0.690823	0.279476
C	3.039946	−2.220686	−0.130689
C	0.326323	−1.693830	−0.181645
H	2.685127	−3.831656	−1.506710
C	2.257280	−2.998908	−0.947625
C	0.873969	−2.718815	−1.014518
H	0.460137	−4.149718	−2.601419
C	0.004212	−3.463594	−1.884539
H	−2.005270	−3.851528	−2.504829
C	−1.348322	−3.311561	−1.818849
C	−1.934001	−2.574201	−0.734540
C	−1.084635	−1.824236	0.138690
H	−3.947057	−3.273941	−1.104929
C	−3.299640	−2.736602	−0.407741
C	−1.590526	−1.473746	1.406250
H	−0.907100	−1.061242	2.145452
C	−3.787735	−2.308220	0.816806
C	−2.910527	−1.724791	1.747874
H	−4.835276	−2.471983	1.078972
H	−3.268747	−1.465694	2.745777
H	4.096461	−2.419942	0.043341

H	−0.985586	0.986267	2.141125
H	3.932871	2.875485	−0.009361
H	2.330435	4.081516	−1.503356
B	3.527653	0.115152	0.880156
N	2.557608	−1.073414	0.386883

Cartesian coordinates (B3LYP-D3BJ/def2-SVP, CH_2Cl_2, PCM model): *P*-*M*-Isomerization Process of H2-Me$_4$

(*M*,*M*)-H2-Me$_4$

Total energy of the optimized structure *E* = -1548.34936954 Hartree

Symbol	X	Y	Z
C	1.196680	1.332512	0.133213
C	1.217167	2.754523	0.082002
C	−0.000002	3.439534	0.000000
C	−1.217170	2.754522	−0.082002
C	−1.196683	1.332510	−0.133216
C	−0.000001	0.621708	−0.000003
C	2.571859	0.802979	0.168407
C	−2.571861	0.802976	−0.168410
C	3.069307	−0.540293	0.215415
C	4.405150	−0.741188	−0.250059
C	5.249763	0.378032	−0.452044
C	4.750332	1.640583	−0.249558
N	3.440832	1.830981	0.006837
C	2.321849	−1.704638	0.671595
C	2.796694	−3.007860	0.322238
C	4.073402	−3.149335	−0.316456
C	4.876095	−2.067911	−0.524993
N	−3.440835	1.830977	−0.006839
C	−4.750334	1.640577	0.249557
C	−5.249764	0.378026	0.452043
C	−4.405150	−0.741193	0.250059
C	−3.069307	−0.540297	−0.215416
C	0.898800	−4.030909	1.452290
C	0.499727	−2.759870	1.909145
B	2.726857	3.290291	0.065122
B	−2.726861	3.290288	−0.065121
C	3.102119	4.135262	−1.273881
C	−3.195167	4.021762	−1.444764

C	1.203787	−1.627274	1.536254
C	2.047554	−4.150728	0.692286
C	−4.876092	−2.067916	0.524993
C	−4.073398	−3.149340	0.316457
C	−2.796691	−3.007863	−0.322238
C	−2.321847	−1.704640	−0.671595
C	−2.047549	−4.150729	−0.692287
C	−0.898794	−4.030908	−1.452290
C	−0.499722	−2.759869	−1.909143
C	−1.203784	−1.627274	−1.536253
H	−0.323480	−4.917058	−1.726929
H	0.365128	−2.663622	−2.568766
H	−0.903338	−0.661384	−1.936294
H	−2.409734	−5.134927	−0.385796
H	−4.419288	−4.153876	0.571690
H	−5.883313	−2.186274	0.929581
H	−6.287172	0.234862	0.756182
H	−0.365122	−2.663625	2.568769
H	0.903341	−0.661385	1.936297
H	0.323487	−4.917060	1.726930
H	2.409740	−5.134925	0.385795
H	4.419294	−4.153871	−0.571689
H	5.883316	−2.186268	−0.929580
H	6.287172	0.234869	−0.756183
H	5.358398	2.540914	−0.343137
H	0.000001	−0.459070	−0.000007
H	−0.000003	4.535408	0.000001
H	−5.358402	2.540908	0.343135
H	−2.979673	3.419325	−2.344729
H	−4.276652	4.250478	−1.446630
H	−2.662525	4.982281	−1.558845
H	4.170176	4.417308	−1.313220
H	2.865775	3.591635	−2.205721
H	2.524819	5.076409	−1.292692
C	−3.102124	4.135256	1.273884
H	−2.865781	3.591627	2.205723
H	−2.524823	5.076403	1.292697
H	−4.170180	4.417303	1.313223

C	3.195161	4.021762	1.444767
H	4.276646	4.250481	1.446633
H	2.662517	4.982279	1.558851
H	2.979670	3.419322	2.344731

TS3
Total energy of the optimized structure *E* = -1548.32645708 Hartree

Symbol	X	Y	Z
C	2.466497	-0.916596	0.092384
C	3.268889	0.293840	-0.119909
C	4.699796	0.129892	-0.023962
C	5.285149	-1.104720	0.324367
C	4.476300	-2.189469	0.474625
N	3.147499	-2.078446	0.322239
C	2.828862	1.656481	-0.479923
C	3.794809	2.663153	-0.835725
C	5.194061	2.398247	-0.745257
C	5.622917	1.187522	-0.315485
C	2.049299	4.315853	-1.272090
C	1.095378	3.368706	-0.869367
B	2.276192	-3.422921	0.321712
C	2.752112	-4.352422	-0.931921
C	1.491277	2.096518	-0.490679
C	3.383100	3.956192	-1.239973
H	0.732717	1.425731	-0.157456
H	1.747754	5.318789	-1.581280
H	4.157347	4.676708	-1.513662
H	5.900852	3.189260	-1.005046
H	6.687811	0.971694	-0.213824
H	6.366734	-1.191992	0.425134
H	4.851190	-3.188690	0.694079
H	3.770012	-4.761420	-0.798425
H	2.731637	-3.813661	-1.895627
H	2.070838	-5.216125	-1.027235
C	2.410025	-4.172642	1.764130
H	3.424454	-4.573467	1.943421
H	1.718453	-5.032440	1.799801
H	2.155452	-3.515235	2.614123

C	0.876007	-2.724449	0.118188
C	-0.366568	-3.355518	0.025508
C	-1.552326	-2.627407	-0.055503
C	-1.442840	-1.219266	0.044467
B	-3.071919	-3.060290	-0.318791
C	-2.776181	-0.599389	-0.052058
N	-3.676459	-1.551046	-0.399917
C	-3.287811	-3.816833	-1.742497
C	-3.791148	-3.815328	0.933550
C	-3.201846	0.763093	0.061250
C	-4.923757	-1.250262	-0.813420
H	-2.872964	-3.253430	-2.596803
H	-4.353247	-4.016038	-1.958903
H	-2.779475	-4.796890	-1.723243
H	-4.872055	-3.968513	0.760489
H	-3.343025	-4.814552	1.075258
H	-3.679575	-3.268648	1.886405
C	-4.448292	1.094695	-0.552073
C	-2.475463	1.819581	0.753203
C	-5.314755	0.056189	-0.974690
H	-5.567286	-2.096292	-1.057076
C	-4.810863	2.470467	-0.735754
C	-2.844973	3.180012	0.512138
C	-1.498687	1.572393	1.748858
H	-6.289880	0.294151	-1.400817
C	-4.002994	3.470574	-0.283615
H	-5.741200	2.695374	-1.261270
C	-2.124055	4.225559	1.139850
C	-0.829064	2.610043	2.375660
H	-1.285945	0.548058	2.047926
H	-4.264216	4.516804	-0.459257
C	-1.115294	3.949264	2.044585
H	-2.402802	5.258532	0.918575
H	-0.567790	4.762142	2.525988
C	0.997967	-1.297992	0.066795
C	-0.203496	-0.567346	0.066701
H	-0.397311	-4.450719	0.010052
H	-0.264179	0.502184	0.099837

H	-0.080074	2.384115	3.137527
H	0.034943	3.625273	-0.833697

LM2
(**(*P*,*M*)-H2-Me$_4$**)
Total energy of the optimized structure *E* = -1548.34146345 Hartree

Symbol	X	Y	Z
C	2.692939	−0.460401	0.176247
C	3.160581	0.832111	−0.249320
C	4.514016	0.903230	−0.712133
C	5.403301	−0.167064	−0.461244
C	4.924294	−1.297673	0.146056
N	3.608023	−1.442377	0.389900
C	2.378206	2.061654	−0.291987
C	2.843392	3.151859	−1.096831
C	4.140498	3.090132	−1.703039
C	4.972194	2.042720	−1.452399
C	0.900671	4.481552	−0.484852
C	0.507320	3.467486	0.407861
B	2.968690	−2.857638	0.828400
C	3.524438	−4.044553	−0.135390
C	1.232917	2.290457	0.505431
C	2.067911	4.330199	−1.209716
H	−0.370013	3.609472	1.035890
H	0.934079	1.546558	1.240191
H	0.309551	5.395944	−0.569573
H	2.430317	5.133397	−1.855718
H	4.473450	3.934191	−2.311513
H	5.994695	2.031744	−1.834941
H	6.450950	−0.093131	−0.754306
H	5.554904	−2.152078	0.392143
H	4.593568	−4.270078	0.030078
H	3.391509	−3.823636	−1.209298
H	2.971118	−4.977187	0.073507
C	3.304996	−3.100000	2.407553
H	4.389702	−3.224023	2.581010
H	2.813340	−4.022792	2.761804
H	2.955758	−2.274115	3.051893
C	1.450174	−2.451459	0.559871

C	0.331066	−3.280819	0.449728
C	−0.918928	−2.763556	0.102908
C	−1.041323	−1.353285	−0.001597
B	−2.276803	−3.477051	−0.379623
C	−2.426399	−1.002066	−0.330697
N	−3.076453	−2.106219	−0.766851
C	−2.069678	−4.397395	−1.703367
C	−3.132149	−4.209150	0.798091
C	−3.120705	0.243008	−0.267251
C	−4.264704	−2.031691	−1.399238
H	−1.555724	−3.863196	−2.521795
H	−3.026710	−4.780014	−2.103260
H	−1.453933	−5.280031	−1.455753
H	−4.114599	−4.566585	0.439585
H	−2.580417	−5.093820	1.162069
H	−3.312184	−3.554722	1.669005
C	−4.299217	0.353725	−1.064280
C	−2.785792	1.333625	0.638249
C	−4.851668	−0.813459	−1.650471
H	−4.700052	−2.978683	−1.720777
C	−4.933547	1.631645	−1.215020
C	−3.481656	2.574589	0.499826
C	−1.942604	1.170038	1.766188
H	−5.768795	−0.751414	−2.237206
C	−4.503749	2.709554	−0.499478
H	−5.780701	1.715031	−1.898972
C	−3.241514	3.622625	1.422715
C	−1.767885	2.189302	2.686565
H	−1.456072	0.212809	1.940194
H	−4.991564	3.680264	−0.615875
C	−2.402535	3.435613	2.506058
H	−3.769146	4.569767	1.287905
H	−2.243994	4.240772	3.226495
C	1.333580	−1.053911	0.283023
C	0.070974	−0.499209	0.019176
H	0.457346	−4.362441	0.569810
H	−0.062521	0.551698	−0.219082
H	−1.130763	2.023320	3.557850

TS2

Total energy of the optimized structure *E* = -1548.33913119 Hartree

Symbol	X	Y	Z
C	−2.562721	0.793271	−0.055305
C	−3.099518	−0.488953	−0.387763
C	−4.232253	−0.505231	−1.257029
C	−4.936874	0.699112	−1.501159
C	−4.508796	1.855277	−0.895890
N	−3.337998	1.891127	−0.228029
C	−2.631546	−1.754040	0.152483
C	−3.103346	−2.969985	−0.433516
C	−4.070828	−2.919811	−1.491905
C	−4.660019	−1.742155	−1.844104
C	−1.965496	−4.265460	1.281678
C	−1.590794	−3.066793	1.919631
B	−2.677483	3.232672	0.398322
C	−2.720137	4.443128	−0.685518
C	−1.883853	−1.839859	1.351048
C	−2.707884	−4.213490	0.116507
H	−1.076238	−3.103540	2.879151
H	−1.584179	−0.927354	1.864658
H	−1.697902	−5.227484	1.723840
H	−3.050050	−5.132516	−0.365261
H	−4.391721	−3.858708	−1.949432
H	−5.473859	−1.717410	−2.571676
H	−5.830372	0.697445	−2.126356
H	−5.048875	2.799499	−0.969690
H	−3.747159	4.797231	−0.889853
H	−2.261274	4.171034	−1.652498
H	−2.160391	5.311427	−0.295398
C	−3.479152	3.571631	1.778528
H	−4.531515	3.848974	1.585434
H	−3.006580	4.430526	2.286707
H	−3.481627	2.728923	2.492149
C	−1.213184	2.616637	0.611649
C	0.000401	3.278759	0.802247
C	1.213889	2.616271	0.612289
C	1.205557	1.216309	0.346612

B	2.678457	3.231898	0.398871
C	2.562841	0.792791	−0.055549
N	3.337940	1.890660	−0.229085
C	2.721781	4.443998	−0.683007
C	3.480830	3.568180	1.779427
C	3.099656	−0.489477	−0.387616
C	4.508155	1.854814	−0.897914
H	2.264568	4.173080	−1.651105
H	3.748830	4.799249	−0.885219
H	2.160764	5.311267	−0.292434
H	4.533194	3.845543	1.586387
H	3.008680	4.426279	2.289344
H	3.483318	2.724202	2.491549
C	4.232094	−0.506058	−1.257179
C	2.631224	−1.754469	0.152548
C	4.936279	0.698349	−1.502582
H	5.047670	2.799263	−0.972963
C	4.659759	−1.743196	−1.843948
C	3.102403	−2.970537	−0.433649
C	1.883967	−1.840018	1.351370
H	5.829366	0.696533	−2.128364
C	4.070077	−2.920650	−1.491875
H	5.473947	−1.718819	−2.571144
C	2.706441	−4.213882	0.116316
C	1.590302	−3.066861	1.919834
H	1.584818	−0.927426	1.865135
H	4.390792	−3.859660	−1.949299
C	1.964172	−4.265634	1.281565
H	3.048047	−5.132985	−0.365696
H	1.696030	−5.227559	1.723606
C	−1.205107	1.216728	0.345831
C	0.000121	0.500531	0.228332
H	0.000533	4.360890	0.972302
H	0.000041	−0.555096	−0.043879
H	1.076034	−3.103514	2.879510

LM1

(***P*,*M*)-H2-Me$_4$**)

Total energy of the optimized structure *E* = -1548.34146346 Hartree

Symbol	X	Y	Z
C	2.426422	−1.002043	−0.330694
C	3.120716	0.243036	−0.267224
C	4.299237	0.353773	−1.064238
C	4.851708	−0.813400	−1.650432
C	4.264750	−2.031641	−1.399222
N	3.076490	−2.106185	−0.766854
C	2.785775	1.333644	0.638275
C	3.481616	2.574622	0.499859
C	4.503721	2.709604	−0.499428
C	4.933550	1.631702	−1.214965
C	2.402433	3.435637	2.506062
C	1.767816	2.189309	2.686572
B	2.276853	−3.477027	−0.379653
C	2.069742	−4.397356	−1.703409
C	1.942578	1.170042	1.766206
C	3.241429	3.622659	1.422734
H	1.130680	2.023319	3.557845
H	1.456068	0.212803	1.940211
H	2.243857	4.240799	3.226489
H	3.769040	4.569812	1.287925
H	4.991521	3.680323	−0.615820
H	5.780711	1.715104	−1.898905
H	5.768841	−0.751339	−2.237154
H	4.700107	−2.978626	−1.720769
H	3.026777	−4.779950	−2.103316
H	1.555772	−3.863155	−2.521826
H	1.454016	−5.280009	−1.455805
C	3.132193	−4.209130	0.798063
H	4.114645	−4.566569	0.439564
H	2.580457	−5.093797	1.162042
H	3.312225	−3.554699	1.668975
C	0.918965	−2.763551	0.102875
C	−0.331024	−3.280829	0.449690
C	−1.450140	−2.451480	0.559843

C	−1.333561	−1.053929	0.283003
B	−2.968647	−2.857672	0.828398
C	−2.692925	−0.460427	0.176247
N	−3.607998	−1.442409	0.389924
C	−3.524407	−4.044580	−0.135394
C	−3.304924	−3.100049	2.407556
C	−3.160588	0.832080	−0.249316
C	−4.924276	−1.297715	0.146113
H	−3.391517	−3.823642	−1.209301
H	−4.593527	−4.270128	0.030106
H	−2.971063	−4.977208	0.073470
H	−4.389628	−3.224071	2.581024
H	−2.813265	−4.022844	2.761794
H	−2.955676	−2.274168	3.051896
C	−4.514033	0.903185	−0.712099
C	−2.378228	2.061635	−0.292006
C	−5.403306	−0.167113	−0.461180
H	−5.554875	−2.152123	0.392218
C	−4.972238	2.042664	−1.452368
C	−2.843437	3.151823	−1.096860
C	−1.232937	2.290465	0.505398
H	−6.450963	−0.093187	−0.754215
C	−4.140555	3.090076	−1.703043
H	−5.994749	2.031673	−1.834884
C	−2.067971	4.330169	−1.209774
C	−0.507351	3.467500	0.407800
H	−0.934088	1.546583	1.240172
H	−4.473524	3.934125	−2.311522
C	−0.900722	4.481546	−0.484927
H	−2.430392	5.133354	−1.855782
H	−0.309613	5.395942	−0.569670
C	1.041345	−1.353280	−0.001615
C	−0.070960	−0.499216	0.019156
H	−0.457292	−4.362452	0.569771
H	0.062524	0.551693	−0.219097
H	0.369987	3.609506	1.035818

TS1

Total energy of the optimized structure *E* = -1548.32645711 Hartree

Symbol	X	Y	Z
C	2.466481	0.916485	0.092685
C	3.268797	-0.293993	-0.119614
C	4.699704	-0.130150	-0.023517
C	5.285090	1.104361	0.325123
C	4.476305	2.189173	0.475315
N	3.147531	2.078277	0.322652
C	2.828700	-1.656522	-0.479955
C	3.794605	-2.663101	-0.836141
C	5.193868	-2.398316	-0.745482
C	5.622780	-1.187754	-0.315268
C	2.049026	-4.315571	-1.273060
C	1.095163	-3.368575	-0.869842
B	2.276334	3.422862	0.321600
C	2.752519	4.351801	-0.932349
C	1.491112	-2.096543	-0.490683
C	3.382841	-3.955967	-1.240869
H	0.732587	-1.425920	-0.157029
H	1.747431	-5.318362	-1.582671
H	4.157054	-4.676401	-1.514869
H	5.900621	-3.189292	-1.005489
H	6.687680	-0.972037	-0.213432
H	6.366665	1.191535	0.426086
H	4.851243	3.188354	0.694869
H	3.770470	4.760690	-0.798902
H	2.732049	3.812675	-1.895849
H	2.071379	5.215566	-1.028044
C	2.410092	4.173138	1.763724
H	3.424523	4.573983	1.942957
H	1.718564	5.032989	1.798986
H	2.155396	3.516090	2.613958
C	0.876105	2.724438	0.118123
C	-0.366458	3.355522	0.025381
C	-1.552236	2.627427	-0.055536
C	-1.442796	1.219291	0.044568
B	-3.071815	3.060341	-0.318833

C	-2.776143	0.599439	-0.052005
N	-3.676386	1.551089	-0.399948
C	-3.287690	3.816909	-1.742520
C	-3.791054	3.815364	0.933515
C	-3.201826	-0.763028	0.061328
C	-4.923688	1.250307	-0.813451
H	-2.873015	3.253426	-2.596858
H	-4.353101	4.016332	-1.958842
H	-2.779150	4.796863	-1.723304
H	-4.871966	3.968509	0.760457
H	-3.342964	4.814603	1.075218
H	-3.679462	3.268691	1.886372
C	-4.448237	-1.094647	-0.552051
C	-2.475468	-1.819498	0.753325
C	-5.314695	-0.056146	-0.974691
H	-5.567197	2.096338	-1.057158
C	-4.810753	-2.470429	-0.735778
C	-2.844890	-3.179937	0.512182
C	-1.498838	-1.572277	1.749114
H	-6.289822	-0.294113	-1.400811
C	-4.002853	-3.470518	-0.283652
H	-5.741069	-2.695358	-1.261321
C	-2.123986	-4.225462	1.139947
C	-0.829234	-2.609909	2.375967
H	-1.286213	-0.547935	2.048246
H	-4.264010	-4.516753	-0.459362
C	-1.115347	-3.949135	2.044809
H	-2.402650	-5.258445	0.918610
H	-0.567847	-4.761998	2.526243
C	0.997998	1.297970	0.066927
C	-0.203469	0.567346	0.066952
H	-0.397191	4.450723	0.009849
H	-0.264131	-0.502176	0.100384
H	-0.080375	-2.383967	3.137958
H	0.034730	-3.625154	-0.834134

9. References

[1] J. R. Lakowicz, Principles of Fluorescence Spectroscopy, *2nd ed.*, Kluwer Academic/Plenum, New York, **1999**.
[2] I. B. Berlman in *Handbook of Fluorescence Spectra of Aromatic Molecules, 2nd ed*., *Academic Press,* New York, **1971**, p. 399.
[3] A. J. Fry in *Laboratory Techniques in Electroanalytical Chemistry*, *2nd ed*. (Eds.: P. T. Kissinger, W. R. Heineman), Marcel Dekker Ltd, New York, **1996**, p. 481.
[4] V. Mamane, F. Louërat, J. Iehl, M. Abboud, Y. Fort, *Tetrahedron* **2008**, *64*, 10699-10705.
[5] R. Sun, X. Yang, Q. Li, K. Xu, J. Tang, X. Zheng, M. Yuan, H. Fu, R. Li, H. Chen, *Org. Lett.* **2019**, *21*, 9425-9429.
[6] J. Zheng, S. L. You, *Angew. Chem. Int. Ed.* **2014**, *53*, 13244-13247; *Angew. Chem.* **2014**, *126*, 13460-13463.
[7] A. Fürstner, V. Mamane, *J. Org. Chem.* **2002**, *67*, 6264-6267.
[8] M. Kastler, J. Schmidt, W. Pisula, D. Sebastiani, K. Müllen, *J. Am. Chem. Soc.* **2006**, *128*, 9526-9534.
[9] A. Gosset, Z. Xu, F. Maurel, L. M. Chamoreau, S. Nowak, G. Vives, C. Perruchot, V. Heitz, H. P. Jacquot De Rouville, *New J. Chem.* **2018**, *42*, 4728–4734.
[10] G. M. Sheldrick, *Acta Crystallogr.* A **2008**, *64*, 112-122.
[11] Gaussian 09, Revision D.01, M. J. Frisch, G. W. Trucks, H. B. Schlegel, G. E. Scuseria, M. A. Robb, J. R. Cheeseman, G. Scalmani, V. Barone, B. Mennucci, G. A. Petersson, H. Nakatsuji, M. Caricato, X. Li, H. P. Hratchian, A. F. Izmaylov, J. Bloino, G. Zheng, J. L. Sonnenberg, M. Hada, M. Ehara, K. Toyota, R. Fukuda, J. Hasegawa, M. Ishida, T. Nakajima, Y. Honda, O. Kitao, H. Nakai, T. Vreven, J. A. Jr. Montgomery, J. E. Peralta, F. Ogliaro, M. Bearpark, J. J. Heyd, E. Brothers, K. N. Kudin, V. N. Staroverov, T. Keith, R. Kobayashi, J. Normand, K. Raghavachari, A. Rendell, J. C. Burant, S. S. Iyengar, J. Tomasi, M. Cossi, N. Rega, J. M. Millam, M. Klene, J. E. Knox, J. B. Cross, V. Bakken, C. Adamo, J. Jaramillo, R. Gomperts, R. E. Stratmann, O. Yazyev, A. J. Austin, R. Cammi, C. Pomelli, J. W. Ochterski, R. L. Martin, K. Morokuma, V. G. Zakrzewski, G. A. Voth, P. Salvador, J. J. Dannenberg, S. Dapprich, A. D. Daniels, O. Farkas, J. B. Foresman, J. V. Ortiz, J. Cioslowski, D. J. Fox, Gaussian, Inc., Wallingford CT, 2013.
[12] a) A. D. Becke, *Phys. Rev. A* **1988**, *38*, 3098-3100; b) C. Lee, W. Yang, R. G. Parr, *Phys. Rev. B* **1988**, *37*, 785-789; c) A. D. Becke, *J. Chem. Phys.* **1993**, *98*, 5648-5652.
[13] a) A. Schäfer, C. Huber, R. Ahlrichs, *J. Chem. Phys.* **1994**, *100*, 5829-5835; b) F. Weigend, R. Ahlrichs, *Phys. Chem. Chem. Phys*. **2005**, *7*, 3297-3305; c) F. Weigend, *Phys. Chem. Chem. Phys.* **2006**, *8*, 1057-1065.
[14] S. Grimme, S. Ehrlich, L. Goerigk, *J. Comput. Chem.* **2011**, *32*, 1456-1465.
[15] T. Yanai, D. P. Tew, N. C. Handy, *Chem. Phys. Lett*. **2004**, *393*, 51-57.
[16] GaussView, Version 5, R. Dennington, T. Keith, J. Millam, Semichem Inc., Shawnee Mission KS, 2009.
[17] N. M. O'Boyle, A. L. Tenderholt, K. M. Langner, *J. Comp. Chem.* **2008**, *29*, 839-845.
